# A dynamic interaction process between KaiA and KaiC is critical to the cyanobacterial circadian oscillator

**DOI:** 10.1038/srep25129

**Published:** 2016-04-26

**Authors:** Pei Dong, Ying Fan, Jianqiang Sun, Mengting Lv, Ming Yi, Xiao Tan, Sen Liu

**Affiliations:** 1 Hubei Key Laboratory of Tumor Microenvironment and Immunotherapy, China Three Gorges University, Yichang 443002, China; 2 College of Medical Science, China Three Gorges University, Yichang 443002, China; 3 School of Statistics, Shandong Institute of Business and Technology, Yantai, 264005, China; 4 Department of Physics, College of Sciences, Huazhong Agricultural University, Wuhan 430070, China

## Abstract

The core circadian oscillator of cyanobacteria consists of three proteins, KaiA, KaiB, and KaiC. This circadian oscillator could be functionally reconstituted *in vitro* with these three proteins, and therefore has been a very important model in circadian rhythm research. KaiA can bind to KaiC and then stimulate its phosphorylation, but their interaction mechanism remains elusive. In this study, we followed the “second-site suppressor” strategy to investigate the interaction mechanism of KaiA and KaiC. Using protein sequence analyses, we showed that there exist co-varying residues in the binding interface of KaiA and KaiC. The followed mutagenesis study verified that these residues are important to the functions of KaiA and KaiC, but their roles could not be fully explained by the reported complex structures of KaiA and KaiC derived peptides. Combining our data with previous reports, we suggested a dynamic interaction mechanism in KaiA-KaiC interaction, in which both KaiA and the intrinsically disordered tail of KaiC undergo significant structural changes through conformational selection and induced fit during the binding process. At last, we presented a mathematic model to support this hypothesis and explained the importance of this interaction mechanism for the KaiABC circadian oscillator.

Circadian rhythms are natural rhythms generated by biological clocks with a period of approximately 24 h. Existing in near all organisms, circadian rhythms enable the organisms to anticipate and then adapt to the daily environment changes^1^. The importance of circadian rhythms is underscored by the findings showing that disturbance to circadian rhythms causes fitness disadvantages and health problems^2^,^3^,^4^,^5^. As the simplest organism having an endogenous circadian clock, cyanobacteria have been ideal organisms for circadian rhythm research^6^.

At the molecular level, circadian rhythms are regulated by molecular clocks composed of defined sets of genes and their products (RNAs and proteins). A typical circadian clock system is composed of three coupled units: a sensory input unit, an oscillation-generating unit (oscillator), and an output unit^7^. At the center of a circadian clock, the circadian oscillator turns the environmental cues into the changes of gene transcriptions and translations. In a very long time, transcription and translation-based negative feedback loops (TTFLs) were considered to be the sole mechanism of circadian oscillators, except that a non-TTFL circadian oscillator was found in cyanobacteria^8^. However, recent studies have proved that non-TTFL circadian oscillators exist in all domains of life including human, and are of outstanding importance^1^,^9^,^10^. As the best known non-TTFL circadian oscillator, the cyanobacterial circadian oscillator consists of three clock proteins, KaiA, KaiB, and KaiC^11^. In this oscillator, KaiC is an enzyme that undergoes auto-phosphorylation/auto-dephosphorylation orderly at two sites (T432 and S431). KaiA stimulates the phosphorylation of KaiC, whereas KaiB antagonizes KaiA to induce the dephosphorylation phase of KaiC. It was shown that, in the presence of ATP, these three clock proteins can generate circadian oscillations *in vitro*, with the phosphorylation/dephosphorylation of KaiC oscillating with a period of ~24 h^12^,^13^. Further studies showed that the major functional forms of these three proteins are KaiA dimer, KaiB dimer/tetramer, and KaiC hexamer, and these functional forms were supported well by structural studies^14^,^15^.

Although much has been done to understand the molecular mechanism of the KaiABC system, the complex structures between these three clock proteins remain mysterious. It was demonstrated that these three proteins could form different hetero-oligomers^16^,^17^, but no structures with enough details of these hetero-oligomers have been determined thus far. For example, in electron microscopy (EM) analysis, KaiA was found to bind with KaiC via the interactions between the C-terminal domains of KaiA homo-dimer and an intrinsically disordered region (IDR) - the C-terminal tail of KaiC^18^. KaiA could adopt a “tethered” orientation or an “engaged” orientation binding with the full-length KaiC. In the tethered model, KaiA was trapped by the KaiC C-terminus, but did not have direct contacts with the dome structure of the CII domain of KaiC (KaiC-CII). In the engaged model, KaiA was brought close to and had direct contacts with the dome structure of KaiC-CII. Nonetheless, the resolutions of the EM models were too low to reveal more details of KaiA-KaiC interaction, including the structural details coupling the transition of these two binding models. Alternatively, a KaiC C-terminus derived peptide was used and its complex structure with the C-terminal domains of KaiA homo-dimer was determined by NMR^19^. In this model, the KaiC C-terminus derived peptide adopted a random coil conformation fitting in the concaved space between the C-terminal domains of KaiA homo-dimer.

In this paper, we set out to understand the interaction details and mechanism between KaiA and KaiC. We chose the KaiABC clock proteins from the widely used cyanobacterium *Synechococcus elongatus* PCC 7942 (S. e. PCC 7942) for experimental study. By combining multiple sequence alignments, evolutionary information, computational modeling of protein structures, and site-directed mutagenesis, we comprehensively studied the interaction interface between the KaiA C-terminal domain and the KaiC C-terminal tail, and showed that the interaction between KaiA and KaiC has a dynamic mechanism, in which both KaiA and the C-terminal tail of KaiC have significant conformational changes upon binding. Finally, a mathematic model was used to demonstrate that this binding mechanism is critical to the oscillation of the KaiABC system.

## Results

### Evolution and co-variance analyses of the critical residues for KaiA-KaiC interaction

To pinpoint the critical residues for KaiA-KaiC interaction, we reasoned that these residues should either be conserved or co-vary across different species. So we searched the NCBI protein sequence database and collected the sequences of these three clock proteins (KaiA, KaiB, and KaiC) from 65 cyanobacterial species (these three proteins co-exist in each species). The full-length protein sequences were then aligned in MEGA v5.05^20^ respectively, and manually adjusted to keep the intact of the secondary structural elements according to the known structure of the respective protein of S. e. PCC 7942 (Supplementary Fig. 1A,B,C). The sequence alignments showed that the overall sequences of KaiB and KaiC were highly conserved, but KaiA sequences were only quite conserved in the last two helices of the C-terminal domain (α-helices 9 and 10 in Supplementary Fig. 1). According to the NMR structure (PDB ID: 1SUY) of KaiA C-terminal domain (α-helices 7, 8, 9, and 10) and KaiC C-terminus derived peptide from *Thermosynechococcus elongatus* BP-1 (T. e. BP-1), these two sequence-conserved helices of KaiA were involved in the interactions with the C-terminal tail of KaiC, which is the way KaiA binds with KaiC^21^. Therefore, although there was no study directly proving that all of these 65 species have the independent KaiABC circadian oscillator, the conservation in all these three proteins indicated that these clock proteins were likely correlated evolutionarily.

**Figure 1:**
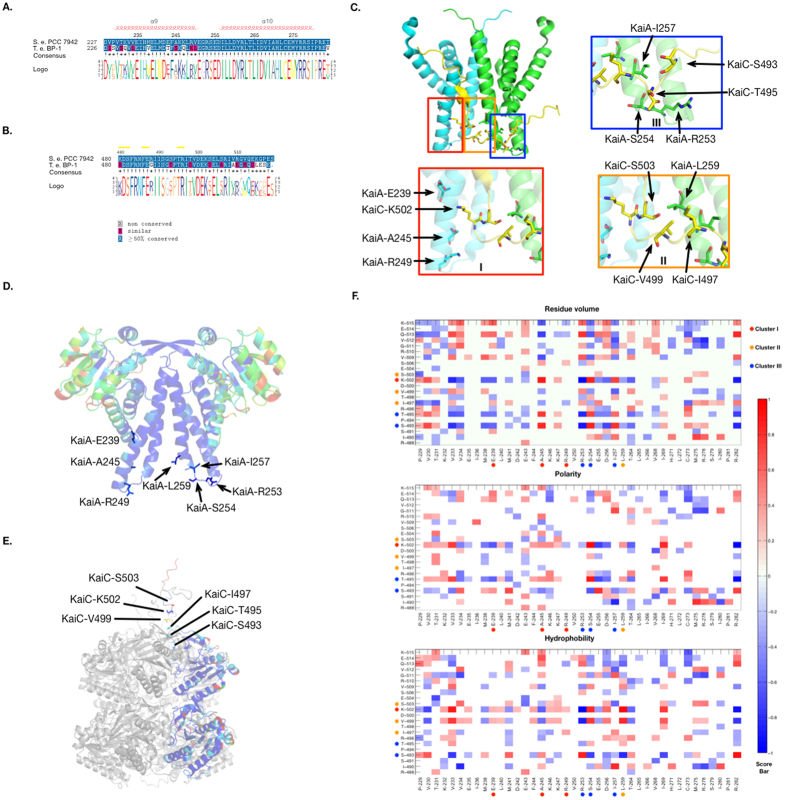
The sequence analyses of the clock proteins. (**A**) The sequence alignment of KaiA proteins of S.e. PCC 7942 and T. e. BP-1. Only the C-terminal domains (helices 9, and 10) were shown. See Supplementary Fig. 1A for the alignment of the full-length sequences. All the sequence alignments in this paper were rendered with STRAP^57^. (**B**) The sequence alignment of KaiC C-terminal tails of S. e. PCC 7942 and T. e. BP-1. See Supplementary Fig. 1C for the alignment of the full-length sequences. (**C**) The NMR structure between KaiA C-terminal domains (in cyan and green) and a KaiC C-terminus derived peptide (in yellow, PDB ID: 1SV1) of T. e. BP-1. Three “co-varying residue clusters” were identified. (**D**) The Relative Evolution Rates (RERs) of KaiA were calculated and shown in colors on KaiA of S. e. PCC 7942 (PDB ID: 1R8J). The color is gradiently changed from blue (small RERs) to red (large RERs). See Table 1 and Supplementary Fig. 1E for values. (**E**) The Relative Evolution Rates (RERs) of KaiC were calculated and shown in colors on KaiC of S. e. PCC 7942 (PDB ID: 3S1A). See Table 1 and Supplementary Fig. 1F for values. (**F**) The calculated residue-residue pair positional correlations of the identified co-varying residues in the three clusters. Red means two residues are positively correlated, and blue means negative correlation.

Since both the KaiA C-terminal domains (especially the helices 9 and 10) and the KaiC C-terminal tails were highly similar in sequence between S. e. PCC 7942 and T. e. BP-1 (Fig. 1A,B), we supposed that their binding modes should be similar too. To identify the key interaction residues between KaiA C-terminal domain and KaiC C-terminal tail, we looked into the sequence alignments of KaiAs and KaiCs, and noticed that there were some highly correlated residue changes. For example, when KaiA-259 (numbered according to S. e. PCC 7942) changed from small hydrophobic residues (L, for example) in one sequence to large polar residues (Q, for example) in another sequence, KaiA-257 changed from small alky hydrophobic residues (I, for example) to a bulky hydrophobic residue (F, for example), and KaiC-495 changed from small residues (T, for example) to a bulky residue (H, for example). Based on the NMR structure of KaiA C-terminal domains and KaiC C-terminus derived peptide, we noticed that there were three possibly independent “co-varying residue clusters” between them (Fig. 1C and Table 1). The identified residues had direct interactions (primarily sidechain-sidechain interaction) in a same cluster, but not between two different clusters. In these identified residues, some were previously proved to be critical for the normal oscillation of the KaiABC oscillator. For example, KaiA-A245D decreased the amplitude of the oscillation *in vivo*^22^, KaiA-R249H extended the period *in vivo*^23^, and KaiC-T495A caused arrhythmic oscillation *in vivo*^23^.

To quantitatively analyze the co-variance of the residues in these three clusters, we prepared a phylogenetic tree by concatenating the aligned sequences of KaiA, KaiB, and KaiC in MEGA v5.05 (Supplementary Fig. 1D). From this phylogenetic tree, the residue relative evolution rates (RERs) were calculated (Table 1, Fig. 1D,E, Supplementary Fig. 1E,F). The evolution rates of the residues in these three clusters were quite low (<= 1.0 RER), which indicated that these residues were all quite conserved. To see if the changes of the residues in a same cluster were inter-correlated, we calculated their residue-residue pair positional correlations according to sidechain volume, polarity, or hydrophobicity of the residues (95% sequence coverage, and 95% significant level). As shown in Fig. 1F, the results showed that in both Cluster I and Cluster III, the residue-residue correlated changes were obvious (either positive correlation or negative correlation). In Cluster II, the correlations were much weaker except that the correlated change between KaiA-259 and KaiC-499 was significant.

### Mutagenesis study of the identified co-varying residues

**Figure 2:**
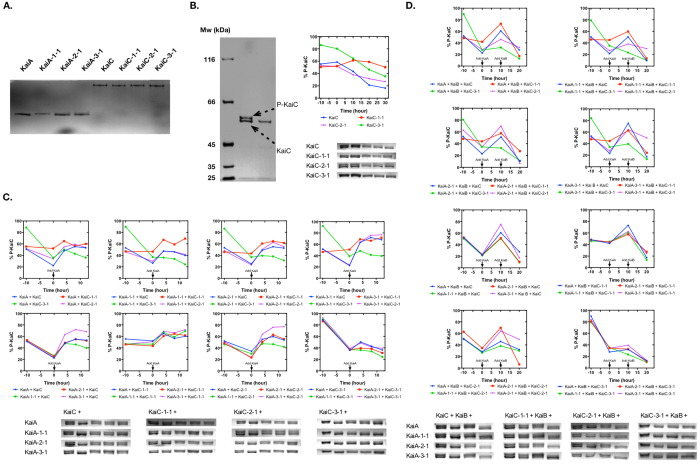
The mutagenesis studies of KaiA and KaiC. (**A**) The analysis of the homogeneities of KaiA and KaiC mutants with native gel. The gel result suggested that the mutations did not disrupt the oligomerization statues of the proteins. (**B**) The stabilities and auto-dephosphorylation of KaiC variants were analyzed by incubation in the assay buffer alone. Left: The SDS-PAGE gel was optimized to be able to differentiate the phosphorylated KaiC (P-KaiC, shown is an example of the wild-type KaiC) from its de-phosphorylated form. KaiC was purified and incubated at 30 °C for 20 h (before the incubation: the 2^nd^ lane; after the incubation: the 3^rd^ lane). Right: KaiCs were incubated alone at 4 °C for 10 h (−10 to 0 h) and then at 30 °C for 30 h (0 to 30 h). ImageJ^58^ was used to analyze the gels and calculate the percentages of phosphorylated KaiCs (P-KaiC%). (**C**) The stimulated phosphorylation of KaiCs by KaiAs. KaiCs were incubated alone at 30 °C for 10 h and then KaiAs were added. (**D**) The de-phosphorylation of KaiCs when KaiAs and KaiB exist. KaiCs were incubated alone at 30 °C for 10 h, followed by the addition of KaiAs (at 0 h) and KaiB at 10 h.

To compensate the interface mutations disruptive to protein-protein interactions, a “second-site suppressor” strategy was adopted by nature and, recently, in rational computational protein design and systems biology^24^,^25^. Briefly speaking, when an interface residue of one protein partner has mutation, the interacting residue in the other protein partner can gain correlated mutations to keep the binding affinity unchanged. To experimentally test if this strategy exists among the residues in the identified clusters between KaiA and KaiC, we introduced mutations to KaiA and KaiC of S. e. PCC 7942 according to the sequence alignment results (Table 1). The mutation KaiA-R249K was not applied since these two residues are very similar. After mutation, the KaiA mutants and the KaiC mutants had same oligomerization states as the wild-type proteins (Fig. 2A), which is a prerequisite for their proper functions.

To check how the mutations affected the stability and auto-phosphatase activity of KaiC, the purified KaiC proteins were incubated alone in the assay buffer at 4 °C for 10 h and then at 30 °C for 30 h^26^. The KaiC mutants showed good stability similar to the wild-type KaiC (Fig. 2B). KaiC-2-1 and KaiC-3-1 showed similar auto-phosphatase activity as the wild-type KaiC, since their dephosphorylation rates were similar. But KaiC-1-1 could not de-phosphorylate very well and no obvious dephosphorylation was detected in 30 h at 30 °C. KaiC-3-1 had a higher initial phosphorylation level and a faster de-phosphorylation rate, which could be caused by the introduced mutations as well.

Next, KaiA proteins were added to test if KaiCs still had the auto-kinase activity, and if KaiAs were still able to stimulate this auto-kinase activity (Fig. 2C). When KaiC was at low phosphorylation levels after being incubated alone at 30 °C for 10 h, all KaiAs could stimulate the phosphorylation of the wild-type KaiC. Compared to the wild-type KaiA, KaiA-3-1 seemed slightly stronger regarding the potential to stimulate KaiC phosphorylation, and this was also held true on the other KaiC mutants. The wild-type KaiA showed good stimulations on the wild-type KaiC and KaiC-2-1. Although KaiC-1-1 showed less de-phosphorylation activity alone, its phosphorylation level stimulated by KaiAs was still similar as the wild-type KaiC, and it was stimulated by all KaiAs as well. KaiC-2-1 showed similar activities as the wild-type KaiC in both the auto-dephosphorylation activity and KaiA-stimulated phosphorylation, but KaiA-2-1 did not do significant benefits to KaiC-2-1. KaiA-2-1 was able to stimulate the phosphorylation of all KaiCs except KaiC-3-1. The phosphorylation of KaiC-3-1 could hardly be stimulated by any KaiAs, although the wild-type KaiA and KaiA-3-1 showed slight activities, which indicated that the mutations probably disrupted its normal interaction with KaiA, or its auto-kinase activity. Together, these data showed that the mutations in KaiA-1-1, KaiA-2-1, KaiA-3-1, KaiC-1-1, and KaiC-2-1 did not significantly compromise KaiA’s function on stimulating the phosphorylation of KaiC, whereas the mutations in KaiC-3-1 largely compromised its KaiA-stimulated phosphorylation, which could not be fully compensated by KaiA-3-1.

Last, we examined the wild-type KaiB’s role on antagonizing KaiA and inducing the dephosphorylation of KaiC (Fig. 2D). KaiB could antagonize all KaiA’s function and switch the wild-type KaiC from the phosphorylation phase to the de-phosphorylation phase. By comparing the slopes of the KaiC de-phosphorylation phases without KaiB (−10 h to 0 h) and with KaiB (10 h to 20 h), it showed that KaiB did not accelerate the de-phosphorylation rate of the wild-type KaiC, which agreed with previous reports^27^,^28^. However, the de-phosphorylation of KaiC-1-1 was faster with KaiB than without KaiB, which, surprisingly, showed that KaiB likely changed the de-phosphorylation rates of KaiC-1-1 when KaiA existed. But KaiA-1-1 did not act differently from the other KaiA variants when mixed with KaiB and KaiC-1-1. KaiC-2-1 had slower de-phosphorylation rates than the wild-type KaiC when mixed with KaiB and all KaiA variants except KaiA-2-1, with which, KaiC-2-1 de-phosphorylated much faster. Nonetheless, KaiA-2-1 did not significantly affect the de-phosphorylation rate of the wild-type KaiC. KaiC-3-1 showed slightly faster de-phosphorylation when KaiB was added than with KaiAs alone, whereas KaiA-3-1 acted normally just as the wild-type KaiA.

The above data showed that the interaction between KaiA and KaiC is more complicated than shown in the NMR complex structure of the KaiA C-terminal domains and the KaiC C-terminus derived peptide^19^. None of the KaiA mutants (KaiA-1-1, KaiA-2-1, or KaiA-3-1) lost its function on stimulating KaiC phosphorylation, although KaiA-3-1 showed slightly higher activities on all KaiC variants. Similarly, in the KaiC mutants, neither KaiC-1-1 nor KaiC-2-1 lost its phosphorylation potential stimulated by KaiA. KaiC-3-1 could not get phosphorylated well no matter if KaiAs existed, but when KaiAs existed, its de-phosphorylation rates were slower, so the mutations should not have totally destroyed its interaction with KaiA. However, the different behaviors between the wild-type protein (KaiA or KaiC) and its mutants indicated that the mutations indeed affect the interaction between KaiA and KaiC. Not as expected, the mutant pairs in a same co-varying residue cluster did not show significant compensation. The activities shown by the different combinations of cross-cluster mutants (such as KaiA-1-1 and KaiC-2-1) further indicated that even simultaneous mutations in both KaiA and KaiC would not totally break their interaction.

### Molecular dynamics (MD) simulation of KaiA C-terminal domains

**Figure 3:**
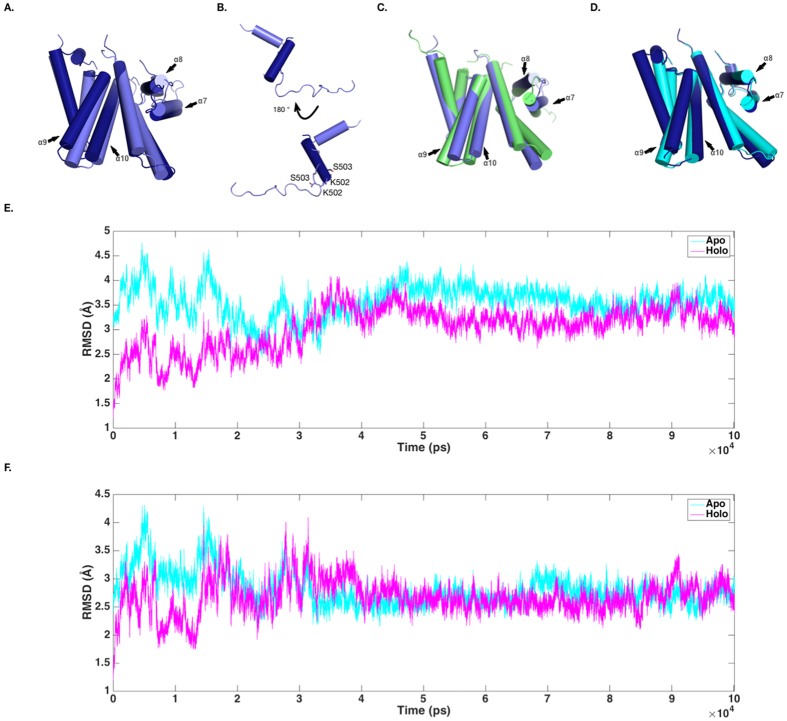
The conformational analyses of KaiA. (**A**) The conformational difference of the KaiA C-terminal domains in the X-ray structure (PDB ID: 5C5E, in dark blue) and in the NMR structure (PDB ID: 1SV1). (**B**) The conformational difference of the KaiC C-terminus derived peptides in the X-ray structure (PDB ID: 5C5E, in dark blue) and in the NMR structure (PDB ID: 1SV1, in light blue). For clarity, the residues shown in sticks are marked according to the residues in the KaiC C-terminal tail of S. e. PCC 7942. (**C**) The conformational difference between the apo (PDB ID: 1Q6B, in green) and the holo (PDB ID: 1SV1, in light blue) KaiA C-terminal domains of T. e. BP-1. (**D**) The conformational differences between the apo (PDB ID: 1R8J, in cyan) and the holo (PDB ID: 5C5E, in dark blue) KaiA C-terminal domains of S. e. PCC 7942. (**E**) The molecular dynamics simulation of the structure of KaiA C-terminal domains (PDB ID: 1SV1). The KaiC C-terminus derived peptide was not included in the simulation. The backbone RMSD differences were calculated for the whole KaiA C-terminal domain compared to the apo structure (1Q6B) and the holo structure (1SV1) respectively. (**F**) The RMSD differences of the helices 9 and 10 of KaiA C-terminal domains in the MD simulation.

During our study, Pattanayek and Egli reported an X-ray complex structure of a KaiC C-terminus derived peptide and the full-length KaiA homo-dimer of S. e. PCC 7942^21^ (Fig. 3A,B). Surprisingly, the peptide adopted an α-helix conformation, which is totally different from the previous NMR structure, in which the KaiC C-terminal peptide was a random coil^19^ (Fig. 3B). Since there were several differences between these two studies, including the technology (NMR vs. X-ray), the protein origins (*T. elongatus* BP-1 vs. *S. elongatus* PCC 7942), and the length of proteins (the C-terminal domain of KaiA, a KaiC C-terminus derived 35-mer peptide vs. the full-length KaiA, a KaiC C-terminus 20-mer peptide), it remains unclear which one is the correct model for KaiA-KaiC interaction.

The mutation sites in our study were predicted according to the NMR complex structure, but the mutagenesis study did not support the importance of those interaction residues and the compensation of the interface residues very well. In the X-ray structure, the peptide was too short, and only two mutated sites (K502 and S503) in our study could be seen, but they seemed not critical to the inter-chain interaction as mentioned in^21^ (Fig. 3B). Therefore, the X-ray complex structure could not be fully supported by our mutagenesis data as well.

It was pointed out that the C-terminal domain of KaiA had a conformational difference in the helices 9/10 before and after binding KaiC, and this conformational change was likely induced by the binding of KaiC^19^ (Fig. 3A). This conformational difference also exists between the X-ray structures of KaiA C-terminal domains of S. e. PCC 7942 (Fig. 3C). Between the X-ray structure (PDB ID: 5C5E) and the NMR structure (PDB ID: 1SUY), the differences in this region are also obvious (Fig. 3D). Therefore, we supposed that the structural difference of KaiA C-terminal domains before and after binding KaiC C-terminus derived peptide was probably due to a more complicated process, which combines conformational selection and followed induced fit and stabilization. To verify this possibility, we did molecular dynamics simulation using the structures of KaiA with (holo, PDB ID: 1SV1) and without (apo, PDB ID: 1Q6B) the binding of KaiC C-terminus derived peptides (Fig. 3E,F). As represented by the crossed trajectories (the RMSD values were calculated against the indicated experimental structures instead of the equilibrated MD conformations), the KaiA C-terminal domain was getting closer to the apo conformation (decreasing RMSD values compared to the apo conformation) while it was moving away from the holo conformation (increasing RMSD values compared to the holo conformation), and *vise verse* (Fig. 3E). Most importantly, the helices 9/10 in KaiA C-terminal domain had the similar conformational changes as well (Fig. 3F). Therefore, the MD simulations suggested that, in agreement with the experimental evidence, the helices 9/10 of KaiA were very flexible, and quickly oscillated between different statuses, including (but not limited to) the conformations previously revealed in the NMR models and the X-ray structures (Movies S1). In addition, this conformational oscillation seemed to be independent of the starting structure (Supplementary Fig. 2, and Movie S2). Comparing the different conformations, we preferred the hypothesis that the KaiA conformations in the NMR structure and in the X-ray structure were two different transient statuses, and therefore they could accommodate and needed different conformations of the KaiC C-terminal derived peptide. The NMR conformation could be close to the “tethered” model of KaiA-KaiC interaction, and the X-ray conformation could be close to the “engaged” model^18^. This hypothesis was supported by the fitting trials of the NMR structure and the X-ray structure with the full-length KaiC hexamer structure as presented in^21^.

### Mathematic modeling of the KaiABC oscillator

If the hypothesis above was valid, we could represent the binding of KaiA-KaiC with the following steps,


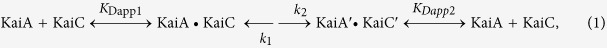


in which, KaiA′ and KaiC′ represent the statuses after conformational changes. When the binding constant between KaiA and KaiC is measured in experiment, it is hard to differentiate these two complex statuses (KaiA • KaiC and KaiA′ • KaiC′), so the apparent binding (or dissociation) constant would be from their combined contribution (denoted as *K*_Dfit_). As described in the Supplementary Information, the relationship between apparent dissociation constants (*K*_Dapp1_, *K*_Dapp2_) and the combined constant (*K*_Dfit_) for the binding of KaiA and KaiC would be


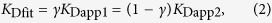


where there is a number γ such that 0 < γ < 1. Obviously, *K*_Dfit_ is smaller than the possible apparent dissociation constant of either binding step. That says, if our hypothesis is true but the binding of KaiA and KaiC is to be modeled as a one-step process for simplicity in the mathematic modeling of the KaiABC osciallator, the experimental *K*_D_ value of KaiA-KaiC interaction could be too high to be used if the whole binding process was not fully detected.

**Figure 4:**
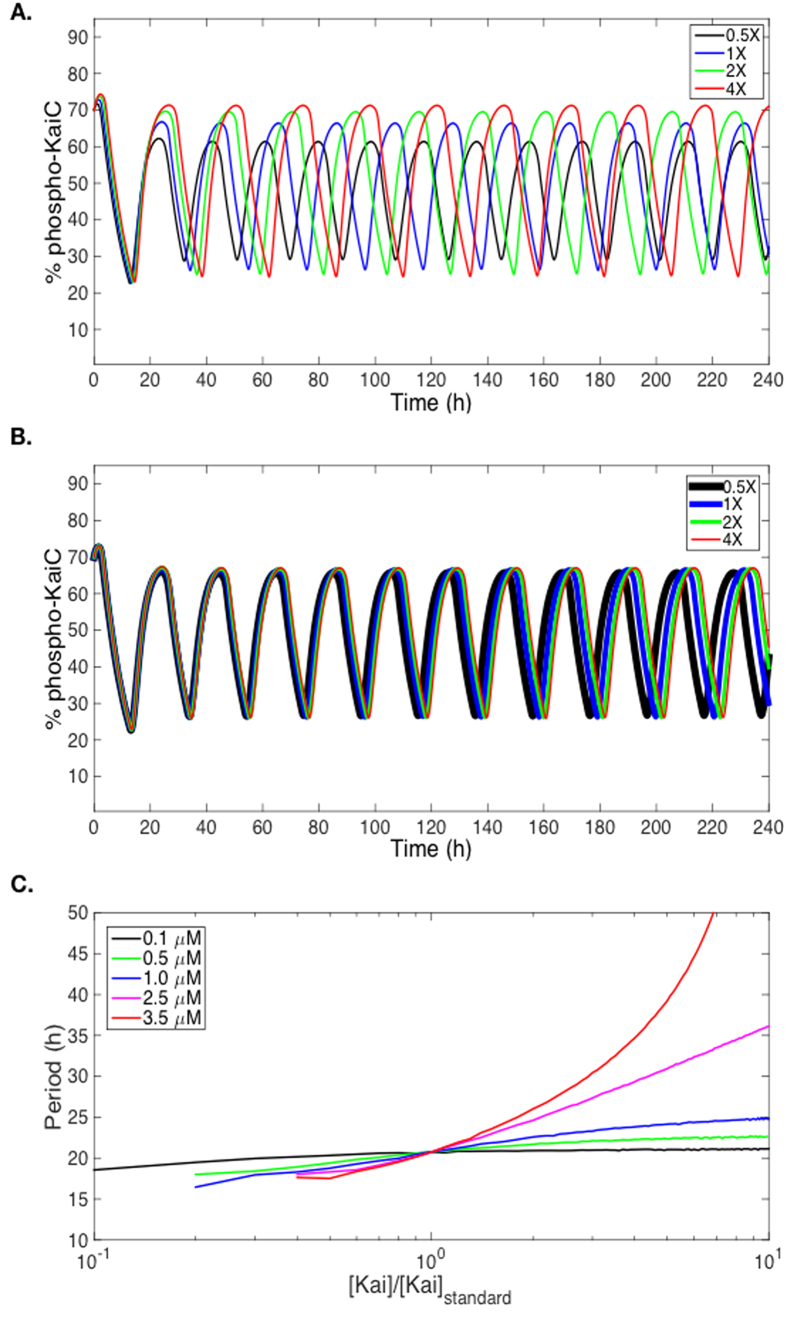
The mathematic modeling of the oscillation of the KaiABC oscillator. The periods in the modeling is ~21 h instead of 24 h. (**A**) The equilibrium constant (*K*_D_) of KaiA-KaiC interaction was set to 1 μM as Rust *et al.*^28^. The period changed obviously when the total protein concentration varied (0.5X, 1X, 2X, 4X). The 1X concentration represents the standard protein concentrations (KaiA: 1.3 μM; KaiB: 3.4 μM; KaiC: 3.4 μM) used in the *in vitro* reconstitution experiment. (**B**) When the equilibrium constant of KaiA-KaiC interaction was set to 0.1 μM according to our hypothesis, the period of the oscillation was more robust against the variation of the total protein concentration. (**C**) The period of the oscillation (y-axis) is more robust against the variation of protein concentration (x-axis) when *K*_D_ is smaller.

Rust *et al.*^28^ presented a nice mathematic model for the KaiABC oscillator previously. In their model, they used a *K*_D_ value (1.0 μM) smaller than observed (2.5 μM) in experiment^29^ when considering the binding equilibrium of KaiA and KaiC. The reason, as they discussed, was that the model required a small *K*_D_ to generate the oscillation matched to the experimental result. As studied by Kageyama *et al.*^29^ and discussed by Phong *et al.*^30^, the KaiABC oscillator should be robust against variations in total protein concentrations. Instead, the model of Rust *et al.* generated oscillations with varied periods when the total protein concentration was changed (the ratios between clock proteins were fixed) (Fig. 4A), which did not match the experimental results perfectly^29^,^30^. Because Kageyama *et al.*^29^ showed that KaiA and KaiC could form more stable complexes when the incubation time was extended, we supposed that the *K*_D_ value measured in their study was mainly from the first binding step in (1). Based on the model of Rust *et al.* and our hypothesis, we chose to set the *K*_D_ value to be 0.1 μM, which was estimated from the dissociation rates under different incubation times for KaiA and KaiC in^29^. In that report^29^, when the interacting time between KaiA and KaiC was extended from 3 min to 60 min, the complex half time (t_1/2_) increased about 20 folds. So we simply estimated the *K*_D_ value to be about 20x lower than the reported value since t_1/2_ = ln(2)/*k*_d_. Then we were able to perfectly model the robustness of the oscillation under varied total protein concentrations (Fig. 4B). The model also showed that the circadian oscillation was more robust against protein concentration variations with smaller *K*_D_ values (Fig. 4C). Therefore, the mathematic modeling agreed with our analysis on the *K*_D_ value of KaiA-KaiC interaction, which then supported our hypothesis on the dynamic mechanism of KaiA-KaiC interaction.

## Discussion

The KaiABC system is the core circadian oscillator of cyanobacteria, and consists of three proteins, KaiA, KaiB, and KaiC. This system was the first validated non-TTFL circadian oscillator, and could be reconstituted *in vitro*^12^,^13^,^31^. KaiC is able to oscillate between the un-phosphorylated form and the phosphorylated forms, which then generates oscillated output signals via the interaction with downstream proteins. KaiA’s function is to stimulate the phosphorylation of KaiC, whereas KaiB’s role is to induce KaiC’s de-phosphorylation by antagonize KaiA’s function^11^,^32^,^33^. Although the KaiABC system has very simple elements and a lot of research has been done on it, the detailed molecular mechanisms for their interactions are still elusive, largely due to the unknown complex structures and interaction mechanisms of the hetero-complexes between these three clock proteins^15^,^31^.

To understand the interaction mechanism between KaiA and KaiC, Vakonakis *et al.*^19^ acquired a complex structure of the KaiA C-terminal domain and a KaiC C-terminus derived peptide by NMR, and recently, Pattanayek and Egli^21^ solved a complex structure of the full-length KaiA and a shorter KaiC C-terminus derived peptide. However, their structures were contradicted with each other (Fig. 3A,B), and it is not clear which one would be correct. In stead, Ishii *et al.*^34^ adopted the site-directed spin labeling-ESR method to map the interacting residues between KaiA and KaiC, and largely supported the NMR model, but the labeling of mutant proteins could introduce artificial interactions.

In this study, we set out to use the “secondary-site suppressor” strategy to understand the interaction details between KaiA and KaiC. We used evolution analyses to identify the critical residues and the possible mutations, based upon the NMR structure of the complex between KaiA C-terminal domains and a KaiC C-terminus derived peptide. However, our experimental data did not support the NMR structure very well, nor the recently published X-ray structure of KaiA and another shorter KaiC C-terminus derived peptide. Interestingly, the peptide conformations were totally different from each other in those experimental complex structures. It was suggested that the NMR structure might be artificial^21^, but by combining all of these data, we proposed that the interaction between KaiA and KaiC might be a dynamic process with different transient interactions including those two (NMR and X-ray) conformations. It was known that KaiA and KaiC could have different binding conformations^18^; therefore, it is possible that the NMR conformation and the X-ray conformation captured different binding statuses. In this way, our mutagenesis study, as well as the difference in these two experimental complex structures, could be explained. Our MD simulations and mathematical modeling of the oscillation supported our hypothesis. In the MD simulation, the conformation of the apo KaiA oscillated between the apo conformation and the holo conformations, so it is likely that KaiC could be captured more easily and allowed to make further conformational changes after being captured. As a further support, the mathematical modeling inferred from the two-step binding hypothesis had a better fitting with the experimental results.

Why could our hypothesis be possible? The KaiC C-terminal tail is an intrinsically disordered region (IDR), and its known function is to bind KaiA. Similar with intrinsically disordered proteins (IDPs), IDRs could adopt different conformations, especially when bind to protein partners^35^,^36^. Meanwhile, the protein complexes formed by IDRs are often very flexible and could only be described as structural ensembles^36^. Therefore, it is reasonable that the KaiC C-terminal tail could have different conformations upon binding to KaiA. The extended conformation of the KaiC C-terminal tail (as in the NMR structure) could be doing “protein fishing”^37^,^38^, and the followed folded conformation (as in the X-ray structure) could be really functioning. During this binding process, the KaiC C-terminal tail has conformational change (induced fit) after conformational selection^39^. This possibility is also supported by the finding that a more stable KaiA-KaiC complex would form after the initial association^29^. In this way, the interaction mediated by this IDR could be finely regulated, and has high specificity^40^.

Another interesting but pending for further investigation finding in our study was about the role of KaiB. KaiB was supposed to only antagonize KaiA’s function but not affect the de-phosphorylation of KaiC^27^,^28^. However, our data showed that although the wild-type KaiB did not change the de-phosphorylation rate of the wild-type KaiC (when KaiA existed), it seemed to be more complicated when mixed with KaiA mutants and KaiC mutants. This finding might indicate that KaiB’s function is not as simple as previously supposed, which could be an important reason that the binding site of KaiB on KaiC is still under hot debates^26^,^41^,^42^,^43^,^44^,^45^,^46^,^47^. A further exploring in this direction might be very helpful for understanding the complicated interactions between these three proteins.

## Methods

### Protein sequence alignments and analyses

The protein sequences were obtained from the protein sequence database of NCBI (http://www.ncbi.nlm.nih.gov). Only the species having all protein sequences available for KaiA, KaiB, and KaiC were considered. KaiA1, KaiB1, or KaiC1 was considered when there were more than one copy of these proteins. The sequences were aligned in MEGA v5.05^20^ using the ClustalW protocol and the default weight matrix Gonnet. Finally, the alignments were manually modified to keep the intact of secondary structures.

The phylogenetic tree was generated with the concatenated aligned sequences of these three proteins prepared from the respective protein sequence alignment in MEGA v5.05. The alternative topologies of the trees were firstly generated with the neighbor-joining algorithm and JTT model, and then evaluated with ML method and WAG model to find the best topology and estimate branch lengths. Then the evaluated tree was used to calculate position-by-position evolution rates with WAG model.

A Phylip NJ tree was generated in ClustalX v2.1^48^ using the concatenated sequence alignment of KaiABC. Then the Phylip NJ tree was used to calculate pair positional correlations in CRASP^49^ according to residue characteristics. The type of matrix was linear correlation, the variability threshold was 2, the significant level was 95%, and the gap number threshold was 5%. The amino acids were analyzed by volume^50^, polarity^51^, and hydrophobicity^52^.

### Protein expression and purification

The plasmids for expressing the wild-type KaiA, KaiB, and KaiC proteins of S. e. PCC 7942 were kindly provided by Dr. Carl Johnson (Vanderbilt University). The DNA sequences were inserted in pGEX-6P-1^53^, and the proteins were expressed as GST-tagged products in BL21(DE3)^54^. Site-directed mutations were introduced into the corresponding coding sequences with overlap extension PCR. The GST-tagged proteins were purified with GST-affinity beads, followed by the cleavage of GST with PreScission Protease and the further purification with size-exclusion columns. The plasmid containing the coding sequence of PreScission Protease was a kind gift from Dr. Luhua Lai (Peking University) and purified with the similar procedures.

### *In vitro* study of the functions of KaiA, KaiB, and KaiC

The proteins were analyzed with 8–12% SDS-PAGE for purity and molecular masses. The oligomerization statuses of the proteins were analyzed with 8% native gels. Protein concentrations were determined with the Bradford assay. The assay buffer was 50 mM Tris-HCl (pH 8.0), 150 mM NaCl, 5 mM ATP, 5 mM MgCl_2_, and 0.01% Tween-20. To check the auto-dephosphorylation of KaiC, KaiC was incubated alone in the assay buffer at 4 °C. To test KaiA’s function on stimulating KaiC’s phosphorylation, KaiA was added to de-phosphorylated KaiC at 30 °C. To examine KaiB’s antagonizing function on KaiA, KaiB was added to the mixture of KaiA and KaiC after KaiC was stimulated by KaiA for 10 h at 30 °C. The refernce ratio of KaiA:KaiB:KaiC in the reaction system was 1:1:2 (m/V) as previous^55^. The proteins were collected at indicated time points and analyzed with 8% SDS-PAGE.

### Molecular dynamics simulation

The initial protein structures were obtained from PDB (http://www.pdb.org), and used for molecular dynamics simulation in NAMD^56^. For NMR models, the lowest energy model was used as the representative structure. Periodic water boxes were added to wrap the protein with 10 Å of boundary distances, and the production runs were performed after the systems were equilibrated well. Na^2+^ and Cl^−^ were added to 0.15 mol/L and counteracted the net charger of the system. The Charmm parameters from c35b2_c36a2 were used, and the smooth particle-mesh Ewald (PME) method was enabled. To do the temperature and pressure equilibrium, the system temperature was increased from 0 K to 300 K with a 20 K step in the Berendsen step, followed by 1 ns of equilibrium at 310 K. The atom coordinates were recorded per picosecond (ps) throughout the simulations.

### Mathematical modeling

The mathematical modeling of the oscillation was based on the model presented by Rust *et al.*^28^. The model considering the binding equilibrium of KaiA and KaiC was used, and the dissociation constant was varied for modeling in this study.

## Additional Information

**How to cite this article**: Dong, P. *et al.* A dynamic interaction process between KaiA and KaiC is critical to the cyanobacterial circadian oscillator. *Sci. Rep.* **6**, 25129; doi: 10.1038/srep25129 (2016).

## Supplementary Material

Supplementary Video S1

Supplementary Video S2

Supplementary Information

## Figures and Tables

**Table 1:** The residues identified and mutated in this paper.

Cluster	Name	Residue	Wild-type	Mutation	RER
I	KaiA-1-1	239	E	N	0.589
		245	A	W	0.587
		249	R	–	–
	KaiC-1-1	502	K	R	0.086
II	KaiA-2-1	259	L	Q	0.430
	KaiC-2-1	497	I	V	0.365
		499	V	T	1.023
		503	S	T	1.053
III	KaiA-3-1	253	R	H	0.165
		254	S	K	0.370
		257	I	F	0.691
	KaiC-3-1	493	S	V	0.300
		495	T	H	0.399

The Relative Evolution Rates (RERs) were calculated in MEGA v5.05.
