# Supplementary material for: A dynamic interaction process between KaiA and KaiC is critical to the cyanobacterial circadian oscillator
Source: Sci Rep. 2016 Apr 26;6:25129. doi: 10.1038/srep25129 (PMC4844972; doi:10.1038/srep25129)
Supplement: Supplementary Information [file srep25129-s3.pdf]

A dynamic interaction process between KaiA and KaiC is critical to the cyanobacterial circadian oscillator

Pei Dong[1,2], Ying Fan[2], Jianqiang Sun[3], Mengting Lv[2], Ming Yi[4], Xiao Tan[1,2], Sen Liu[1,2,*]

[1]Hubei Key Laboratory of Tumor Microenvironment and Immunotherapy, China Three Gorges University, Yichang 443002, China

[2]College of Medical Science, China Three Gorges University, Yichang 443002, China

[3]School of Statistics, Shandong Institute of Business and Technology, Yantai, 264005, China

[4]Department of Physics, College of Sciences, Huazhong Agricultural University, Wuhan 430070, China

[*]Corresponding author: senliu.ctgu@gmail.com

Supplementary Text S1. The analysis of the KaiA-KaiC binding equilibrium

In this section we will prove that K_{Dfit}, which is the apparent value of the real equilibrium constants of two steps of the KaiA-KaiC binding processes, should be smaller than the apparent values corresponding to K_{Dapp1} and K_{Dapp2} in the following part. For simplicity, A stands for KaiA, and C stands for KaiC (C1 and C2 are two different conformational statuses).

$$\mathrm{A} + \mathrm{C} \underset{kd_1}{\overset{ka_1}{\rightleftharpoons}} \mathrm{AC}_1 \underset{k_1}{\overset{k_2}{\rightleftharpoons}} \mathrm{AC}_2 \underset{ka_2}{\overset{kd_2}{\rightleftharpoons}} \mathrm{A} + \mathrm{C}$$

According to the binding processes shown above and the mass action principle, we obtain that:

$$\frac{d[AC_1]}{dt} = ka_1[A][C] - kd_1[AC_1] + k_1[AC_2] - k_2[AC_1] \qquad (1)$$

$$\frac{d[AC_2]}{dt} = ka_2[A][C] - kd_2[AC_1] + k_2[AC_1] - k_1[AC_2] \qquad (2)$$

Moreover, we suppose that all steps above are rapid and pre-equilibrium processes compared to the phosphorylation processes. Thus, we have

$$\frac{d[AC_1]}{dt} = ka_1[A][C] - kd_1[AC_1] + k_1[AC_2] - k_2[AC_1] = 0 \qquad (3)$$

$$\frac{d[AC_2]}{dt} = ka_2[A][C] - kd_2[AC_2] + k_2[AC_1] - k_1[AC_2] = 0 \qquad (4)$$

Combining (3) with (4), we may conclude that

$$(ka_1 + ka_2)[A][C] = kd_1[AC_1] + kd_2[AC_2] \qquad (5)$$

Suppose that $[AC_{\mathrm{tot}}]$ is the total concentration of complex AC, i.e.

$$[AC_{\mathrm{tot}}] = [AC_1] + [AC_2] \qquad (6)$$

Thus, there is a number γ $(0 < \gamma < 1)$ such that

$$[AC_1] = \gamma[AC_{\mathrm{tot}}] \qquad (7)$$

Combining (5), (6), and (7), we have

$$(ka_1 + ka_2)[A][C] = (\gamma kd_1 + (1-\gamma)kd_2)[AC_{\mathrm{tot}}] \qquad (8)$$

$$(ka_1 + ka_2)[A][C] = \left(kd_1 + \frac{(1-\gamma)}{\gamma}kd_2\right)[AC_1] \qquad (9)$$

$$(ka_1 + ka_2)[A][C] = \left(\frac{\gamma}{(1-\gamma)}kd_1 + kd_2\right)[AC_2] \qquad (10)$$

Denoting $K_{\mathrm{Dapp1}} = [A][C]/[AC_1]$, $K_{\mathrm{Dapp2}} = [A][C]/[AC_2]$, and from (8), (9), (10), we obtain

$$K_{\mathrm{Dfit}} = \frac{[A][C]}{[AC_{\mathrm{tot}}]}$$

$$= \frac{(\gamma kd_1 + (1-\gamma)kd_2)}{ka_1 + ka_2}\ , \qquad (11)$$

$$K_{Dobs1} = \frac{[A][C]}{[AC_1]}$$

$$= \frac{(\gamma kd_1 + (1-\gamma)kd_2)}{\gamma(ka_1 + ka_2)} \ , \qquad (12)$$

$$K_{Dobs2} = \frac{[A][C]}{[AC_2]}$$

$$= \frac{(\gamma kd_1 + (1-\gamma)kd_2)}{(1-\gamma)(ka_1 + ka_2)} \ , \qquad (13)$$

According to (11), (12) and (13), it is obvious that

$$K_{\mathrm{Dfit}} = \gamma \, K_{\mathrm{Dapp1}} = (1-\gamma) \, K_{\mathrm{Dapp2}} \qquad (14)$$

Supplementary Figure S1. The sequence analyses of the clock proteins. (A) The sequence alignment of 65 KaiA sequences aligned in MEGA v5.05. The alignments were rendered with STRAP. The secondary structure elements were shown according to the protein structure of Synechococcus elongates PCC 7942. (B) The sequence alignment of 65 KaiB sequences aligned in MEGA v5.05. (C) The sequence alignment of 65 KaiC sequences aligned in MEGA v5.05. (D) The phylogenetic tree generated from concatenated KaiA/KaiB/KaiC sequences. (E) The calculated Relative Evolution Rates (RERs) of KaiA residues. (F) The calculated RERs of KaiC residues.

(A)

```
                                                                           β1      α1
                                                                                10
Synechococcus e.  PCC 7942              1  ......................................MLSQIAICIWVESTAIL
Acaryochloris m.  MBIC11017             1  .............................MTLSQTAVSRPQIFICTLLSSESLVD
Acaryochloris sp.  CCMEE 5410          1  .............................MTLSQTAVSRPQIFICTLLSSESLVD
Anabaena v.  ATCC 29413                1  .......................................................
Arthrospira m.  CS-328                 1  ..............................................MFIPSIELS
Arthrospira m.  FACHB-438              1  ..............................................MFIPSIELS
Arthrospira p.  str.  Paraca          1  ..............................................MFIPSIELS
Crocosphaera w.  WH 0003               1  .......................................................
Crocosphaera w.  WH 8501               1  ..............................MCLSIHRPLQTRLSICLCSSDKPFT
Cyanobium sp.  PCC 7001                1  .....................................MPESVLTVASLVHDPALQ
Cyanothece sp.  ATCC 51142             1  ......................................MQTRLSICLYSSDEHLT
Cyanothece sp.  CCY0110                1  .......................................................
Cyanothece sp.  PCC 7424               1  ............................MHFRLTIYLFIPDPS..........LA
Cyanothece sp.  PCC 7425               1  ...................................MSTHHQLSICGLVSSEAVAQ
Cyanothece sp.  PCC 7822               1  ............................MHFRLTIYLFVPETS..........IA
Cyanothece sp.  PCC 8801               1  ......................................MSSRLSICLYISDRTIA
Cyanothece sp.  PCC 8802               1  ......................................MSSRLSICLYISDRTIA
Cylindrospermopsis r.  CS-505          1  .......................................................
Fischerella sp.  JSC-11                1  .......................................................
Leptolyngbya b.  IAM M-101             1  .....................................MRPQLSIGVYLNSPALPQ
Lyngbya sp.  PCC 8106                  1  .......................................................
Microcoleus c.  PCC 7420               1  .....................................MHPQLSIGIFVRSPALAQ
Microcoleus v.  FGP-2                  1  ......................................MLPKLSIGIFVRSEEIA
Microcystis a.  NIES-843               1  ...............................................MSSPPGLV
Microcystis a.  PCC 7806               1  ................................MTIYAFVPTDSPLTGVSSPLGLV
Microcystis a.  PCC 7820               1  ...............................................MSSPLGLV
Microcystis a.  PCC 7941               1  .MAGGCLLNRESLTYCLSISIYLAGEKTLPPRLTIYAFVPTDSPLTGVSSPLGLV
Microcystis a.  PCC 9443               1  ............................MPPRLTIYAFVPTDSPLTGVSSPLGLV
Microcystis a.  PCC 9701               1  ............................MPPRLTIYAFVPTDPPLTGVSSPLGLV
Microcystis a.  PCC 9717               1  ............................MPPRLTIYAFVPTDPPLTGVSSPPGLV
Microcystis a.  PCC 9806               1  MAGGGCLLNRESLTYCLSISIYLAGEKTLPPRLTIYAFVPTDSPLTGASSPLGLV
Microcystis a.  PCC 9807               1  ............................MPPRLTIYAFVPTDSPLTGVSSPLGLV
Microcystis sp.  T1-4                  1  ............................MPPRLTIYAFVPTDSPLTGVSSPPGLV
Moorea p.  3L                          1  ....................................MLLSQLTICTLISSDSLAQ
Nodularia s.  CCY9414                  1  .......................................................
Nostoc a.  0708                        1  .......................................................
Nostoc c.                              1  .......................................................
Nostoc p.  PCC 73102                   1  .......................................................
Nostoc sp.  PCC 7120                   1  .......................................................
Nostoc sp.  PCC 9709                   1  .......................................................
Oscillatoria sp.  PCC 6506             1  .............MFAKELPKLTDSALSGNFGIEPNTALRSELSICIFLRSEELA
Raphidiopsis b.  D9                    1  .......................................................
Synechococcus e.  PCC 6301             1  ......................................MLSQIAICIWVESTAIL
Synechococcus sp.  PCC 7335            1  ......................................MPAQFLLGVFVPAASLA
Synechococcus sp.  BL107               1  .....................................MARPALTVALLLSSSALV
Synechococcus sp.  CB0101              1  .....................................MSQPALTIASLLPDPRVE
Synechococcus sp.  CB0205              1  .....................................MTQPALTIASLLSDPRLV
Synechococcus sp.  CC9311              1  .......MACAAIERQTKIFDLRALFRQDSIAHFSFWMTRPALTIAFLLRSPELE
Synechococcus sp.  CC9605              1  .....................................MARPALTVALVLGSPALV
Synechococcus sp.  CC9902              1  .....................................MARPALTVALLLSSSALV
Synechococcus sp.  JA-2-3B             1  ................................MLPDTHPLRINLLLDWEPPAGEP
Synechococcus sp.  JA-3-3Ab            1  .......................................................
Synechococcus sp.  PCC 7002            1  ......................................MVSKLSLYLVTPPGFQP
Synechococcus sp.  RCC307              1  .....................................MAVTELVIGALVRNELLL
Synechococcus sp.  RS9916              1  .....................................MNGPALAIALLLKSPKLL
Synechococcus sp.  RS9917              1  .....................................MTGPALTIALLLRTSPLI
Synechococcus sp.  WH 5701             1  .....................................MTQPALTVASLIRDEQLR
Synechococcus sp.  WH 7803             1  .....................................MARPALTIALLLTSKELV
Synechococcus sp.  WH 7805             1  .....................................MARPALTIALLLTTQELV
Synechococcus sp.  WH 8016             1  .....................................MSRPALTIAFLLRSPELE
Synechococcus sp.  WH 8102             1  .....................................MARPGLTIALLLTTPNLV
Synechococcus sp.  WH 8109             1  .....................................MARPALTVALVLASPALV
Synechocystis sp.  PCC 6803            1  .......................................MQSPLSLCLFAPEHVA
Thermosynechococcus e.  BP-1           1  ...................................MAQSTALTICGLVYSPAIGQ
Trichodesmium e.  IMS101               1  ................MKISRITDSQINPDLPTVP.KTLSTKLFICIYLHDDKFA
consensus
```

logo

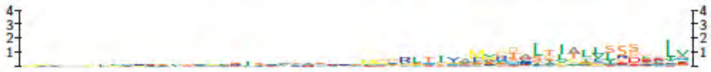

```
                                     α1        β2            α2          β3      α3
                                   20         30           40         50         60
Synechococcus e.  PCC 7942       18  QDCQRALSADRYQLQVCE......SGEMLLEYAQTHRDQIDCLILVAANP...SF
Acaryochloris m.  MBIC11017      27  LYTQILEGDRYSLVHAT.......YDN.FLEIVEQGKHRIDCLIFEKNAA....L
Acaryochloris sp.  CCMEE 5410    27  LYTRILEGDRYSLVHAT.......YDN.FLEIVEQGKHRIDCLIFEKNAA....L
Anabaena v.  ATCC 29413           1  .......................................................
Arthrospira m.  CS-328           10  QSVSSYLPKEHYIIIQLD......SPEEFIEYVTEHKHQLDCLILEWSPQ....W
Arthrospira m.  FACHB-438        10  QSVSSYLPKEHYIIIQPD......SPEEFIEYVTEHKHQLDCLILEWSPQ....W
Arthrospira p.  str.  Paraca     10  QSVSSYLPKEHYIIIQPD......SPEEFIEYVTEHKHQLDCLILEWSPQ....W
Crocosphaera w.  WH 0003          1  ..MANHLSGDRYRLHIID......QVEELIPFLKAHSETIDCLIVREEAS....I
Crocosphaera w.  WH 8501         26  QLLANHLSGDRYRLHIID......QVEELIPFLKAHSETIDCLIVREEAS....I
Cyanobium sp.  PCC 7001          19  AAVARWLAGGRYALVRVD......PSDDPVAALERLREDFDALLIEEGSVD....
Cyanothece sp.  ATCC 51142       18  QLLTHHLSGDRYRLHIID......QLEDLVPFLEDHSETIDCLIVKQEPS....I
Cyanothece sp.  CCY0110           1  ..MTHHLSSDRYRLHIID......QLQDLVPFLKEHSETIDCLIVRQEPS....I
Cyanothece sp.  PCC 7424         18  QSLTHLLSTERYNINLVD......SPNELIDVIEDNKEKIDCLVVLNQPS....L
Cyanothece sp.  PCC 7425         21  TLS.QALDDRFSLVQFQ.......QESDFRQFLEQEKHDIDCLVLESSLD....L
Cyanothece sp.  PCC 7822         18  QSLTRLLSTERYTLIFVN......SMDELLEAIEENKDKIDCLVVLKLPT....L
Cyanothece sp.  PCC 8801         18  QNLAEILADKPYCLKKVD......SPEELLKFVESRSEQIDCLIIFRDTT....V
Cyanothece sp.  PCC 8802         18  QNLAEILADKPYCLKKVD......SPEELLEFVESRSEQIDCLIIFRDTT....V
Cylindrospermopsis r.  CS-505    1  .......................................................
Fischerella sp.  JSC-11           1  .......................................................
Leptolyngbya b.  IAM M-101       19  SLEQILASHERYAMTVVE......STQDFFQLIQQHKQQLDCLILQDSSE....L
Lyngbya sp.  PCC 8106             1  ...MSSLQSDRYAVTQVH......SVEELLQYVSKEREQLDCLIVQVDMD....L
Microcoleus c.  PCC 7420         19  SLSQVLS.GDRYVLSITN......SASEFLEFVEQKQQHIDCLVLQDDDS....L
Microcoleus v.  FGP-2            18  ASAIELLRGERYALTHIK......SAVEFLGFLK.HNYHLDCLVLEVDGD....L
Microcystis a.  NIES-843          9  NSLTALLANERYHLTLGS......SSSELLANIEAQKEKIDCLLVVFHPS....L
Microcystis a.  PCC 7806         24  NSLTALLANERYNLTLGS......SSSELLANIEAEKEKIDCLLVVFHPS....L
Microcystis a.  PCC 7820          9  NSLTALLANERYNLTLGS......SSSELLANIEAEKEKIDCLLVVFHPS....L
Microcystis a.  PCC 7941         55  NSLTALLANERYNLTLGS......SSSELLANIEAEKEKIDCLLVVFHPS....L
Microcystis a.  PCC 9443         28  NSLTALLANERYNLTLGS......SSSELLANIEAEKEKIDCLLVFFHPS....L
Microcystis a.  PCC 9701         28  NSLTALLANERYNLTLGS......SSSELLANIEAQKEKIDCLLVVFHPS....L
Microcystis a.  PCC 9717         28  NSLTALLANERYHLTLGS......SSSELLANIEAQKEKIDCLLVVFHPS....L
Microcystis a.  PCC 9806         56  NSLTALLANERYNLTLGS......SSSELLANIEAEKEKIDCLLVVSHPS....L
Microcystis a.  PCC 9807         28  NSLTALLANERYNLTLGS......SSSELLANIEAQKEKIDCLLVVFHPS....L
Microcystis sp.  T1-4           28  NSLTALLANERYNLTLGS......SSSELLANIEAQKEKIDCLLVVFHPS....L
Moorea p.  3L                   20  SLSQVLG.SEQYIIHSTS......SESEFFEVVEQHKQDLDCLVLQDTDT....L
Nodularia s.  CCY9414            1  .......................................................
Nostoc a.  0708                   1  .......................................................
Nostoc c.                         1  .......................................................
Nostoc p.  PCC 73102              1  .......................................................
Nostoc sp.  PCC 7120              1  .......................................................
Nostoc sp.  PCC 9709              1  .......................................................
Oscillatoria sp.  PCC 6506       43  TSVLKLLDRNRYILMRSN......SPEGFLDFIQ.RNSHLDCLVFEVYPE....L
Raphidiopsis b.  D9               1  .......................................................
Synechococcus e.  PCC 6301       18  QDCQRALSADRYQLQVCE......SGEMLLEYAQTHRDQIDCLILVAANP...SF
Synechococcus sp.  PCC 7335      18  EPLQQLLAQQQFVVSWSQ......SASEFEEWVVSNRHRIDCLIIQSAEE....S
Synechococcus sp.  BL107         19  DLCRQWLPANRYESVVLS....VGDNEGLATTLAPRHDDFDAVVIEQNLLD....
Synechococcus sp.  CB0101        19  RACRSWLKGGRYQLEDLG......SLADPILELQQQRDRFDVVLLQQGVHP....
Synechococcus sp.  CB0205        19  KAATSWLKGERYCLEDLG......SLSDPIAELLQRRDAFDVVLLQQGVTP....
Synechococcus sp.  CC9311        49  SACCQWLPGNRYTPVDLG...LEDSAVDVVSALERQREAVDAVVIEQSLLQ....
Synechococcus sp.  CC9605        19  EACRPWLPANRYESVVLT....VDPGETLAAVLGPRQDDFDAVVLEQSLLD....
Synechococcus sp.  CC9902        19  DTCRQWLPANRYESVVLS....VGENEGLATTLAPRHNDFDAVVVEQNLLD....
Synechococcus sp.  JA-2-3B       24  LACEDWLPPERFVVECFQ......DQATFLEALRKQDGHVDCLVLLVDPTRLQEL
Synechococcus sp.  JA-3-3Ab       1  ......MSPERFVAEVFQ......DPEAFLEALRGEENHPDCLVLLVEATRLDKL
Synechococcus sp.  PCC 7002      18  GKIEAQLIGDRYQIKLLD......YGQKFLAFLEANKEQIDCLVVVQGKE....N
Synechococcus sp.  RCC307        19  KSCRQWLREADRFRLEVIP....LNPESLFDALQERWEDLDGVLIEQGALD....
Synechococcus sp.  RS9916        19  PACRSWLPENRYTVVDLG...LTGGVTDFQAALEQQREAVDAVVIEQSLLE....
Synechococcus sp.  RS9917        19  ETCRQWLPGNRYTPVDLG...LNRETTDLRQVLEGQREAVDAVVIEQSLLT....
Synechococcus sp.  WH 5701       19  QACSTWLPQGRYELMELG......LQQDPAAELERCWEQFDAVLLEPGVLS....
Synechococcus sp.  WH 7803       19  DACTQWLPVNRYKPVDLH...QAASGEGLLEVLAHQREAVDAVVIEQSLLD....
Synechococcus sp.  WH 7805       19  DACRQWLPVNRYKPVDLQ...QTASGQALLEVLSHQREAVDAVVIEQSLLD....
Synechococcus sp.  WH 8016       19  SACCQWLPGNRYTRVDLG...LEDSAVDVVSALERQREAVDAVVIEQSLLE....
Synechococcus sp.  WH 8102       19  DACQQWLPDTRYHSIVLSGPHQGQEQLDLVSTLEAQQEEIDAVVVEQQLLD....
Synechococcus sp.  WH 8109       19  EACRPWLPANRYESVVLT....VDPGETLAAVLGPRQNDFDAVVLEQSLLD....
Synechocystis sp.  PCC 6803      17  HRLRSIFQGDRHYLSTFQ......ALDDFCAFLEDKPERIDCLLVYYEAN....S
Thermosynechococcus e.  BP-1     21  ELVRLHTSDIDELVYFS.......SEREFCNYLEARRNSIACLILEWGEG....T
Trichodesmium e.  IMS101         39  TSVTKLLSQDRYLVALTK......SDREFVQLIEENTN.IDCLLFQEDPE....L
consensus                                       *                   *      * ***
```

logo

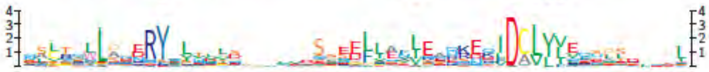

```
                                          α3          β4
                                             70        80        90
Synechococcus e.  PCC 7942          64   RAVVQQLCFEGVVVPAIVVGDRDSEDPD......................EPAKE
Acaryochloris m.  MBIC11017         70   PKVVSHLHREAILLPAVLLQVEESVEKTSQPNSAD..............RDPQQD
Acaryochloris sp.  CCMEE 5410       70   PKVVSHLHREAILLPAVLLQVEESVEKTSQPNSAD..............RDPQQD
Anabaena v.  ATCC 29413              1   .......................................................
Arthrospira m.  CS-328              55   QVLVDKLQQNLISIPTLIIIPNEQSPVVQPSLTEP.................SEN
Arthrospira m.  FACHB-438           55   QGLVNKLQQNLVSIPTLIIISDEQSPVVQPSLTEP.................SEN
Arthrospira p.  str.  Paraca        55   QGLVNKLQQNLVSIPTLIIISDEQSPVVQPSLTEP.................SEN
Crocosphaera w.  WH 0003            44   LPLFNQLYERGTLLPVIIVE..KGEDPS.................IALANVESPT
Crocosphaera w.  WH 8501            71   LPLFNQLYERGTLLPVIIVE..KGEDPS.................IALANVESPT
Cyanobium sp.  PCC 7001             64   PSVFAGLNQRGLVLPAVVIGAVTG..............................E
Cyanothece sp.  ATCC 51142          63   LPLFNQLYEQGTLVPVIIFE..DKVES....................SDNVEPPT
Cyanothece sp.  CCY0110             44   FPLFNQLYEQGTLLPIIIFE..EEDSS..................ASSVNVDSPT
Cyanothece sp.  PCC 7424            63   LPLFNQLYEEGILLPIVLLETPIENTAPMTGGQ.....ENGEENQKAAKLSEAPT
Cyanothece sp.  PCC 7425            64   PQVACYLHKEATLLPAIVLLSNP...SPSAASESD..............KLWHED
Cyanothece sp.  PCC 7822            63   LPLFNQLYEEGVLLPVVLLESPSTSLSELYNATPPAIAESRLPEESPSSPKDAPT
Cyanothece sp.  PCC 8801            63   SPLFNQLYEQGTLLPVIILDSELDSQAS.................TQSENMETAT
Cyanothece sp.  PCC 8802            63   SPLFNQLYEQGTLLPVIILDSELDSQAS.................TQSENMETAT
Cylindrospermopsis r.  CS-505        1   .............................................MILVILFLYP
Fischerella sp.  JSC-11              1   .......................................................
Leptolyngbya b.  IAM M-101          64   PAIIEWLHSQATLLPAILVESEPHTTR.......................PPDFT
Lyngbya sp.  PCC 8106               43   TVLVDQFRQQGISLPIVILDSQIQSSQPTAQSETSGST.........QSFSDSPN
Microcoleus c.  PCC 7420            63   PPVINQLYEQGTLLPLVIFPKESKNNDSFVVTNAQSNPI....QTTVRCTPGEVH
Microcoleus v.  FGP-2               62   RSLLGKLQERSLFLPAVIFSPHLQVDGRPEAPYSDGFEAV..ASTANKSQDESCA
Microcystis a.  NIES-843            54   QPTFNQLYEGGILLPVVIIVADKN.....................ITAKTNDSPT
Microcystis a.  PCC 7806            69   QPTFNQLYEGGILLPVVIIVADKN.....................IAPEMNDSPT
Microcystis a.  PCC 7820            54   QPTFNQLYEGGILLPVVIIVADKN.....................IAPEMNDSPT
Microcystis a.  PCC 7941           100   QPTFNQLYEGGILLPVVIIVADKN.....................IAPEMNDSPT
Microcystis a.  PCC 9443            73   QPTFNQLYEGGILLPVVIIVADKN.....................IAPETNDSPT
Microcystis a.  PCC 9701            73   QPTFNQLYEGGILLPVVIIVADKN.....................ITAETNDSPT
Microcystis a.  PCC 9717            73   QPTFNQLYEGGILLPVVIIVADKN.....................ITAETNDSPT
Microcystis a.  PCC 9806           101   QPTFNQLYEAGILLPVVIIVADNN.....................ITAGTNDSPT
Microcystis a.  PCC 9807            73   QPTFNQLYEGGILLPVVIIVADKN.....................IAPETNDSPT
Microcystis sp.  T1-4               73   QPTFNQLYEAGVLLPVVIIVADKN.....................ITAGTNDSPT
Moorea p.  3L                       64   PKVIHYLYLQGTTLPAVFLRSDSPEIPPRVDSNQQLASN....QTTDKFTARSSA
Nodularia s.  CCY9414                1   .......................................................
Nostoc a.  0708                      1   .....................................M.......ILPILLIWLL
Nostoc c.                            1   .............................................MLLPILFFQP
Nostoc p.  PCC 73102                 1   .............................................MLLPILILRP
Nostoc sp.  PCC 7120                 1   .......................................................
Nostoc sp.  PCC 9709                 1   .............................................MLLPILILPP
Oscillatoria sp.  PCC 6506          87   HLLVSQLQEHSIFLPVVILEAEANSEDAKLLPENQ.......AMTSEDSHEENLH
Raphidiopsis b.  D9                  1   .......................................................
Synechococcus e.  PCC 6301          64   RAVVQQLCFEGVVVPAIVVGDRDSEDPD......................EPAKE
Synechococcus sp.  PCC 7335         63   LQVLLHLKEKDILLPAVALYSSKAPSTHSELSDSAVFL.......QGQRTYHQAV
Synechococcus sp.  BL107            66   SDAREDLLKAGLLFPAVIVGEVKG..............................H
Synechococcus sp.  CB0101           64   PEVYEELQQQGLLLPAVVIGEVSG..............................R
Synechococcus sp.  CB0205           64   PEALQGLRQQGVLLPAVVVGEVNG..............................R
Synechococcus sp.  CC9311           97   EQIREDLLARGLLFPAVVVGELMG..............................R
Synechococcus sp.  CC9605           66   AEVKEQLLAAGLLFPAVIVGEVKG..............................Q
Synechococcus sp.  CC9902           66   SEAREDLLKAGLLFPAVIVGEVKG..............................H
Synechococcus sp.  JA-2-3B          73   HDLGNQLCAWGLLLPTVLAIVLEESHLQKETP..ETEGEIPATSSLVKALLKREQ
Synechococcus sp.  JA-3-3Ab         44   EALGCQLCKQGLLLPTVVAIVSEELKPEGGASR.SGEASSPPVPPGARALLKRER
Synechococcus sp.  PCC 7002         63   QDYFECLTQSGILLPCVFLGPTTACEIS.....................QEDVSE
Synechococcus sp.  RCC307           66   RHGLDQLRTQGMLLPAVVLGDVTG..............................E
Synechococcus sp.  RS9916           67   PDLRRSLLEAGLLFPAVVVGEVMG..............................R
Synechococcus sp.  RS9917           67   PEVRQSLLEAGLLFPAVVVGEVMG..............................H
Synechococcus sp.  WH 5701          64   IEAFQRLRAQGRILPAVVLGQLSG..............................P
Synechococcus sp.  WH 7803          67   ETTREGLRRGGLLFPAVVVGELMG..............................R
Synechococcus sp.  WH 7805          67   ESTREGLRREGLLFPAVVVGELMG..............................R
Synechococcus sp.  WH 8016          67   EQTREDLLARGLLFPAVVVGELMG..............................R
Synechococcus sp.  WH 8102          70   ASSRDQLLGRGLLFPAVVVGEMKG..............................H
Synechococcus sp.  WH 8109          66   AEAKEQLLASGLLFPAVIVGEVKG..............................Q
Synechocystis sp.  PCC 6803         62   LPVLNRLYEQGRLLPIILLEPSPS....................ALAKTTDEHPT
Thermosynechococcus e.  BP-1        65   PQIITYLHHSATLLPAILIFPAA...PAPPPAG......................
Trichodesmium e.  IMS101            83   TSLIHKLHHRSILLPAVIIKKSESENSLENQQLTDTDNN....FTEYTTQHNYQP
consensus                                      *       * ***
```

logo

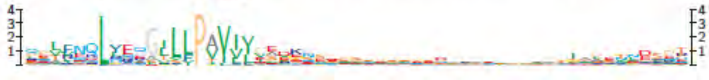

```
                                                  β5                      α4
                                        100                 110       120       130
Synechococcus e.  PCC 7942          97  QLYHSAELHLGIH................QLEQLPYQVDAALAEFLRLAPVETMA
Acaryochloris m.  MBIC11017        111  SYYHIAEVIVDHD................QEDILS.SIDCAIAQFLQLSKACRLP
Acaryochloris sp.  CCMEE 5410      111  SYYHIAEVIVDHD................QEDILS.SIDCAIAQFLQLSKACRLP
Anabaena v.  ATCC 29413              1  .......................................................
Arthrospira m.  CS-328              93  YILSSTEVQLKGK................QIHLIQGYIDKAISQFITHAVTTRLT
Arthrospira m.  FACHB-438           93  YILSSTEVQLKGQ................QIHVIQGYIDKAISQFITHAVTTRLT
Arthrospira p.  str.  Paraca        93  YILSSTEVQLKGQ................QIHLIQGYIDKAISQFITHAVTTRLT
Crocosphaera w.  WH 0003            80  FLYHSAEVRHGVS................QLGDVQMTIDRAITKFLHLGPSCCLA
Crocosphaera w.  WH 8501           107  FLYHSAEVRHGVS................QLGDVQMTIDRAITKFLHLGPSCCLA
Cyanobium sp.  PCC 7001             89  VTVHEAEVRLPPD................QLEQLPYSLDAALSRFLRRGVMNPAD
Cyanothece sp.  ATCC 51142          96  FLYHSAEVRYRVK................ELDEIQVTIDRAITKFLHLGPSCFLT
Cyanothece sp.  CCY0110             80  FLYHSAEVHHQAK................EWDDIQVIIDRAITKFLHLGPSCFLS
Cyanothece sp.  PCC 7424           113  CLYHSAEVRVKVN................ELEDISSIIDQAITRFLHLGPNCSVT
Cyanothece sp.  PCC 7425           102  IFYHTAELQLPLE................SLPQISSHIEQAIARFLQLTAACRLP
Cyanothece sp.  PCC 7822           118  FLYHSAEIRFKIN................ELEDIASVIDQAITRFLHLGPNCSIS
Cyanothece sp.  PCC 8801           101  CLYHTAEVRYRVT................ALNKIESVIEEAIAQFLHLSPSCSLA
Cyanothece sp.  PCC 8802           101  CLYHTAEVRYRVT................ALNKIESVIEEAIAQFLHLSPSCSLA
Cylindrospermopsis r.  CS-505       11  ALEKLLYSLVTHY................DNLINTLIILTPGSFISVSMSNVSIN
Fischerella sp.  JSC-11              1  .......................................................
Leptolyngbya b.  IAM M-101          96  FLYHTGEVWATLA................DLEQVDNLIANAIEGFLNLSPSCQIP
Lyngbya sp.  PCC 8106               89  YIYHSSEVHLGVN................QLNQISGYVDKAISQFITQAVTTRLT
Microcoleus c.  PCC 7420           114  YLFHAAEVRLDSS................QVSDLEHALEQAITQFLSLSPNCNLP
Microcoleus v.  FGP-2              115  FFYHPGEVQLALA................RSSEIAAGIDRAIAQFLKLSTPVPVN
Microcystis a.  NIES-843            88  CLYHSAELQITVN................ELDSITSVIDQAIARFLHLAPNCSFS
Microcystis a.  PCC 7806           103  CLYHSAELQITVK................ELDSITSVIDQAIARFLHLAPNCSFS
Microcystis a.  PCC 7820            88  CLYHSAELQITVK................ELDSITSVIDQAIARFLHLAPNCSFS
Microcystis a.  PCC 7941           134  CLYHSAELQITVK................ELDSITSVIDQAIARFLHLAPNCSFS
Microcystis a.  PCC 9443           107  CLYHSAELQITVK................ELDGITSVIDQAIARFLHLAPNCSFS
Microcystis a.  PCC 9701           107  CLYHSAELQITVN................ELDSITAVIDQAIARFLHLAPNCSFS
Microcystis a.  PCC 9717           107  CLYHSAELQITVK................ELDSITSVIDQAIARFLHLAPNCSFS
Microcystis a.  PCC 9806           135  CLYHSAELQITVK................ELDSITAVIDQAIARFLHLAPNCSFF
Microcystis a.  PCC 9807           107  CLYHSAELQITVK................ELDGITSVIDQAIARFLHLAPNCSFS
Microcystis sp.  T1-4              107  CLYHSAELQITVK................ELDSITSVIDQAIARFLHLAPNCSFS
Moorea p.  3L                      115  HLFHPAEVELSIH................QLPEIGDFIQQAMTEFLSLAPACNLP
Nodularia s.  CCY9414                1  .......................................................
Nostoc a.  0708                     12  GVKKYLYNIAIPN................QQEGFIEALNNVIDDIVNLFFGSQLT
Nostoc c.                           11  NVKKILDNLDELG................IQIPLNPNNGYILQSNFAQFSTSTLV
Nostoc p.  PCC 73102                11  DVNKCLDNLVDLA................KQKGWISWVWKFIDAIVAQFCYLPVT
Nostoc sp.  PCC 7120                 1  .......................................................
Nostoc sp.  PCC 9709                11  DVNKSLNNLVDLA................NQKALIPWVWKLIDATISQFSSLPVA
Oscillatoria sp.  PCC 6506         135  FFYHAAEVKLPIA................KLSDLSSFIDQAMTRFLNLSVPVRAR
Raphidiopsis b.  D9                  1  .......................................................
Synechococcus e.  PCC 6301          97  QLYHSAELHLGIH................QLEQLPYQVDAALAEFLRLAPVEAMA
Synechococcus sp.  PCC 7335        111  VASDIRNLGAVDD................AIYQAVDSYLQLPTEVNADDLDQVAI
Synechococcus sp.  BL107            91  VDYHQEELHLPED................QLAQLGYNVDAAISRFLRQGRADGRQ
Synechococcus sp.  CB0101           89  VEYHEAEVHLPQD................QLEQIAYSVDAAVSRFLRRGMQATPT
Synechococcus sp.  CB0205           89  VDYHEAEVHLPQD................QLEQVVYSLDAAVSRFLRKGLSAQSE
Synechococcus sp.  CC9311          122  VDYHPEEVHLPGD................QLEQLGYNVDAAISRFLRHGQKDIRP
Synechococcus sp.  CC9605           91  VDYHPEELHLPDD................QLAQLGYNVDAAISRFLRQGRADGRQ
Synechococcus sp.  CC9902           91  VDYHQEELHLPED................QLAQLGYNVDAAISRFLRQGRADGRQ
Synechococcus sp.  JA-2-3B         126  FFYHNATLVRPISPQELKGQVRADCTASGSPSVLELLVDRAIALFLQMSPTCGLP
Synechococcus sp.  JA-3-3Ab         98  FFYHNATLVRLVSPSELLPPKGSQATPSASP..LELLINRAIALFLQISPTCSLP
Synechococcus sp.  PCC 7002         97  QLYHSAEKYLDFA................NLENLSLTIDQAIAQFLHLAPSCALS
Synechococcus sp.  RCC307           91  VDYHDAEVHLQAD................QLEQLPYNLDAAVLRFLERSQAKGRS
Synechococcus sp.  RS9916           92  VDYHPEEVHLPAD................QLEQLGYNVDAAISRFLRHGQKEELH
Synechococcus sp.  RS9917           92  VDYHPEEVHLPVD................QLEQLGYNVDAAISRFLRHGQREDRP
Synechococcus sp.  WH 5701          89  MEFFQDEVHLPPD................QMEQLSYSLDAAISRCLRRRSSSGSG
Synechococcus sp.  WH 7803          92  VDYHPEEVHLPVD................QLEQLGYNVDAAISRFLRQGQKEARP
Synechococcus sp.  WH 7805          92  VDYHPEEVHLPVD................QLEQLGYNVDAAISRFLRQGQKEARP
Synechococcus sp.  WH 8016          92  VDYHPEEVHLPGD................QLEQLGYNVDAAISRFLRHGQKDIRP
Synechococcus sp.  WH 8102          95  VDYHAEELHLAED................QLAQLGYTVDAAISRFLRQGRADGRS
Synechococcus sp.  WH 8109          91  VDYHPEELHLPDD................QLAQLGYNVDAAISRFLRQGRADGRQ
Synechocystis sp.  PCC 6803         97  IVYHNAEIHLPES................QWSELPTVVDRAIAHYLHLGPICTLP
Thermosynechococcus e.  BP-1        95  PHYHIAEVILTTD................QLDQLNRQIEEAITGFVKLCPGCAVP
Trichodesmium e.  IMS101           134  FIYHQAEASLLIN................QLGKIDFYVEQAIQKFLKLSPASSSK
consensus                                 **  **                          *   ** **   **
```

logo

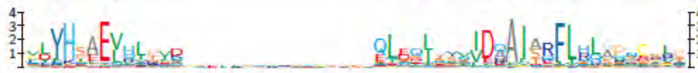

```
                                                                             α5            β6      α6
                                         140                  150           160         170
Synechococcus e.  PCC 7942          136  DHIMLMG....ANHDP...........ELSSQQRDLAQRLQERLGYLGVYYKRDP
Acaryochloris m.  MBIC11017         149  TRLQKKYADEAIQDN.............LATQQQRLSQKLKERLGYLGVYYKRNP
Acaryochloris sp.  CCMEE 5410       149  TRLQKKYADEAIQDN.............LATQQQRLSQKLKERLGYLGVYYKRNP
Anabaena v.  ATCC 29413               1  .......................................................
Arthrospira m.  CS-328              132  DES..SSVDTPTELTN...........FIVRQQRRLMDKLHERLGYLGVYYKRNP
Arthrospira m.  FACHB-438           132  DES..SSVDTPTELTN...........FIVRQQRRLMDKLHERLGYLGVYYKRNP
Arthrospira p.  str.  Paraca        132  DES..SSVDTPTELTN...........FIVRQQRRLMDKLHERLGYLGVYYKRNP
Crocosphaera w.  WH 0003            119  DRSLSAQGDIDTEKHQ..........SFLLLQQRRLAEKLKERLGYLGVYYKRNP
Crocosphaera w.  WH 8501            146  DRSLSAQGDIDTEKHQ..........SFLLLQQRRLAEKLKERLGYLGVYYKRNP
Cyanobium sp.  PCC 7001             128  GEEAAEAGPASGDAAAGREAGEAGETGAPVPMPWRLDDRLRERLGLLGVFYKRDP
Cyanothece sp.  ATCC 51142          135  DQSLSSQRDIGTEKNQ..........SFLLLQQRRLAEKLKERLGYLGVYYKRNP
Cyanothece sp.  CCY0110             119  EEPSSSQRDIRVENNQ..........SFLLLQQRRLAEKLKERLGYLGVYYKRNP
Cyanothece sp.  PCC 7424            152  DQSLIRSKPNQVEDKQ..........NFLLLQQRRLAEKLKERLGYLGVYYKRNS
Cyanothece sp.  PCC 7425            141  LVVPMASS.PEMQDS.............LNQQQQNLSEKLKERLGYLGLYYKRDP
Cyanothece sp.  PCC 7822            157  EQSLSRSKPNLIEEKQ..........NFLLLQQRRLAEKLKERLGYLGVYYKRNS
Cyanothece sp.  PCC 8801            140  NAPVSPLKIIPSPEKQP.........SFLLLQQRRLAEKLKERLGYLGVYYKRNP
Cyanothece sp.  PCC 8802            140  NAPVSPLKIIPSPEKQP.........SFLLLQQRRLAEKLKERLGYLGVYYKRNP
Cylindrospermopsis r.  CS-505        50  NRSGISGLELPGTYLL...........NYTKEKRYLGLDYKEGYTLVLACENSHT
Fischerella sp.  JSC-11               1  ..............................................MNGCQQPKQ
Leptolyngbya b.  IAM M-101          135  RETAPPDPTRDLTTQN...........FLMLQQRRLTEKLHERLGYLGVYYKRNP
Lyngbya sp.  PCC 8106               128  DDS..LDVDTPTELTN...........FIMRQQKRLADKLHERLGYLGVYYKRSS
Microcoleus c.  PCC 7420            153  TLLANADKKTEEIHRG...........SLMKQQYRLSEKLKERLGYLGVYYKRNP
Microcoleus v.  FGP-2               154  YQSVTVDATAQLTAQS...........LLSRQQRRLAEKLRERLGYLGVYYKRNS
Microcystis a.  NIES-843            127  EPTTIVNQPNPVENNH..........SFLLLQQRRLAEKLKERLGYLGVYYKRNP
Microcystis a.  PCC 7806            142  ERTTIVNQPNPVENNH..........SFLLLQQRRLAEKLKERLGYLGVYYKRNP
Microcystis a.  PCC 7820            127  ERTTIVNQPNPVENNH..........SFLLLQQRRLAEKLKERLGYLGVYYKRNP
Microcystis a.  PCC 7941            173  ERTTIVNQPNPVENNH..........SFLLLQQRRLAEKLKERLGYLGVYYKRNP
Microcystis a.  PCC 9443            146  ERTTIVNQPNPVANNH..........SFLLLQQRRLAEKLKERLGYLGVYYKRNP
Microcystis a.  PCC 9701            146  ERTTIVNQPNPVANNH..........SFLLLQQRRLAEKLKERLGYLGVYYKRNP
Microcystis a.  PCC 9717            146  EPTTIVNQPNPVENNH..........SFLLLQQRRLAEKLKERLGYLGVYYKRNP
Microcystis a.  PCC 9806            174  ERTTIVNQPNPVANNH..........SFLLLQQRRLAEKLKERLGYLGVYYKRNP
Microcystis a.  PCC 9807            146  ERTTIVNQPNPVANNH..........SFLLLQQRRLAEKLKERLGYLGVYYKRNP
Microcystis sp.  T1-4               146  ERTTIVNQPNPVANNH..........SFLLLQQRRLAEKLKERLGYLGVYYKRNP
Moorea p.  3L                       154  RSSGPVYPNLAVANYS...........FLSKQQHRLAEKLRERLGYLGVYYKRNP
Nodularia s.  CCY9414                 1  .......................................................
Nostoc a.  0708                      51  PTSAAK.....IHQLH...........NTRQLNTNQTGDNNTQYTYVLACNYQKN
Nostoc c.                            50  AATTAR.....LYYLL...........NWLPANLN..QNFIDQYFYVFACQQQKP
Nostoc p.  PCC 73102                 50  ATTSAT.....INYLP...........NWPQQNP..SKAYTSKYVYVFASQMQKS
Nostoc sp.  PCC 7120                  1  .......................................................
Nostoc sp.  PCC 9709                 50  ATTSEK.....INYLP...........NWLQQSP..SKAYTGKYVYVFASQMQKS
Oscillatoria sp.  PCC 6506          174  GHASTTDPTAELTSQS...........LLHRQQRRLAEKLRERLGYLGVYYKRNS
Raphidiopsis b.  D9                   1  .......................................................
Synechococcus e.  PCC 6301          136  DHIMLMG....ANHDP...........ELSSQQRDLAQRLQERLGYLGVYYKRDP
Synechococcus sp.  PCC 7335         150  GSAESAVSHDRDKRLF...........LLSLQQQRLTEKLKERLGYVGVYYKRNR
Synechococcus sp.  BL107            130  ED..TATKAVGS.......................LSRRLQERLGYLGVFYKRDP
Synechococcus sp.  CB0101           128  GVGEGTGGDTPER....................WRLPNRLKGRLGYLGVFYKRDP
Synechococcus sp.  CB0205           128  SGSE..SASTPLS....................WRLPNRLQGRLGYLGVFYKRDP
Synechococcus sp.  CC9311           161  EDGSSESDQVGGQPEG...............SAWKLSSRLQERLGYLGVFYKRDP
Synechococcus sp.  CC9605           130  DDSSSSSRAVSN.......................LSDRLQERLGYLGVFYKRDP
Synechococcus sp.  CC9902           130  ED..TATKAVGS.......................LSRRLQERLGYLGVFYKRDP
Synechococcus sp.  JA-2-3B          181  QGPVHPSDQPYLAYSN..............QQQRRLAEKLRERLGYAGVYYHRDP
Synechococcus sp.  JA-3-3Ab         151  AANPHPKDQPYLLYTH..............QQQRRLAEKLRERLGYAGVYYHREP
Synechococcus sp.  PCC 7002         136  DKPQDPHHSDPDKTHQ.........AFLLLQQRRLAEKLRERLGYLGVYYKRNP
Synechococcus sp.  RCC307           130  PSPTELPQGSDQ.....................WRLSQRLEERIGFLGVFYKRDP
Synechococcus sp.  RS9916           131  GEGQALDVEGGVLVEA...............SAWRLTSRLQERLGYLGVFYKRDP
Synechococcus sp.  RS9917           131  SGGTAVDVEGGAAVEA...............SAWRLTSRLQERLGYLGVFYKRDP
Synechococcus sp.  WH 5701          128  GGLEASDSHAGESDQ................SNWRLPHRLRERLGYLGIFYKRDS
Synechococcus sp.  WH 7803          131  EDGS.APSSAES..AS...............SAWKLSSRLQERLGYLGVFYKRDP
Synechococcus sp.  WH 7805          131  EDGSDANTQPSG..ES...............SAWKLSSRLQERLGYLGVFYKRDP
Synechococcus sp.  WH 8016          131  EDGSAESDQVGGQPEG...............SAWKLSSRLQERLGYLGVFYKRDP
Synechococcus sp.  WH 8102          134  DD..DGLASVDK.......................LSRRLQERLGYLGVFYKRDP
Synechococcus sp.  WH 8109          130  DESSSSSRAVSN.......................LSDRLQERLGYLGVFYKRDP
Synechocystis sp.  PCC 6803         136  NQTETIPAPIVDESSQ..........SFLLLQQRRLADKLKERLGYLGVYYKRKP
Thermosynechococcus e.  BP-1        134  PHVLFRLP.ALKESS.............NVDPQHRLSQKLKERLGYLGVYYKRDT
Trichodesmium e.  IMS101            173  DKSQISSQATDDYTKN...........ILHQQQHRLYAKLRERLGYLGVYYKRNP
consensus                                                                   *  ** ************
```

logo

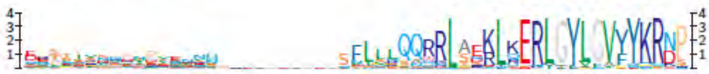

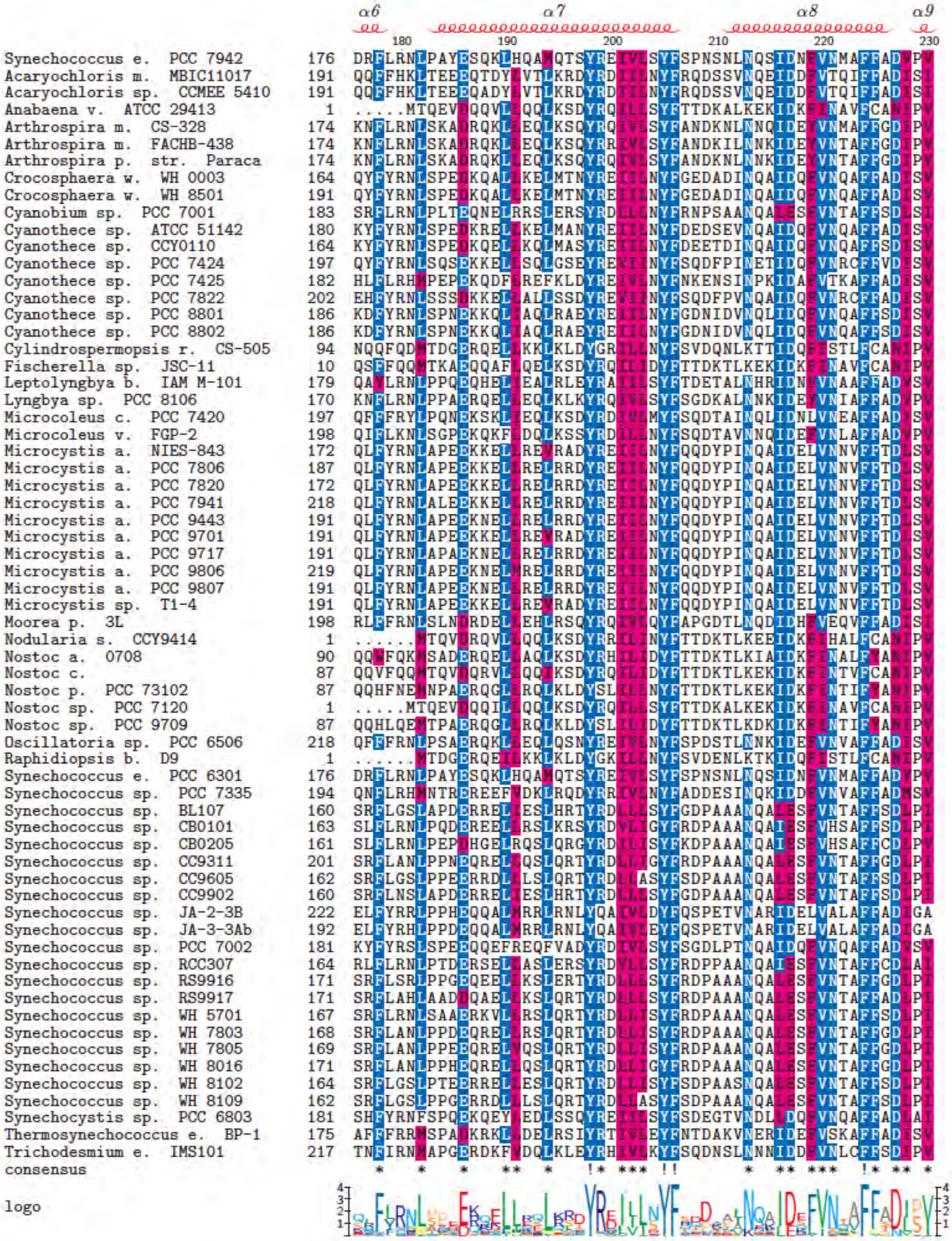

α6 α7 α8 α9
180 190 200 210 220 230
Synechococcus e. PCC 7942 176 DRFLRNLPAYESQKLHQAMQTSYREIVLSYFSPNSNLNQSIDNFVNMAFFADVPV
Acaryochloris m. MBIC11017 191 QQFFHKLTEEEQTDYLVTLKRDYRDIILNYFRQDSSVNQEIDDFVTQIFFADISI
Acaryochloris sp. CCMEE 5410 191 QQFFHKLTEEEQADYLVTLKRDYRDIILNYFRQDSSVNQEIDDFVTQIFFADISI
Anabaena v. ATCC 29413 1 .....MTQEVDQQVLLQQLKSDYRQILLSYFTTDKALKEKIDKFINAVFCANIPV
Arthrospira m. CS-328 174 KNFLRNLSKADRQKLLEQLKSQYRQIVLSYFANDKNLNNQIDEYVNMAFFGDIPV
Arthrospira m. FACHB-438 174 KNFLRNLSKADRQKLLEQLKSQYRRIVLSYFANDKILNNKIDEYVNTAFFGDIPV
Arthrospira p. str. Paraca 174 KNFLRNLSKADRQKLLEQLKSQYRQIVLSYFANDKNLNNKIDEYVNTAFFGDIPV
Crocosphaera w. WH 0003 164 QYFYRNLSPEDKQALLKELMTNYREIILNYFGEDADINQAIDQFVNQAFFADISV
Crocosphaera w. WH 8501 191 QYFYRNLSPEDKQALLKELMTNYREIILNYFGEDADINQAIDQFVNQAFFADISV
Cyanobium sp. PCC 7001 183 SRFLRNLPLTEQNELRRSLERSYRDLLLNYFRNPSAANQALESFVNTAFFSDLSI
Cyanothece sp. ATCC 51142 180 KYFYRNLSPEDKRELLKELMANYREIILNYFDEDSEVNQAIDQFVNQAFFADISV
Cyanothece sp. CCY0110 164 KYFYRNLSPEDKQELLKQLMASYREIILNYFDEETDINQAIDQFVNQAFFSDISV
Cyanothece sp. PCC 7424 197 QYFYRNLSQSEKKELLSQLGSEYREVIINYFSQDFPINETIDQFVNRCFFVDISV
Cyanothece sp. PCC 7425 182 HLFLRHMPEPEKQDFLREFKLDYREIVLNYFNKENSINPKIDAFVTKAFFADISV
Cyanothece sp. PCC 7822 202 EHFYRNLSSSDKKELLALLSSDYREVIINYFSQDFPVNQAIDQFVNRCFFADISV
Cyanothece sp. PCC 8801 186 KDFYRNLSPNEKKQLIAQLRAEYREIILNYFGDNIDVNQLIDQFVNQAFFSDISV
Cyanothece sp. PCC 8802 186 KDFYRNLSPNEKKQLIAQLRAEYREIILNYFGDNIDVNQLIDQFVNQAFFSDISV
Cylindrospermopsis r. CS-505 94 NQQFQDMTDGERQELLKKLKLDYGRILLNYFSVDQNLKTTIDQFISTLFCANIPV
Fischerella sp. JSC-11 10 QSFFQQMTKAEQQAFLQELKSDYRQILIDYFTTDKTLKEKIDKFINAVFCANIPV
Leptolyngbya b. IAM M-101 179 QAYLRNLPPQEQHELIEALRLEYRAIILSYFTDETALNHRIDNFVNAAFFADVSV
Lyngbya sp. PCC 8106 170 KNFLRNLPPAERQELLEQLKLKYRQIVLSYFSGDKALNNKIDEYVNIAFFADVPV
Microcoleus c. PCC 7420 197 QFFFRYLPQNEKSKLIEQLKSDYRDIVLMYFSQDTAINQLIDNLVNEAFFADISV
Microcoleus v. FGP-2 198 QIFLKNLSGPEKQKFLDQLKSSYRDILLNYFSQDTAVNNQIDEFVNLAFFADVPV
Microcystis a. NIES-843 172 QLFYRNLAPEEKKELLREVRADYREIILNYFQQDYPINQAIDELVNNVFFTDLSV
Microcystis a. PCC 7806 187 QLFYRNLAPEEKKELLRELRRDYREIILNYFQQDYPINQAIDELVNNVFFTDLSV
Microcystis a. PCC 7820 172 QLFYRNLAPEEKKELLRELRRDYREIILNYFQQDYPINQAIDELVNNVFFTDLSV
Microcystis a. PCC 7941 218 QLFYRNLALEEKKELLRELRRDYREIILNYFQQDYPINQAIDELVNNVFFTDLSV
Microcystis a. PCC 9443 191 QLFYRNLAPEEKNELLRELRRDYREIILNYFQQDYPINQAIDELVNNVFFTDLSV
Microcystis a. PCC 9701 191 QLFYRNLAPEEKKELLREVRADYREIILNYFQQDYPINQAIDELVNNVFFTDLSV
Microcystis a. PCC 9717 191 QLFYRNLAPAEKNELLRELRRDYREIILNYFQQDYPINQAIDELVNNVFFTDLSV
Microcystis a. PCC 9806 219 QLFYRNLAPEEKNELMRELRRDYREIILNYFQQDYPINQAIDELVNNVFFTDLSV
Microcystis a. PCC 9807 191 QLFYRNLAPEEKNELLRELRRDYREIILNYFQQDYPINQAIDELVNNVFFTDLSV
Microcystis sp. T1-4 191 QLFYRNLAPEEKKELLREVRADYREIILNYFQQDYPINQAIDELVNNVFFTDLSV
Moorea p. 3L 198 RLFFRNLSLNDRDELLEHLRSQYRQIVLQYFAPGDTLNQDIDHFVEQVFFADISI
Nodularia s. CCY9414 1 ......MTQVDRQVLLQQLKSDYRRILINYFTTDKTLKEEIDKFIHALFCANIPV
Nostoc a. 0708 90 QQWFQKMSADERQELLAQLKSDYRHILIDYFTTDKTLKIAIDKFINALFYANIPV
Nostoc c. 87 QQVFQQMTQVDQRVLLQQIKSDYRQILIDYFTTDKTLKEKIDKFINTVFCANIPV
Nostoc p. PCC 73102 87 QQHFNEMNPAERQGLLRQLKLDYSLILLNYFTTDKTLKEKIDKFINTIFYANIPV
Nostoc sp. PCC 7120 1 .....MTQEVDQQILLQQLKSDYRQILLSYFTTDKALKEKIDKFINAVFCANIPV
Nostoc sp. PCC 9709 87 QQHLQEMTPAERQGLLRQLKLDYSLILIDYFTTDKTLKDKIDKFINTIFYANIPV
Oscillatoria sp. PCC 6506 218 QFFFRNLPSAERQKLLEQLQSNYREIVLNYFSPDSTLNNKIDEFVNVAFFADISV
Raphidiopsis b. D9 1 ......MTDGERQEILKKLKLDYGKILLNYFSVDENLKTKIDQFISTLFCANIPV
Synechococcus e. PCC 6301 176 DRFLRNLPAYESQKLHQAMQTSYREIVLSYFSPNSNLNQSIDNFVNMAFFADVPV
Synechococcus sp. PCC 7335 194 QNFLRHMNTREREEFVDKLRQDYRRIVLNYFADDESINQKIDDFVNVAFFADMSV
Synechococcus sp. BL107 160 SRFLGSLAPDERRELIESLHRTYRDLLLSYFGDPAAANQALESFVNTAFFSDLPI
Synechococcus sp. CB0101 163 SLFLRNLPQDEREELLRSLKRSYRDVLIGYFRDPAAANQAIESFVHSAFFSDLPI
Synechococcus sp. CB0205 161 SLFLRNLPEPDHGELRQSLQRGYRDILISYFKDPAAANQAIESFVHSAFFCDLPV
Synechococcus sp. CC9311 201 SRFLANLPPNEQRELLQSLQRTYRDLLIGYFRDPAAANQALESFVNTAFFGDLPI
Synechococcus sp. CC9605 162 SRFLGSLPPEERRDLLLSLQRTYRDLLASYFSDPAAANQALESFVNTAFFSDLPI
Synechococcus sp. CC9902 160 SRFLNSLAPDERRELIESLHRTYRDLLLSYFGDPAAANQALESFVNTAFFSDLPI
Synechococcus sp. JA-2-3B 222 ELFYRRLPPHEQQALMRRLRNLYQAIVLDYFQSPETVNARIDELVALAFFADIGA
Synechococcus sp. JA-3-3Ab 192 ELFYRHLPPDEQQALMRRLRNLYQAIVLEYFQSPETVNARIDELVALAFFADIGA
Synechococcus sp. PCC 7002 181 KYFYRSLSPEEQQEFREQFVADYRDIVLSYFSGDLPTNQAIDQFVNQAFFADVSV
Synechococcus sp. RCC307 164 RLFLRNLPTDERSELLASLERSYRDVLLSYFRDPPAANQAIESFVNTAFFCDLAI
Synechococcus sp. RS9916 171 SRFLSRLPPGEQEELLKSLERTYRDLLLSYFRDPAAANQALESFVNTAFFGDLPI
Synechococcus sp. RS9917 171 SRFLAHLAADDQAELLKSLQRTYRDLLLSYFRDPAAANQALESFVNTAFFADLPI
Synechococcus sp. WH 5701 167 SRFLRNLSAAERKVLLRSLQRTYRDLLISYFRDPAAANQALESFVNTAFFSDLPI
Synechococcus sp. WH 7803 168 SRFLANLPPDEQRELLRSLQRTYRDLLISYFRDPAAANQALESFVNTAFFGDLPI
Synechococcus sp. WH 7805 169 SRFLANLPPEEQRELVQSLQRTYRDLLISYFRDPAAANQALESFVNTAFFGDLPI
Synechococcus sp. WH 8016 171 SRFLANLPPHEQRELLQSLQRTYRDLLIGYFRDPAAANQALESFVNTAFFGDLPI
Synechococcus sp. WH 8102 164 SRFLGSLPTEERRELLESLQRTYRDLLISYFSDPAASNQALESFVNTAFFSDLPI
Synechococcus sp. WH 8109 162 SRFLGSLPPGERRDLLLSLQRTYRDLLASYFSDPAAANQALESFVNTAFFSDLPI
Synechocystis sp. PCC 6803 181 SHFYRNFSPQEKQEYLEDLSSQYREIILSYFSDEGTVNDLLDQFVNQAFFADLAI
Thermosynechococcus e. BP-1 175 AFFFRRMSPADKRKLLDELRSIYRTIVLEYFNTDAKVNERIDEFVSKAFFADISV
Trichodesmium e. IMS101 217 TNFIRNMAPGERDKFVDQLKLEYRHIVLKYFSQDNSLNNNIDDFVNLCFFSDIPV
consensus * * * ** * !* *** !! * ** *** !* ** *
logo

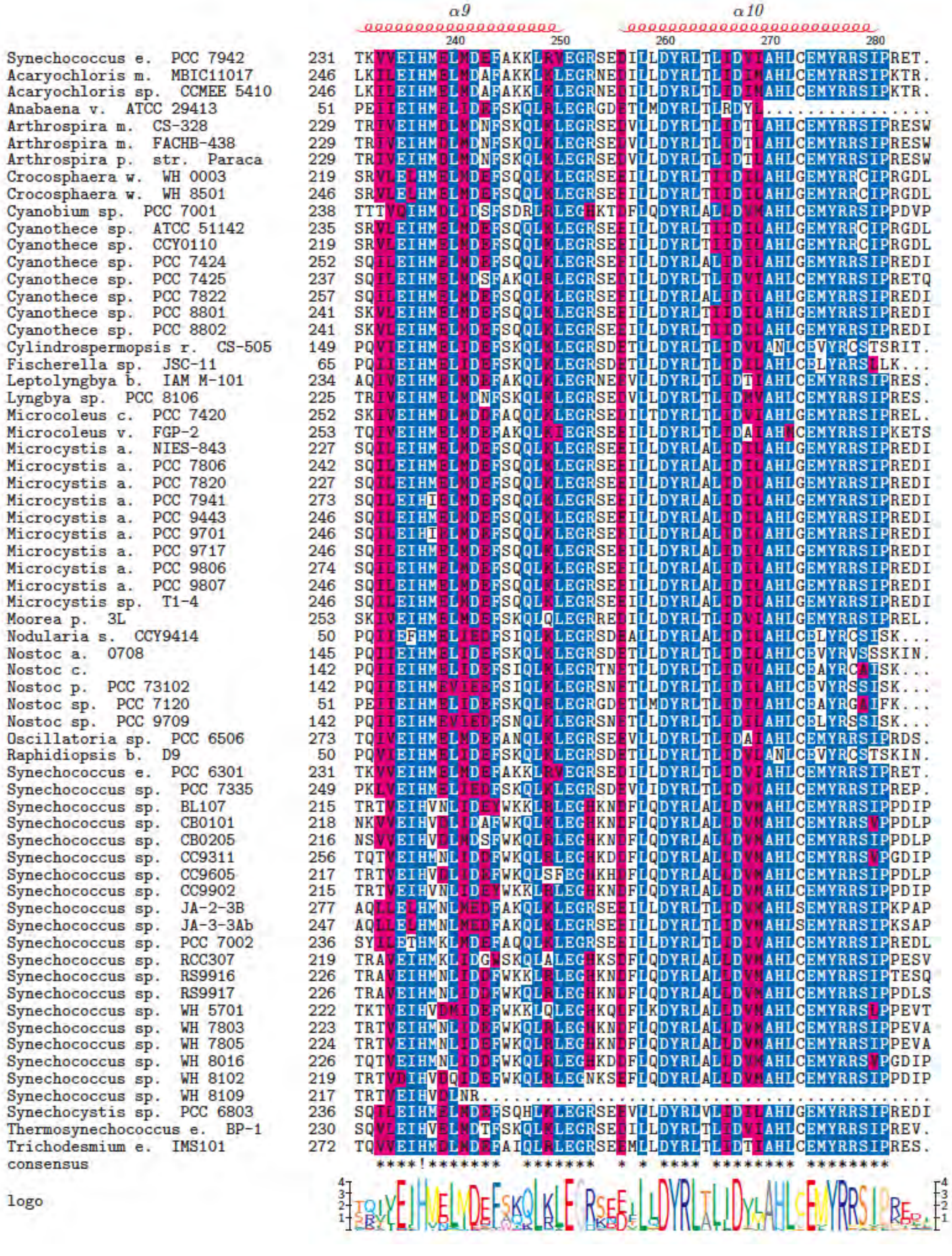

α9
α10
240
250
260
270
280
Synechococcus e. PCC 7942 231 TKVVEIHMELMDEFAKKLRVEGRSEDILLDYRLTLIDVIAHLCEMYRRSIPRET.
Acaryochloris m. MBIC11017 246 LKILEIHMELMDAFAKKLRLEGRNEDILLDYRLTLIDIMAHLCEMYRRSIPKTR.
Acaryochloris sp. CCMEE 5410 246 LKILEIHMELMDAFAKKLRLEGRNEDILLDYRLTLIDIMAHLCEMYRRSIPKTR.
Anabaena v. ATCC 29413 51 PEIIEIHMELIDEFSKQLRLEGRGDETLMDYRLTLRDYL..............
Arthrospira m. CS-328 229 TRIVEIHMDLMDNFSKQLRLEGRSEDVLLDYRLTLIDTLAHLCEMYRRSIPRESW
Arthrospira m. FACHB-438 229 TRIVEIHMDLMDNFSKQLRLEGRSEDVLLDYRLTLIDTLAHLCEMYRRSIPRESW
Arthrospira p. str. Paraca 229 TRIVEIHMDLMDNFSKQLRLEGRSEDVLLDYRLTLIDTLAHLCEMYRRSIPRESW
Crocosphaera w. WH 0003 219 SRVLELHMELMDEFSQQLRLEGRSEEILLDYRLTIIDILAHLGEMYRRCIPRGDL
Crocosphaera w. WH 8501 246 SRVLELHMELMDEFSQQLRLEGRSEEILLDYRLTIIDILAHLGEMYRRCIPRGDL
Cyanobium sp. PCC 7001 238 TTTVQIHMDLIDSFSDRLRLEGHKTDFLQDYRLALIDVMAHLCEMYRRSIPPDVP
Cyanothece sp. ATCC 51142 235 SRVLEIHMELMDEFSQQLRLEGRSEEILLDYRLTIIDILAHLGEMYRRCIPRGDL
Cyanothece sp. CCY0110 219 SRVLEIHMELMDEFSQQLRLEGRSEEILLDYRLTIIDILAHLGEMYRRCIPRGDL
Cyanothece sp. PCC 7424 252 SQILEIHMELMDEFSQQLRLEGRSEEILLDYRLALIDILAHLGEMYRRSIPREDI
Cyanothece sp. PCC 7425 237 SQILEIHMELMDSFAKQLRLEGRSEDILLDYRLTLIDVIAHLCEMYRRSIPRETQ
Cyanothece sp. PCC 7822 257 SQILEIHMELMDEFSQQLRLEGRSEEILLDYRLALIDILAHLGEMYRRSIPREDI
Cyanothece sp. PCC 8801 241 SKVLEIHMELMDEFSQQLRLEGRSEEILLDYRLTIIDILAHLGEMYRRSIPREDI
Cyanothece sp. PCC 8802 241 SKVLEIHMELMDEFSQQLRLEGRSEEILLDYRLTIIDILAHLGEMYRRSIPREDI
Cylindrospermopsis r. CS-505 149 PQVIEIHMELIDEFSKQLRLEGRSDETLLDYRLTLIDVLANLCEVYRCSTSRIT.
Fischerella sp. JSC-11 65 PQIIEIHMELIDEFSKQLRLEGRSDETLLDYRLTLIDILAHLCELYRRSLLK...
Leptolyngbya b. IAM M-101 234 AQIVEIHMELMDEFAKQLRLEGRNEEVLLDYRLTLIDTIAHLCEMYRRSIPRES.
Lyngbya sp. PCC 8106 225 TRIVEIHMELMDNFSKQLRLEGRSEDVLLDYRLTLIDMVAHLCEMYRRSIPRES.
Microcoleus c. PCC 7420 252 SKIVEIHMDLMDIFAQQLRLEGRSEDILTDYRLTLIDVIAHLGEMYRRSIPREL.
Microcoleus v. FGP-2 253 TQIVEIHMELMDEFAKQLKIEGRSEEILLDYRLTLIDAIAHNCEMYRRSIPKETS
Microcystis a. NIES-843 227 SQILEIHMELMDEFSQQLRLEGRSEEILLDYRLALIDILAHLGEMYRRSIPREDI
Microcystis a. PCC 7806 242 SQILEIHMELMDEFSQQLRLEGRSEEILLDYRLALIDILAHLGEMYRRSIPREDI
Microcystis a. PCC 7820 227 SQILEIHMELMDEFSQQLRLEGRSEEILLDYRLALIDILAHLGEMYRRSIPREDI
Microcystis a. PCC 7941 273 SQILEIHIELMDEFSQQLRLEGRSEEILLDYRLALIDILAHLGEMYRRSIPREDI
Microcystis a. PCC 9443 246 SQILEIHMELMDEFSQQLRLEGRSEEILLDYRLALIDILAHLGEMYRRSIPREDI
Microcystis a. PCC 9701 246 SQILEIHIRLMDEFSQQLRLEGRSEEILLDYRLALIDILAHLGEMYRRSIPREDI
Microcystis a. PCC 9717 246 SQILEIHMELMDEFSQQLRLEGRSEEILLDYRLALIDILAHLGEMYRRSIPREDI
Microcystis a. PCC 9806 274 SQILEIHMELMDEFSQQLRLEGRSEEILLDYRLALIDILAHLGEMYRRSIPREDI
Microcystis a. PCC 9807 246 SQILEIHMELMDEFSQQLRLEGRSEEILLDYRLALIDILAHLGEMYRRSIPREDI
Microcystis sp. T1-4 246 SQILEIHMELMDEFSQQLRLEGRSEEILLDYRLALIDILAHLGEMYRRSIPREDI
Moorea p. 3L 253 SKIVEIHMELMDEFSKQLQLEGRREDILLDYRLTLIDVIAHLGEMYRRSIPREL.
Nodularia s. CCY9414 50 PQIIEFHMELIEDFSIQLRLEGRSDEALLDYRLALIDILAHLCELYRCSISK...
Nostoc a. 0708 145 PQIIEIHMELIDEFSKQLRLEGRSDETLLDYRLTLIDILAHLCEVYRVSSSKIN.
Nostoc c. 142 PQIIEIHMELIDEFSIQLRLEGRTNETLLDYRLTLIDVLAHLCEAYRCAISK...
Nostoc p. PCC 73102 142 PQIIEIHMEVIEEFSIQLRLEGRSNETLLDYRLTLIDILAHLCEVYRSSISK...
Nostoc sp. PCC 7120 51 PEIIEIHMELIDEFSKQLRLEGRGDETLMDYRLTLIDILAHLCEAYRGAIFK...
Nostoc sp. PCC 9709 142 PQIIEIHMEVIEDFSNQLRLEGRSNETLLDYRLTLIDILAHLCELYRSSISK...
Oscillatoria sp. PCC 6506 273 TQIVEIHMELMDEFANQLRLEGRSEEVLLDYRLTLIDAIAHLCEMYRRSIPRDS.
Raphidiopsis b. D9 50 PQVIEIHMELIDEFSKQLRLEGRSDETLLDYRLTLIDVLANLCEVYRCSTSKIN.
Synechococcus e. PCC 6301 231 TKVVEIHMELMDEFAKKLRVEGRSEDILLDYRLTLIDVIAHLCEMYRRSIPRET.
Synechococcus sp. PCC 7335 249 PKLVEIHMELIEDFSKQLRLEGRSDEVLIDYRLTLIDVIAHLCEMYRRSIPREP.
Synechococcus sp. BL107 215 TRTVEIHVNLIDEYWKKLRLEGHKNDFLQDYRLALIDVMAHLCEMYRRSIPPDIP
Synechococcus sp. CB0101 218 NKVVEIHVDLIDAFWKQLRLEGHKNDFLQDYRLALIDVMAHLCEMYRRSVPPDLP
Synechococcus sp. CB0205 216 NSVVEIHVDLMDSFWKQLRLEGHKNDFLQDYRLALIDVMAHLCEMYRRSIPPDLP
Synechococcus sp. CC9311 256 TQTVEIHMNLIDEFWKQLRLEGHKDDFLQDYRLALIDVMAHLCEMYRRSVPGDIP
Synechococcus sp. CC9605 217 TRTVEIHVDLIDEFWKQLSFEGHKHDFLQDYRLALIDVMAHLCEMYRRSIPPDLP
Synechococcus sp. CC9902 215 TRTVEIHVNLIDEYWKKLRLEGHKNDFLQDYRLALIDVMAHLCEMYRRSIPPDIP
Synechococcus sp. JA-2-3B 277 AQLLELHMNLMEDFAKQLRLEGRSEEILLDYRLTLIDVMAHLSEMYRRSIPKPAP
Synechococcus sp. JA-3-3Ab 247 AQLLELHMNLMEDFAKQLRLEGRSEEILLDYRLTLIDVMAHLSEMYRRSIPKSAP
Synechococcus sp. PCC 7002 236 SYILETHMKLMDEFAQQLRLEGRSEEILLDYRLTLIDVIAHLCEMYRRSIPREDL
Synechococcus sp. RCC307 219 TRAVEIHMKLIDGWSKQLALEGHKSDFLQDYRLALIDVMAHLCEMYRRSIPPESV
Synechococcus sp. RS9916 226 TRAVEIHMNLIDEFWKKLRLEGHKNDFLQDYRLALIDVMAHLCEMYRRSIPTESQ
Synechococcus sp. RS9917 226 TRAVEIHMNLIDLFWKQLRLEGHKNDFLQDYRLALIDVMAHLCEMYRRSIPPDLS
Synechococcus sp. WH 5701 222 TKTVEIHVDMIDEFWKKLQLEGHKQEFLKDYRLALIDVMAHLCEMYRRSLPPEVT
Synechococcus sp. WH 7803 223 TRTVEIHMNLIDEFWKQLRLEGHKNDFLQDYRLALIDVMAHLCEMYRRSIPPEVA
Synechococcus sp. WH 7805 224 TRTVEIHMNLIDEFWKQLRLEGHKNDFLQDYRLALIDVMAHLCEMYRRSIPPEVA
Synechococcus sp. WH 8016 226 TQTVEIHMNLIDIFWKQLRLEGHKDDFLQDYRLALIDVMAHLCEMYRRSMPGDIP
Synechococcus sp. WH 8102 219 TRTVDIHVDQIDEFWKQLRLEGNKSEFLQDYRLALIDVMAHLCEMYRRSIPPDIP
Synechococcus sp. WH 8109 217 TRTVEIHVDLNR...
Synechocystis sp. PCC 6803 236 SQILEIHMELMDEFSQHLRLEGRSEEVLLDYRLVLIDILAHLGEMYRRSIPREDI
Thermosynechococcus e. BP-1 230 SQVLEIHVELMDTFSKQLRLEGRSEDILLDYRLTLIDVIAHLGEMYRRSIPREV.
Trichodesmium e. IMS101 272 TQVVEIHMELMDEFAIQLRLEGRSEEMLLDYRLTLIDTIAHLCEMYRRSIPRES.
consensus ****!******* ******* * * **** ******** ********
logo

Synechococcus e. PCC 7942	285	....
Acaryochloris m. MBIC11017	300	....
Acaryochloris sp. CCMEE 5410	300	....
Anabaena v. ATCC 29413	90	....
Arthrospira m. CS-328	284	GE.....................
Arthrospira m. FACHB-438	284	GE.....................
Arthrospira p. str. Paraca	284	GE.....................
Crocosphaera w. WH 0003	274	LFDLLNQMD..............
Crocosphaera w. WH 8501	301	LFDLLNQMD..............
Cyanobium sp. PCC 7001	293	LIRFSAST...............
Cyanothece sp. ATCC 51142	290	LFDLLNQID..............
Cyanothece sp. CCY0110	274	LFDLLNQID..............
Cyanothece sp. PCC 7424	307	PYELLFRID..............
Cyanothece sp. PCC 7425	292	SRET...................
Cyanothece sp. PCC 7822	312	PYELLFRID..............
Cyanothece sp. PCC 8801	296	LFDLLYQID..............
Cyanothece sp. PCC 8802	296	LFDLLYQID..............
Cylindrospermopsis r. CS-505	203	....
Fischerella sp. JSC-11	117	....
Leptolyngbya b. IAM M-101	288	....
Lyngbya sp. PCC 8106	279	....
Microcoleus c. PCC 7420	306	....
Microcoleus v. FGP-2	308	QGRM...................
Microcystis a. NIES-843	282	PYDLLLGID..............
Microcystis a. PCC 7806	297	PYDLLLGID..............
Microcystis a. PCC 7820	282	PYDLLLGID..............
Microcystis a. PCC 7941	328	PYDLSQMSG..............
Microcystis a. PCC 9443	301	PYDLLLGID..............
Microcystis a. PCC 9701	301	PYDLLLGID..............
Microcystis a. PCC 9717	301	PYDLLLGID..............
Microcystis a. PCC 9806	329	PYDLSQMSG..............
Microcystis a. PCC 9807	301	PYDLLLGID..............
Microcystis sp. T1-4	301	PDDLSQMSG..............
Moorea p. 3L	307	....
Nodularia s. CCY9414	102	....
Nostoc a. 0708	199	....
Nostoc c.	194	....
Nostoc p. PCC 73102	194	....
Nostoc sp. PCC 7120	103	....
Nostoc sp. PCC 9709	194	....
Oscillatoria sp. PCC 6506	327	....
Raphidiopsis b. D9	104	....
Synechococcus e. PCC 6301	285	....
Synechococcus sp. PCC 7335	303	....
Synechococcus sp. BL107	270	LPGEASNRPSVAVDQPMSSEELL
Synechococcus sp. CB0101	273	LGAPQDPEEASQSPSQEVI....
Synechococcus sp. CB0205	271	LAEPPPGESVG............
Synechococcus sp. CC9311	311	LVPAGSQRRQLQNSEVSL.....
Synechococcus sp. CC9605	272	LSGTASSRVRRPMDQLDASEESS
Synechococcus sp. CC9902	270	LPSEASNRLSVAVDQPMSSEELL
Synechococcus sp. JA-2-3B	332	RDP....................
Synechococcus sp. JA-3-3Ab	302	RDP....................
Synechococcus sp. PCC 7002	291	PFELLFRID..............
Synechococcus sp. RCC307	274	GAGARASAGSAVAQQEESPS...
Synechococcus sp. RS9916	281	LARSEQFGEEVVSS.........
Synechococcus sp. RS9917	281	LSASVASQPAGASAVTDQELSS.
Synechococcus sp. WH 5701	277	DKPSEGMVAKIITASQVQS....
Synechococcus sp. WH 7803	278	LAVPSEQRRSLMDSEVSS.....
Synechococcus sp. WH 7805	279	LATSAEYRQALMDSQVSP.....
Synechococcus sp. WH 8016	281	LVPSGSQRRQLQDSEVTL.....
Synechococcus sp. WH 8102	274	LSGLASGRHRREADLPDAPEVSS
Synechococcus sp. WH 8109	229	....
Synechocystis sp. PCC 6803	291	PFDVYYQTD..............
Thermosynechococcus e. BP-1	284	....
Trichodesmium e. IMS101	326	....
consensus		

logo

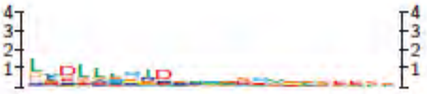

(B)

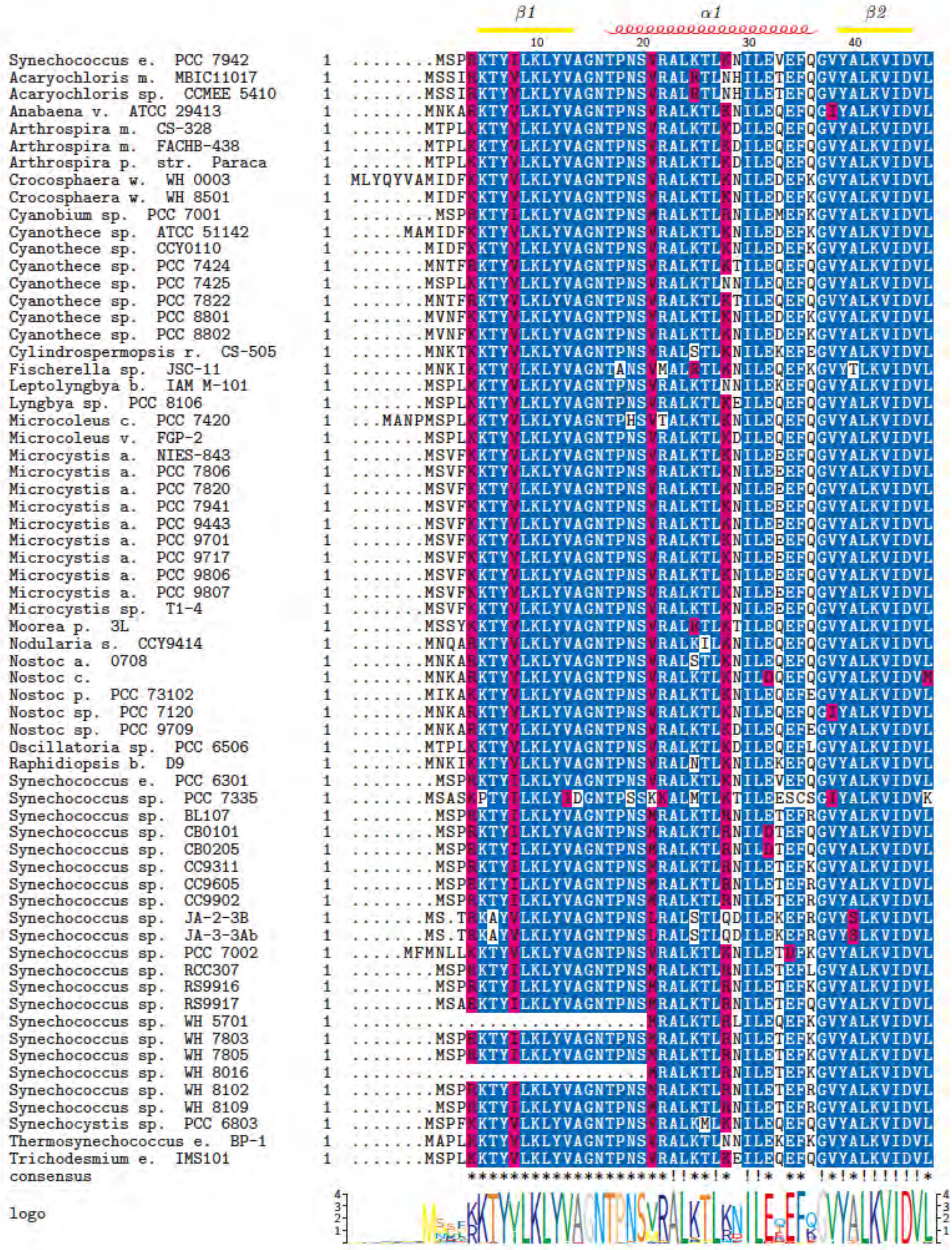
β1
α1
β2
10
20
30
40
Synechococcus e. PCC 7942
Acaryochloris m. MBIC11017
Acaryochloris sp. CCMEE 5410
Anabaena v. ATCC 29413
Arthrospira m. CS-328
Arthrospira m. FACHB-438
Arthrospira p. str. Paraca
Crocosphaera w. WH 0003
Crocosphaera w. WH 8501
Cyanobium sp. PCC 7001
Cyanothece sp. ATCC 51142
Cyanothece sp. CCY0110
Cyanothece sp. PCC 7424
Cyanothece sp. PCC 7425
Cyanothece sp. PCC 7822
Cyanothece sp. PCC 8801
Cyanothece sp. PCC 8802
Cylindrospermopsis r. CS-505
Fischerella sp. JSC-11
Leptolyngbya b. IAM M-101
Lyngbya sp. PCC 8106
Microcoleus c. PCC 7420
Microcoleus v. FGP-2
Microcystis a. NIES-843
Microcystis a. PCC 7806
Microcystis a. PCC 7820
Microcystis a. PCC 7941
Microcystis a. PCC 9443
Microcystis a. PCC 9701
Microcystis a. PCC 9717
Microcystis a. PCC 9806
Microcystis a. PCC 9807
Microcystis sp. T1-4
Moorea p. 3L
Nodularia s. CCY9414
Nostoc a. 0708
Nostoc c.
Nostoc p. PCC 73102
Nostoc sp. PCC 7120
Nostoc sp. PCC 9709
Oscillatoria sp. PCC 6506
Raphidiopsis b. D9
Synechococcus e. PCC 6301
Synechococcus sp. PCC 7335
Synechococcus sp. BL107
Synechococcus sp. CB0101
Synechococcus sp. CB0205
Synechococcus sp. CC9311
Synechococcus sp. CC9605
Synechococcus sp. CC9902
Synechococcus sp. JA-2-3B
Synechococcus sp. JA-3-3Ab
Synechococcus sp. PCC 7002
Synechococcus sp. RCC307
Synechococcus sp. RS9916
Synechococcus sp. RS9917
Synechococcus sp. WH 5701
Synechococcus sp. WH 7803
Synechococcus sp. WH 7805
Synechococcus sp. WH 8016
Synechococcus sp. WH 8102
Synechococcus sp. WH 8109
Synechocystis sp. PCC 6803
Thermosynechococcus e. BP-1
Trichodesmium e. IMS101
consensus
logo

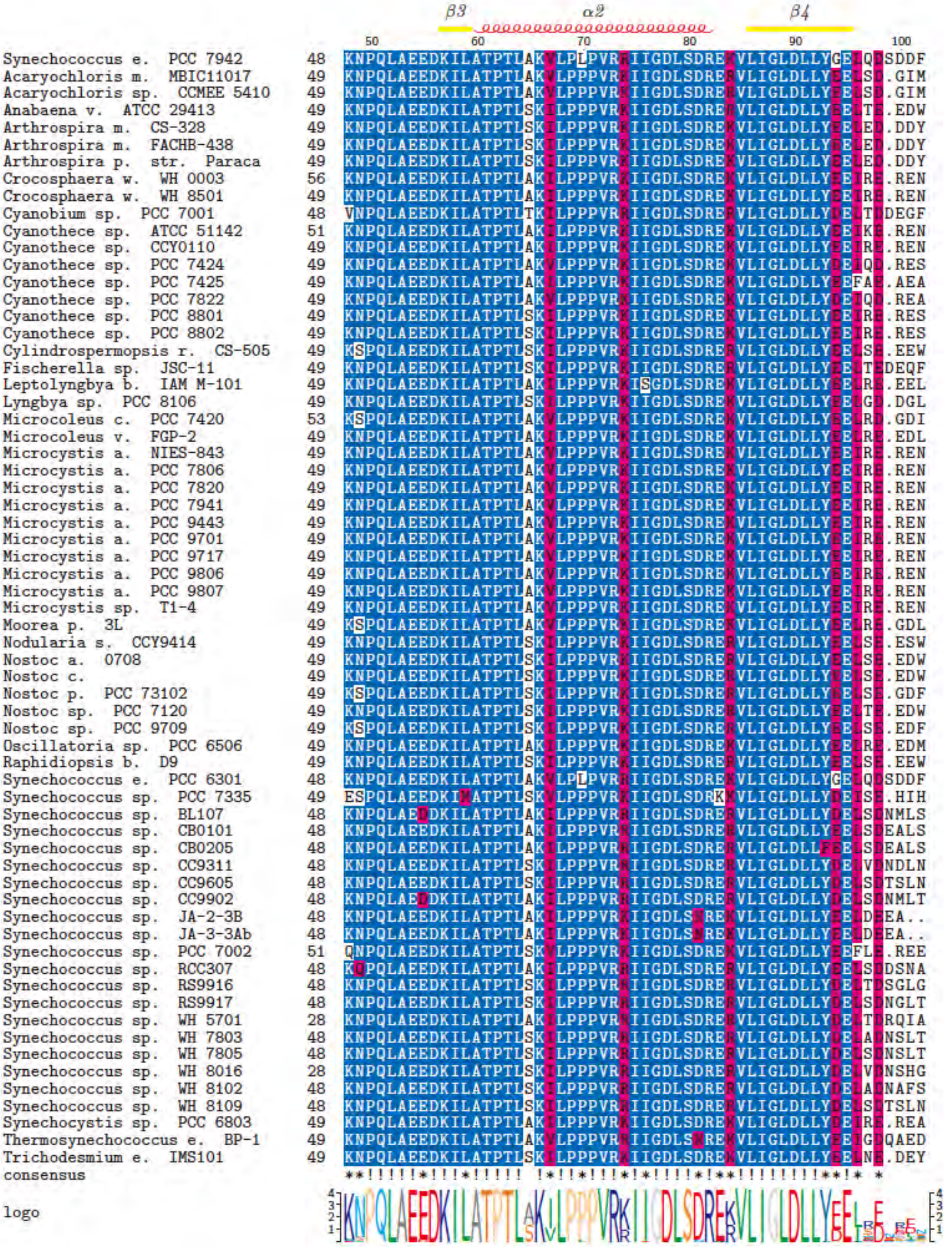

β3
α2
β4
50
60
70
80
90
100
Synechococcus e. PCC 7942
Acaryochloris m. MBIC11017
Acaryochloris sp. CCMEE 5410
Anabaena v. ATCC 29413
Arthrospira m. CS-328
Arthrospira m. FACHB-438
Arthrospira p. str. Paraca
Crocosphaera w. WH 0003
Crocosphaera w. WH 8501
Cyanobium sp. PCC 7001
Cyanothece sp. ATCC 51142
Cyanothece sp. CCY0110
Cyanothece sp. PCC 7424
Cyanothece sp. PCC 7425
Cyanothece sp. PCC 7822
Cyanothece sp. PCC 8801
Cyanothece sp. PCC 8802
Cylindrospermopsis r. CS-505
Fischerella sp. JSC-11
Leptolyngbya b. IAM M-101
Lyngbya sp. PCC 8106
Microcoleus c. PCC 7420
Microcoleus v. FGP-2
Microcystis a. NIES-843
Microcystis a. PCC 7806
Microcystis a. PCC 7820
Microcystis a. PCC 7941
Microcystis a. PCC 9443
Microcystis a. PCC 9701
Microcystis a. PCC 9717
Microcystis a. PCC 9806
Microcystis a. PCC 9807
Microcystis sp. T1-4
Moorea p. 3L
Nodularia s. CCY9414
Nostoc a. 0708
Nostoc c.
Nostoc p. PCC 73102
Nostoc sp. PCC 7120
Nostoc sp. PCC 9709
Oscillatoria sp. PCC 6506
Raphidiopsis b. D9
Synechococcus e. PCC 6301
Synechococcus sp. PCC 7335
Synechococcus sp. BL107
Synechococcus sp. CB0101
Synechococcus sp. CB0205
Synechococcus sp. CC9311
Synechococcus sp. CC9605
Synechococcus sp. CC9902
Synechococcus sp. JA-2-3B
Synechococcus sp. JA-3-3Ab
Synechococcus sp. PCC 7002
Synechococcus sp. RCC307
Synechococcus sp. RS9916
Synechococcus sp. RS9917
Synechococcus sp. WH 5701
Synechococcus sp. WH 7803
Synechococcus sp. WH 7805
Synechococcus sp. WH 8016
Synechococcus sp. WH 8102
Synechococcus sp. WH 8109
Synechocystis sp. PCC 6803
Thermosynechococcus e. BP-1
Trichodesmium e. IMS101
consensus
logo

Synechococcus e. PCC 7942	103	....
Acaryochloris m. MBIC11017	103	EY................................
Acaryochloris sp. CCMEE 5410	103	EY................................
Anabaena v. ATCC 29413	103	EAQSSL............................
Arthrospira m. CS-328	103	NF................................
Arthrospira m. FACHB-438	103	NF................................
Arthrospira p. str. Paraca	103	NF................................
Crocosphaera w. WH 0003	110	DP................................
Crocosphaera w. WH 8501	103	DP................................
Cyanobium sp. PCC 7001	103	DALPLASPASEPDASLPAVPSEGARSVDGLNPPA
Cyanothece sp. ATCC 51142	105	DP................................
Cyanothece sp. CCY0110	103	DP................................
Cyanothece sp. PCC 7424	103	ELDF..............................
Cyanothece sp. PCC 7425	103	EAE...............................
Cyanothece sp. PCC 7822	103	ELDF..............................
Cyanothece sp. PCC 8801	103	EL................................
Cyanothece sp. PCC 8802	103	EL................................
Cylindrospermopsis r. CS-505	103	DNIDDHKIEEIN......................
Fischerella sp. JSC-11	104	QTE...............................
Leptolyngbya b. IAM M-101	103	DSQ...............................
Lyngbya sp. PCC 8106	103	NL................................
Microcoleus c. PCC 7420	107	DF................................
Microcoleus v. FGP-2	103	NS................................
Microcystis a. NIES-843	103	DS................................
Microcystis a. PCC 7806	103	DS................................
Microcystis a. PCC 7820	103	DS................................
Microcystis a. PCC 7941	103	DS................................
Microcystis a. PCC 9443	103	DS................................
Microcystis a. PCC 9701	103	DG................................
Microcystis a. PCC 9717	103	DS................................
Microcystis a. PCC 9806	103	DS................................
Microcystis a. PCC 9807	103	DS................................
Microcystis sp. T1-4	103	DS................................
Moorea p. 3L	103	DF................................
Nodularia s. CCY9414	103	EE................................
Nostoc a. 0708	103	E.................................
Nostoc c.	103	EESHNL............................
Nostoc p. PCC 73102	103	EE................................
Nostoc sp. PCC 7120	103	EAQSNL............................
Nostoc sp. PCC 9709	103	E.................................
Oscillatoria sp. PCC 6506	103	NLHDRI............................
Raphidiopsis b. D9	103	DNIDDHKIEEIN......................
Synechococcus e. PCC 6301	103	....
Synechococcus sp. PCC 7335	103	N.................................
Synechococcus sp. BL107	103	SSFLDDSDEESAPATSDS................
Synechococcus sp. CB0101	103	EPYSEEEDSGGLL.....................
Synechococcus sp. CB0205	103	ESLLDQDAVGDLV.....................
Synechococcus sp. CC9311	103	SSLMDALGVPDIEEADS.................
Synechococcus sp. CC9605	103	SSLIDAVDEETDTTISSDP...............
Synechococcus sp. CC9902	103	SSFLDDSDEESAPATSDS................
Synechococcus sp. JA-2-3B	101	....
Synechococcus sp. JA-3-3Ab	101	....
Synechococcus sp. PCC 7002	105	EL................................
Synechococcus sp. RCC307	103	QVLDDFSE..........................
Synechococcus sp. RS9916	103	SSFRDSLSDEDMVSTDS.................
Synechococcus sp. RS9917	103	SSLMDALEDADTDSTDS.................
Synechococcus sp. WH 5701	83	DEMMD..ESDEI......................
Synechococcus sp. WH 7803	103	STLMDALEDADTTDSDS.................
Synechococcus sp. WH 7805	103	STLMDALEDADTTDSDS.................
Synechococcus sp. WH 8016	83	SSLMDALEDPDIAEADS.................
Synechococcus sp. WH 8102	103	SG................................
Synechococcus sp. WH 8109	103	SSLIDAVDEETDTTISSDP...............
Synechocystis sp. PCC 6803	103	EDQ...............................
Thermosynechococcus e. BP-1	104	DLGLE.............................
Trichodesmium e. IMS101	103	NL................................
consensus		

logo

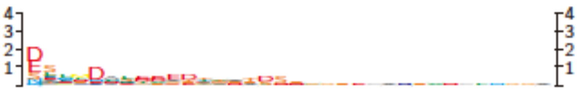

(C)

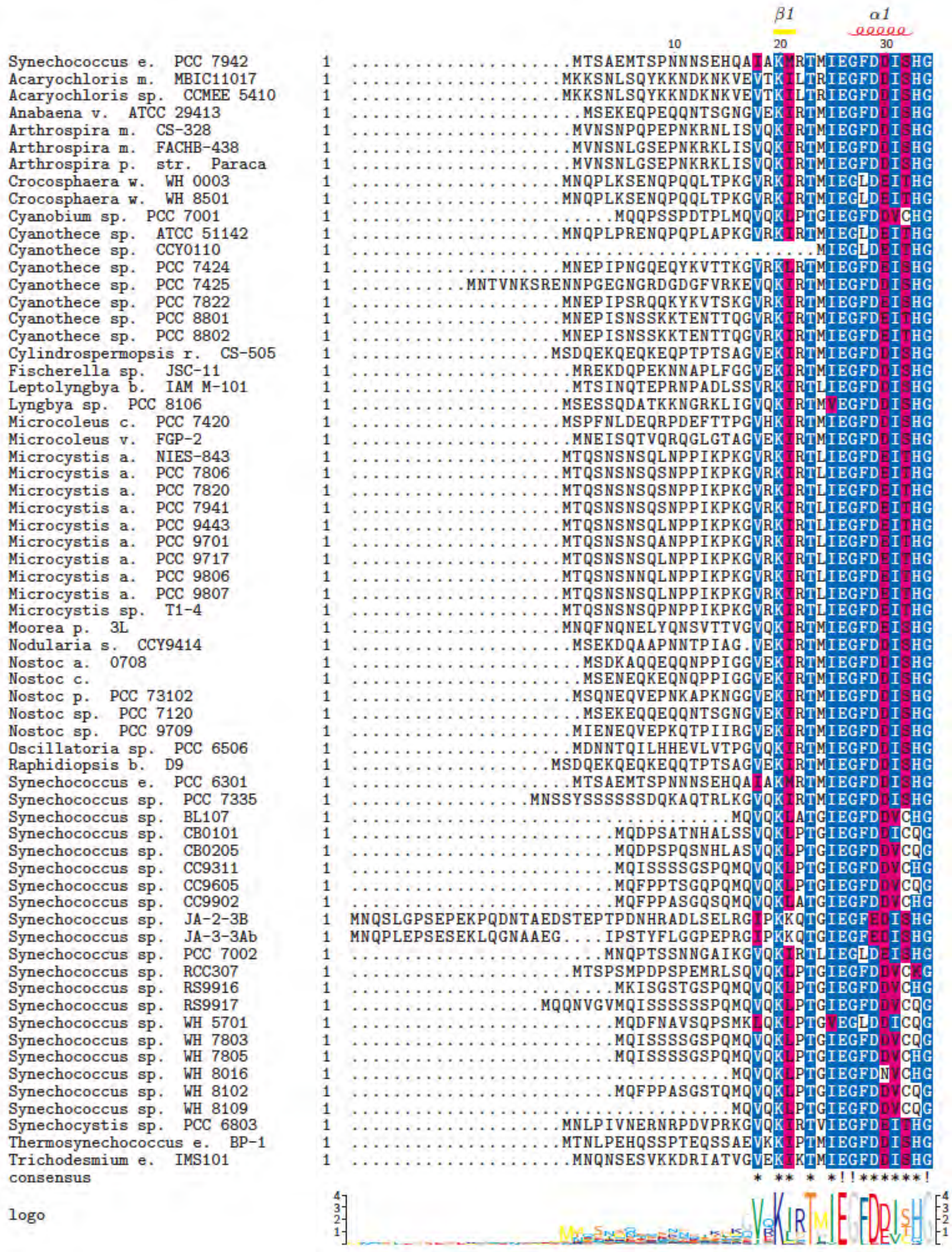

β1
α1
10
20
30
Synechococcus e. PCC 7942
Acaryochloris m. MBIC11017
Acaryochloris sp. CCMEE 5410
Anabaena v. ATCC 29413
Arthrospira m. CS-328
Arthrospira m. FACHB-438
Arthrospira p. str. Paraca
Crocosphaera w. WH 0003
Crocosphaera w. WH 8501
Cyanobium sp. PCC 7001
Cyanothece sp. ATCC 51142
Cyanothece sp. CCY0110
Cyanothece sp. PCC 7424
Cyanothece sp. PCC 7425
Cyanothece sp. PCC 7822
Cyanothece sp. PCC 8801
Cyanothece sp. PCC 8802
Cylindrospermopsis r. CS-505
Fischerella sp. JSC-11
Leptolyngbya b. IAM M-101
Lyngbya sp. PCC 8106
Microcoleus c. PCC 7420
Microcoleus v. FGP-2
Microcystis a. NIES-843
Microcystis a. PCC 7806
Microcystis a. PCC 7820
Microcystis a. PCC 7941
Microcystis a. PCC 9443
Microcystis a. PCC 9701
Microcystis a. PCC 9717
Microcystis a. PCC 9806
Microcystis a. PCC 9807
Microcystis sp. T1-4
Moorea p. 3L
Nodularia s. CCY9414
Nostoc a. 0708
Nostoc c.
Nostoc p. PCC 73102
Nostoc sp. PCC 7120
Nostoc sp. PCC 9709
Oscillatoria sp. PCC 6506
Raphidiopsis b. D9
Synechococcus e. PCC 6301
Synechococcus sp. PCC 7335
Synechococcus sp. BL107
Synechococcus sp. CB0101
Synechococcus sp. CB0205
Synechococcus sp. CC9311
Synechococcus sp. CC9605
Synechococcus sp. CC9902
Synechococcus sp. JA-2-3B
Synechococcus sp. JA-3-3Ab
Synechococcus sp. PCC 7002
Synechococcus sp. RCC307
Synechococcus sp. RS9916
Synechococcus sp. RS9917
Synechococcus sp. WH 5701
Synechococcus sp. WH 7803
Synechococcus sp. WH 7805
Synechococcus sp. WH 8016
Synechococcus sp. WH 8102
Synechococcus sp. WH 8109
Synechocystis sp. PCC 6803
Thermosynechococcus e. BP-1
Trichodesmium e. IMS101
consensus
logo

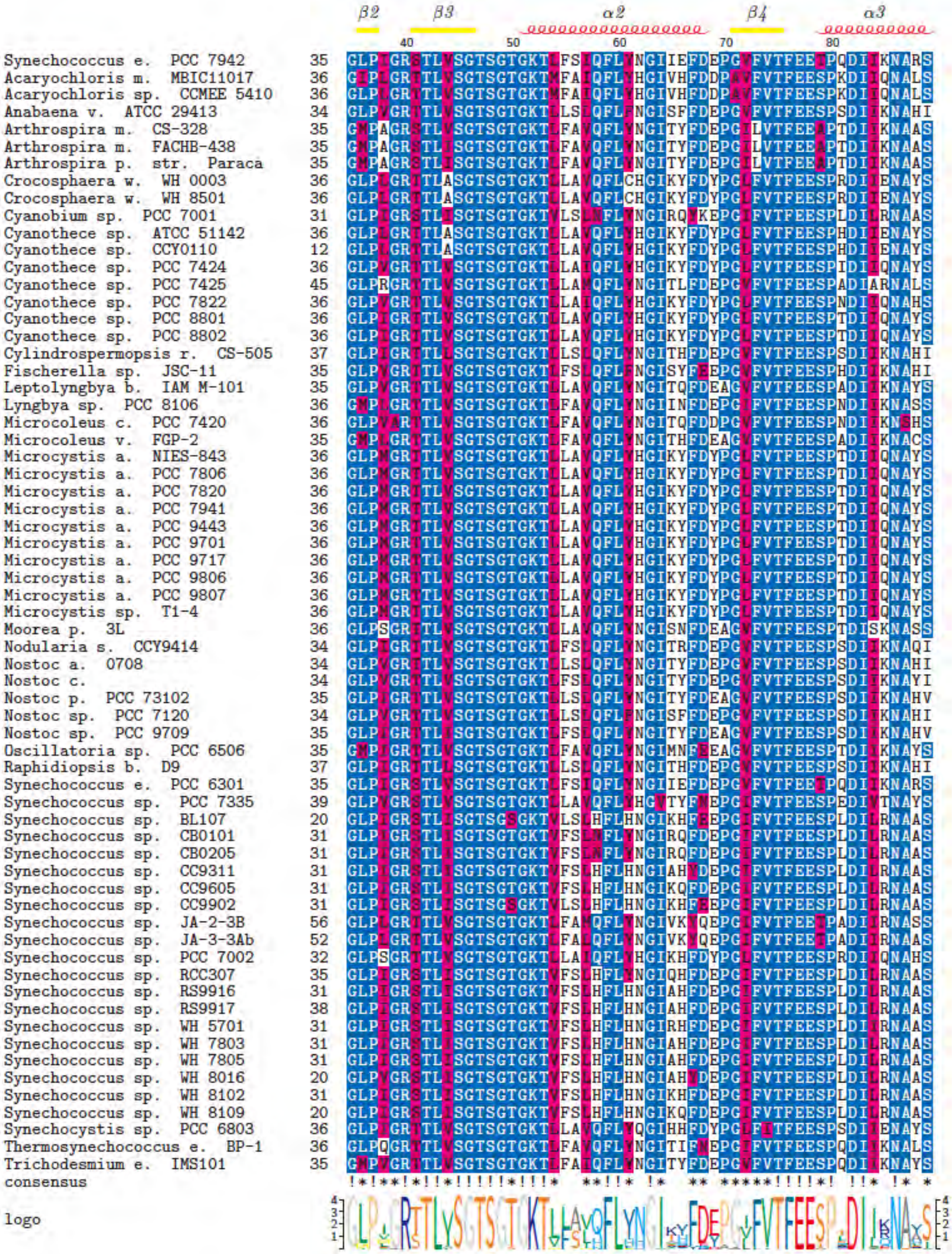
β2
β3
α2
β4
α3
40
50
60
70
80
Synechococcus e. PCC 7942 35
Acaryochloris m. MBIC11017 36
Acaryochloris sp. CCMEE 5410 36
Anabaena v. ATCC 29413 34
Arthrospira m. CS-328 35
Arthrospira m. FACHB-438 35
Arthrospira p. str. Paraca 35
Crocosphaera w. WH 0003 36
Crocosphaera w. WH 8501 36
Cyanobium sp. PCC 7001 31
Cyanothece sp. ATCC 51142 36
Cyanothece sp. CCY0110 12
Cyanothece sp. PCC 7424 36
Cyanothece sp. PCC 7425 45
Cyanothece sp. PCC 7822 36
Cyanothece sp. PCC 8801 36
Cyanothece sp. PCC 8802 36
Cylindrospermopsis r. CS-505 37
Fischerella sp. JSC-11 35
Leptolyngbya b. IAM M-101 35
Lyngbya sp. PCC 8106 36
Microcoleus c. PCC 7420 36
Microcoleus v. FGP-2 35
Microcystis a. NIES-843 36
Microcystis a. PCC 7806 36
Microcystis a. PCC 7820 36
Microcystis a. PCC 7941 36
Microcystis a. PCC 9443 36
Microcystis a. PCC 9701 36
Microcystis a. PCC 9717 36
Microcystis a. PCC 9806 36
Microcystis a. PCC 9807 36
Microcystis sp. T1-4 36
Moorea p. 3L 36
Nodularia s. CCY9414 34
Nostoc a. 0708 34
Nostoc c. 34
Nostoc p. PCC 73102 35
Nostoc sp. PCC 7120 34
Nostoc sp. PCC 9709 35
Oscillatoria sp. PCC 6506 35
Raphidiopsis b. D9 37
Synechococcus e. PCC 6301 35
Synechococcus sp. PCC 7335 39
Synechococcus sp. BL107 20
Synechococcus sp. CB0101 31
Synechococcus sp. CB0205 31
Synechococcus sp. CC9311 31
Synechococcus sp. CC9605 31
Synechococcus sp. CC9902 31
Synechococcus sp. JA-2-3B 56
Synechococcus sp. JA-3-3Ab 52
Synechococcus sp. PCC 7002 32
Synechococcus sp. RCC307 35
Synechococcus sp. RS9916 31
Synechococcus sp. RS9917 38
Synechococcus sp. WH 5701 31
Synechococcus sp. WH 7803 31
Synechococcus sp. WH 7805 31
Synechococcus sp. WH 8016 20
Synechococcus sp. WH 8102 31
Synechococcus sp. WH 8109 20
Synechocystis sp. PCC 6803 36
Thermosynechococcus e. BP-1 36
Trichodesmium e. IMS101 35
consensus
logo

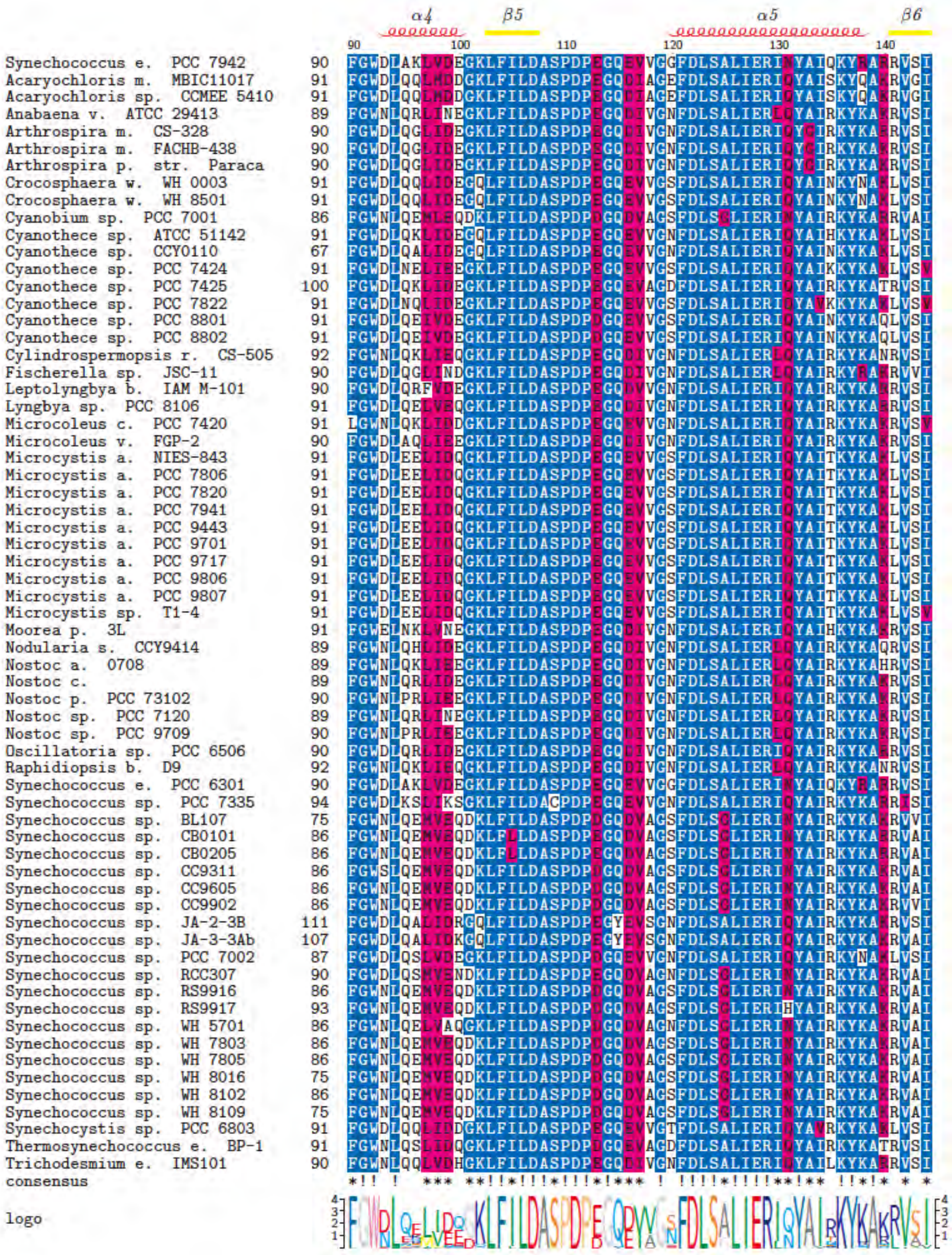
α4
β5
α5
β6
consensus
logo

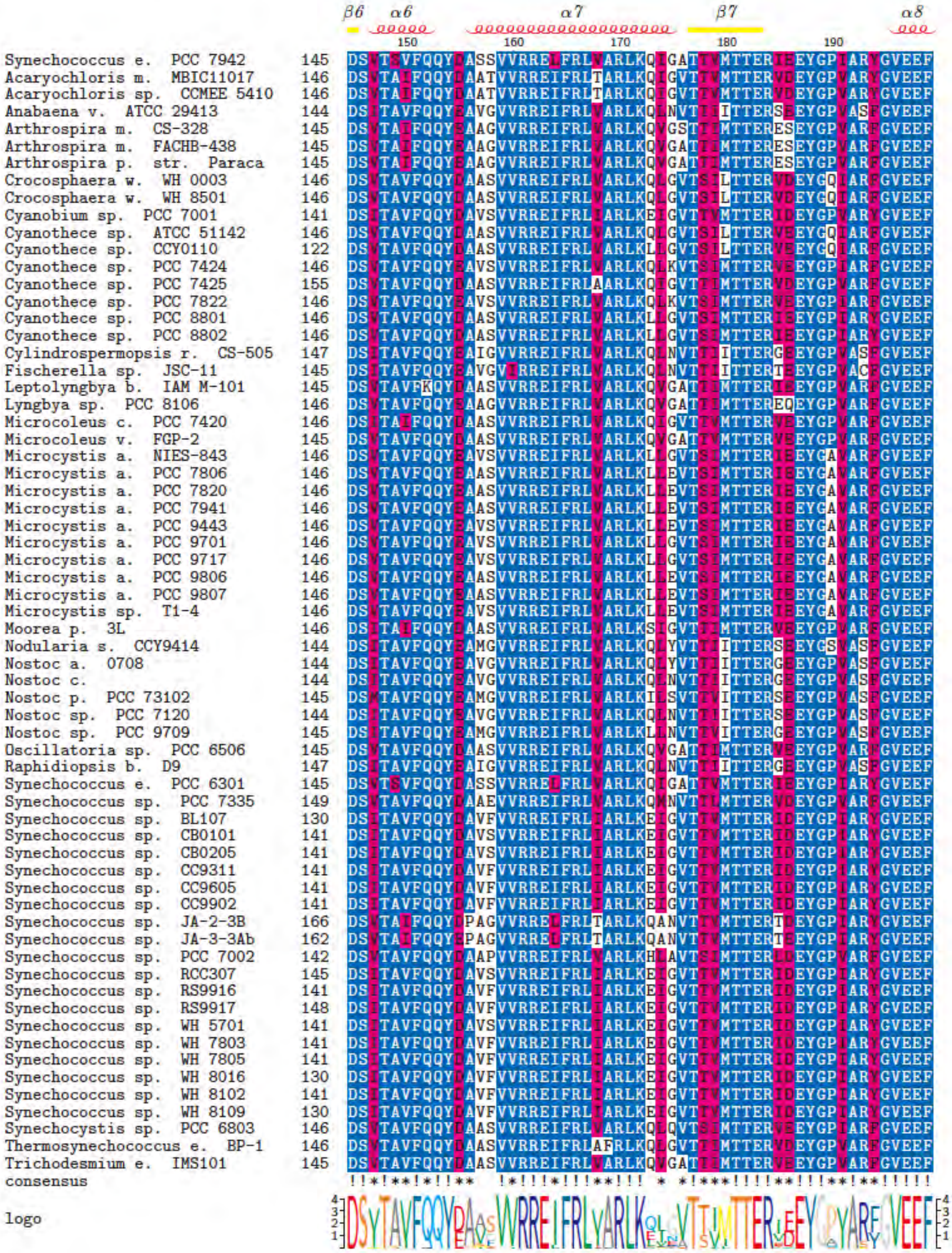

β6 α6 α7 β7 α8
150 160 170 180 190
Synechococcus e. PCC 7942 145
Acaryochloris m. MBIC11017 146
Acaryochloris sp. CCMEE 5410 146
Anabaena v. ATCC 29413 144
Arthrospira m. CS-328 145
Arthrospira m. FACHB-438 145
Arthrospira p. str. Paraca 145
Crocosphaera w. WH 0003 146
Crocosphaera w. WH 8501 146
Cyanobium sp. PCC 7001 141
Cyanothece sp. ATCC 51142 146
Cyanothece sp. CCY0110 122
Cyanothece sp. PCC 7424 146
Cyanothece sp. PCC 7425 155
Cyanothece sp. PCC 7822 146
Cyanothece sp. PCC 8801 146
Cyanothece sp. PCC 8802 146
Cylindrospermopsis r. CS-505 147
Fischerella sp. JSC-11 145
Leptolyngbya b. IAM M-101 145
Lyngbya sp. PCC 8106 146
Microcoleus c. PCC 7420 146
Microcoleus v. FGP-2 145
Microcystis a. NIES-843 146
Microcystis a. PCC 7806 146
Microcystis a. PCC 7820 146
Microcystis a. PCC 7941 146
Microcystis a. PCC 9443 146
Microcystis a. PCC 9701 146
Microcystis a. PCC 9717 146
Microcystis a. PCC 9806 146
Microcystis a. PCC 9807 146
Microcystis sp. T1-4 146
Moorea p. 3L 146
Nodularia s. CCY9414 144
Nostoc a. 0708 144
Nostoc c. 144
Nostoc p. PCC 73102 145
Nostoc sp. PCC 7120 144
Nostoc sp. PCC 9709 145
Oscillatoria sp. PCC 6506 145
Raphidiopsis b. D9 147
Synechococcus e. PCC 6301 145
Synechococcus sp. PCC 7335 149
Synechococcus sp. BL107 130
Synechococcus sp. CB0101 141
Synechococcus sp. CB0205 141
Synechococcus sp. CC9311 141
Synechococcus sp. CC9605 141
Synechococcus sp. CC9902 141
Synechococcus sp. JA-2-3B 166
Synechococcus sp. JA-3-3Ab 162
Synechococcus sp. PCC 7002 142
Synechococcus sp. RCC307 145
Synechococcus sp. RS9916 141
Synechococcus sp. RS9917 148
Synechococcus sp. WH 5701 141
Synechococcus sp. WH 7803 141
Synechococcus sp. WH 7805 141
Synechococcus sp. WH 8016 130
Synechococcus sp. WH 8102 141
Synechococcus sp. WH 8109 130
Synechocystis sp. PCC 6803 146
Thermosynechococcus e. BP-1 146
Trichodesmium e. IMS101 145
consensus
logo

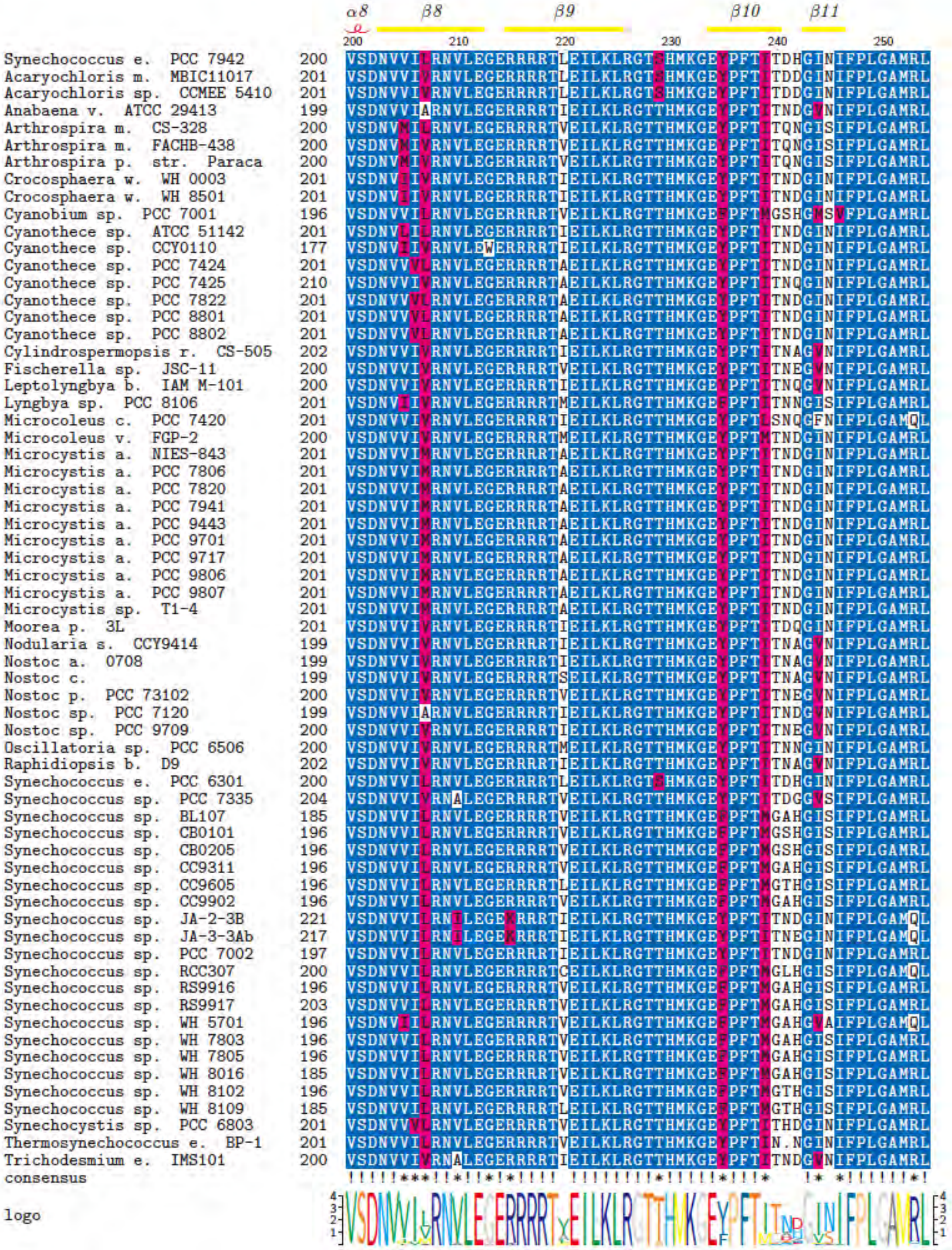
α8
β8
β9
β10
β11
200
210
220
230
240
250
Synechococcus e. PCC 7942 200
Acaryochloris m. MBIC11017 201
Acaryochloris sp. CCMEE 5410 201
Anabaena v. ATCC 29413 199
Arthrospira m. CS-328 200
Arthrospira m. FACHB-438 200
Arthrospira p. str. Paraca 200
Crocosphaera w. WH 0003 201
Crocosphaera w. WH 8501 201
Cyanobium sp. PCC 7001 196
Cyanothece sp. ATCC 51142 201
Cyanothece sp. CCY0110 177
Cyanothece sp. PCC 7424 201
Cyanothece sp. PCC 7425 210
Cyanothece sp. PCC 7822 201
Cyanothece sp. PCC 8801 201
Cyanothece sp. PCC 8802 201
Cylindrospermopsis r. CS-505 202
Fischerella sp. JSC-11 200
Leptolyngbya b. IAM M-101 200
Lyngbya sp. PCC 8106 201
Microcoleus c. PCC 7420 201
Microcoleus v. FGP-2 200
Microcystis a. NIES-843 201
Microcystis a. PCC 7806 201
Microcystis a. PCC 7820 201
Microcystis a. PCC 7941 201
Microcystis a. PCC 9443 201
Microcystis a. PCC 9701 201
Microcystis a. PCC 9717 201
Microcystis a. PCC 9806 201
Microcystis a. PCC 9807 201
Microcystis sp. T1-4 201
Moorea p. 3L 201
Nodularia s. CCY9414 199
Nostoc a. 0708 199
Nostoc c. 199
Nostoc p. PCC 73102 200
Nostoc sp. PCC 7120 199
Nostoc sp. PCC 9709 200
Oscillatoria sp. PCC 6506 200
Raphidiopsis b. D9 202
Synechococcus e. PCC 6301 200
Synechococcus sp. PCC 7335 204
Synechococcus sp. BL107 185
Synechococcus sp. CB0101 196
Synechococcus sp. CB0205 196
Synechococcus sp. CC9311 196
Synechococcus sp. CC9605 196
Synechococcus sp. CC9902 196
Synechococcus sp. JA-2-3B 221
Synechococcus sp. JA-3-3Ab 217
Synechococcus sp. PCC 7002 197
Synechococcus sp. RCC307 200
Synechococcus sp. RS9916 196
Synechococcus sp. RS9917 203
Synechococcus sp. WH 5701 196
Synechococcus sp. WH 7803 196
Synechococcus sp. WH 7805 196
Synechococcus sp. WH 8016 185
Synechococcus sp. WH 8102 196
Synechococcus sp. WH 8109 185
Synechocystis sp. PCC 6803 201
Thermosynechococcus e. BP-1 201
Trichodesmium e. IMS101 200
consensus
logo

		β12 · α9 · β13 · β14 · α10 (positions 260, 270, 280, 290, 300)
Synechococcus e. PCC 7942	255	TQRSSNVRVSSGVVRLDEMCGGGFFKDSIILATGATGTGKTLLVSRFVENACANK
Acaryochloris m. MBIC11017	256	TQRSSNARVSSGVQTLDEMCGGGFFKDSIILVTGATGTGKTLLVSKFLEDACKNG
Acaryochloris sp. CCMEE 5410	256	TQRSSNARVSSGVQTLDEMCGGGFFKDSIILVTGATGTGKTLLVSKFLENACSNG
Anabaena v. ATCC 29413	254	TQRSSNVRVSSGVKTLDGMCGGGFFKDSIILATGATGTGKTLLVSKFLQNGCVNN
Arthrospira m. CS-328	255	TQRSSNVRVSSGNKTLDEMCGGGFFKDSIILATGATGTGKTLLVSIFVNNACQSN
Arthrospira m. FACHB-438	255	TQRSSNIRVSSGDKILDEMCGGGFFKDSIILATGATGTGKTLLVSIFVNNACQSN
Arthrospira p. str. Paraca	255	TQRSSNIRVSSGDKTLDEMCGGGFFKDSIILATGATGTGKTLLVSIFVNNACQSN
Crocosphaera w. WH 0003	256	TQRSSNARSSSGVKTLDQMCGGGFFKDSIILATGATGTGKTLLVSKFLEEGCRQG
Crocosphaera w. WH 8501	256	TQRSSNARSSSGVKTLDQMCGGGFFKDSIILATGATGTGKTLLVSKFLEEGCRQG
Cyanobium sp. PCC 7001	251	TQRSSNIRISSGVPKLDEMCGGGFFKDSIILATGATGTGKTLLVSKFVEDACRSK
Cyanothece sp. ATCC 51142	256	TQRSSNARISSGVETLDEMCGGGFFKDSIILATGATGTGKTLLVSKFLEEGCRQG
Cyanothece sp. CCY0110	232	TQRSSNARVSSGVATLDEMCGGGFFKDSIILATGATGTGKTLLVSKFLEEGCRQG
Cyanothece sp. PCC 7424	256	TQRSSNVRISSGVKTLDEMCGGGFFKDSIILATGATGTGKTLLVSKFLEEGCRRG
Cyanothece sp. PCC 7425	265	TQRSSNVRVSSGVKTLDEMCGGGFFKDSIILVTGATGTGKTLLVSKFLENACLNG
Cyanothece sp. PCC 7822	256	TQRSSNVRISSGVKTLDEMCGGGFFKDSIILATGATGTGKTLLVSKFLEEGCRRG
Cyanothece sp. PCC 8801	256	TQRSSNARISSGVKTLDEMCGGGFFKDSIILATGATGTGKTLLVSKFLEEGCRQG
Cyanothece sp. PCC 8802	256	TQRSSNARISSGVKTLDEMCGGGFFKDSIILATGATGTGKTLLVSKFLEEGCRQG
Cylindrospermopsis r. CS-505	257	TQRSSNVRVSSGVNTLDEMCGGGFFKDSIILATGATGTGKTLLVSKFIQDGCVSG
Fischerella sp. JSC-11	255	TQRSSNVRVSSGVKTLDDMCGGGFFKDSIILATGATGTGKTLLVSKFIQNGCQNG
Leptolyngbya b. IAM M-101	255	TQRSSNVRVSSGVNTLDEMCGGGFFKDSIILATGATGTGKTLLVSKFLQEACQRG
Lyngbya sp. PCC 8106	256	TQRSSNVRVSSGNETLDQMCGGGFFKDSIILATGATGTGKTLLVSVFINNACKNG
Microcoleus c. PCC 7420	256	TQRSSNVRVSSGIKTLDEMCGGGFFKDSIILATGATGTGKTLLVSKFIHDACLNE
Microcoleus v. FGP-2	255	TQKSSNVRVSSGDKTLDEMCGGGFFKDSIILATGATGTGKTLLVSKFLQNACMNG
Microcystis a. NIES-843	256	TQRSSNVRISSGVKTLDDMCGGGFFKDSIILATGATGTGKTLLVSKFLEEGCRRG
Microcystis a. PCC 7806	256	TQRSSNVRISSGVKTLDDMCGGGFFKDSIILATGATGTGKTLLVSKFLEEGCRRG
Microcystis a. PCC 7820	256	TQRSSNVRISSGVKTLDDMCGGGFFKDSIILATGATGTGKTLLVSKFLEEGCRRG
Microcystis a. PCC 7941	256	TQRSSNVRISSGVKTLDDMCGGGFFKDSIILATGATGTGKTLLVSKFLEEGCRRG
Microcystis a. PCC 9443	256	TQRSSNVRISSGVKTLDDMCGGGFFKDSIILATGATGTGKTLLVSKFLEEGCRRG
Microcystis a. PCC 9701	256	TQRSSNVRISSGVKTLDDMCGGGFFKDSIILATGATGTGKTLLVSKFLEEGCRRG
Microcystis a. PCC 9717	256	TQRSSNVRISSGVKTLDDMCGGGFFKDSIILATGATGTGKTLLVSKFLEEGCRRG
Microcystis a. PCC 9806	256	TQRSSNVRISSGVKTLDDMCGGGFFKDSIILATGATGTGKTLLVSKFLEEGCRRG
Microcystis a. PCC 9807	256	TQRSSNVRISSGVKTLDDMCGGGFFKDSIILATGATGTGKTLLVSKFLEEGCRRG
Microcystis sp. T1-4	256	TQRSSNVRISSGVKTLDDMCGGGFFKDSIILATGATGTGKTLLVSKFLEEGCRRG
Moorea p. 3L	256	TQRSSMARVSSGINTLDVMCGGGFFKDSIILATGATGTGKTLLVSKFIQNACIQG
Nodularia s. CCY9414	254	TQRSSNVRVSSGVKTLDEMCGGGFFKDSIILATGATGTGKTLLVSKFLQDGCRNG
Nostoc a. 0708	254	TQRSSNVRVSSGVKTLDEMCGGGFFKDSIILATGATGTGKTLLVSKFIQDGCVSG
Nostoc c.	254	TQRSSNVRVSSGVKTLDEMCGGGFFKDSIILATGATGTGKTLLVSKFLQNGCVNG
Nostoc p. PCC 73102	255	TQRSSNIRVSSGVKILDEMCGGGFFKDSIILATGATGTGKTLLVSKFIQDGCLNG
Nostoc sp. PCC 7120	254	TQRSSNVRVSSGVKTLDGMCGGGFFKDSIILATGATGTGKTLLVSKFLQNGCVNN
Nostoc sp. PCC 9709	255	TQRSSNVRVSSGVKTLDEMCGGGFFKDSIILATGATGTGKTLLVSKFIQDGCLNG
Oscillatoria sp. PCC 6506	255	TQRSSNVRVSSGDKTLDEMCGGGFFKDSIILATGATGTGKTLLVSKFLQNACANG
Raphidiopsis b. D9	257	TQRSSNVRVSSGVNTLDEMCGGGFFKDSIILATGATGTGKTLLVSKFIQDGCVSG
Synechococcus e. PCC 6301	255	TQRSSNVRVSSGVVRLDEMCGGGFFKDSIILATGATGTGKTLLVSRFVENACANK
Synechococcus sp. PCC 7335	259	TQRSSNVRVSSGVKTLDEMCGGGFFRDSIILATGATGTGKTLLVSNFLANACENG
Synechococcus sp. BL107	240	TQRSSNVRVSSGVPRLDEMCGGGFFKDSIILATGATGTGKTMLVSKFIEDACRNK
Synechococcus sp. CB0101	251	TQRSSNVRVSSGVPRLDEMCGGGFFKDSIILATGATGTGKTLLVSKFVENACANK
Synechococcus sp. CB0205	251	TQRSSNVRVSSGVPRLDEMCGGGFFKDSIILATGATGTGKTLLVSKFVENACANK
Synechococcus sp. CC9311	251	TQRSSNVRVSSGVPRLDEMCGGGYFKDSIILATGATGTGKTLLISKFIEDACNNK
Synechococcus sp. CC9605	251	TQRSSNVRVSSGVPRLDDMCGGGFFKDSIILATGATGTGKTMLVSKFIEDACRNK
Synechococcus sp. CC9902	251	TQRSSNVRVSSGVPRLDEMCGGGFFKDSIILATGATGTGKTMLVSKFIEDACRNK
Synechococcus sp. JA-2-3B	276	TQRSSNVRVSSGIEKLDEMCGGGFFKDSIILATGATGTGKTSLVSKFLERGCLDG
Synechococcus sp. JA-3-3Ab	272	TQRSSNVRVSSGIEKLDEMCGGGFFKDSIILATGATGTGKTSLVSKFLERGCLNG
Synechococcus sp. PCC 7002	252	TQRSSNARISSGVETLDKMCGGGFFKDSIILATGATGTGKTLLVSKFLEEGCRRG
Synechococcus sp. RCC307	255	TQSSSNVRLSSGVPRLDEMCGGGFFKDSIILATGATGTGKTLLVSKFLEDACRNK
Synechococcus sp. RS9916	251	TQRSSNVRVSSGVQRLDDMCGGGFFKDSIILATGATGTGKTLLVSKFIEDACRNK
Synechococcus sp. RS9917	258	TQRSSNVRLSSGVPRLDEMCGGGFFKDSIILATGATGTGKTLLVSKFIEDACRNK
Synechococcus sp. WH 5701	251	TQRSSNVRISSGVLRLDEMCGGGFFKDSIILATGATGTGKTLLVSKFVEDAYNNK
Synechococcus sp. WH 7803	251	TQRSSNVRVSSGVPRLDEMCGGGFFKDSIILATGATGTGKTLLVSKFIEDACRNE
Synechococcus sp. WH 7805	251	TQRSSNVRVSSGVPRLDEMCGGGFFKDSIILATGATGTGKTLLVSKFIEDACRNK
Synechococcus sp. WH 8016	240	TQRSSNVRVSSGVPRLDEMCGGGYFKDSIILATGATGTGKTLLVSKFIEDACSNK
Synechococcus sp. WH 8102	251	TQRSSNVRVSSGVPRLDEMCGGGFFKDSIILATGATGTGKTLLVSKFIEDACRNK
Synechococcus sp. WH 8109	240	TQRSSNVRVSSGVPRLDDMCGGGFFKDSIILATGATGTGKTMLVSKFLEDACRNK
Synechocystis sp. PCC 6803	256	TQRSSNARISSGVQTLDEMCGGGFFKDSIILATGATGTGKTLLVSKFLQEGCRQR
Thermosynechococcus e. BP-1	255	TQRSSNVRVSSGVKTLDEMCGGGFFKDSIILATGATGTGKTLLVSKFLETGCQQG
Trichodesmium e. IMS101	255	TQRSSNVRVSSGDEILDKMCGGGFFKDSIILATGATGTGKTLLVSKFIEDGCMNN
consensus		!!*!!**!*!!!* !!*!!!!!*!*!!!!!*!!!!!!!!!*!*!*!* **

logo

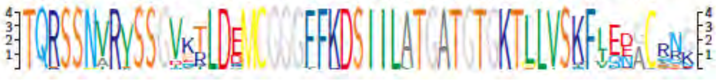

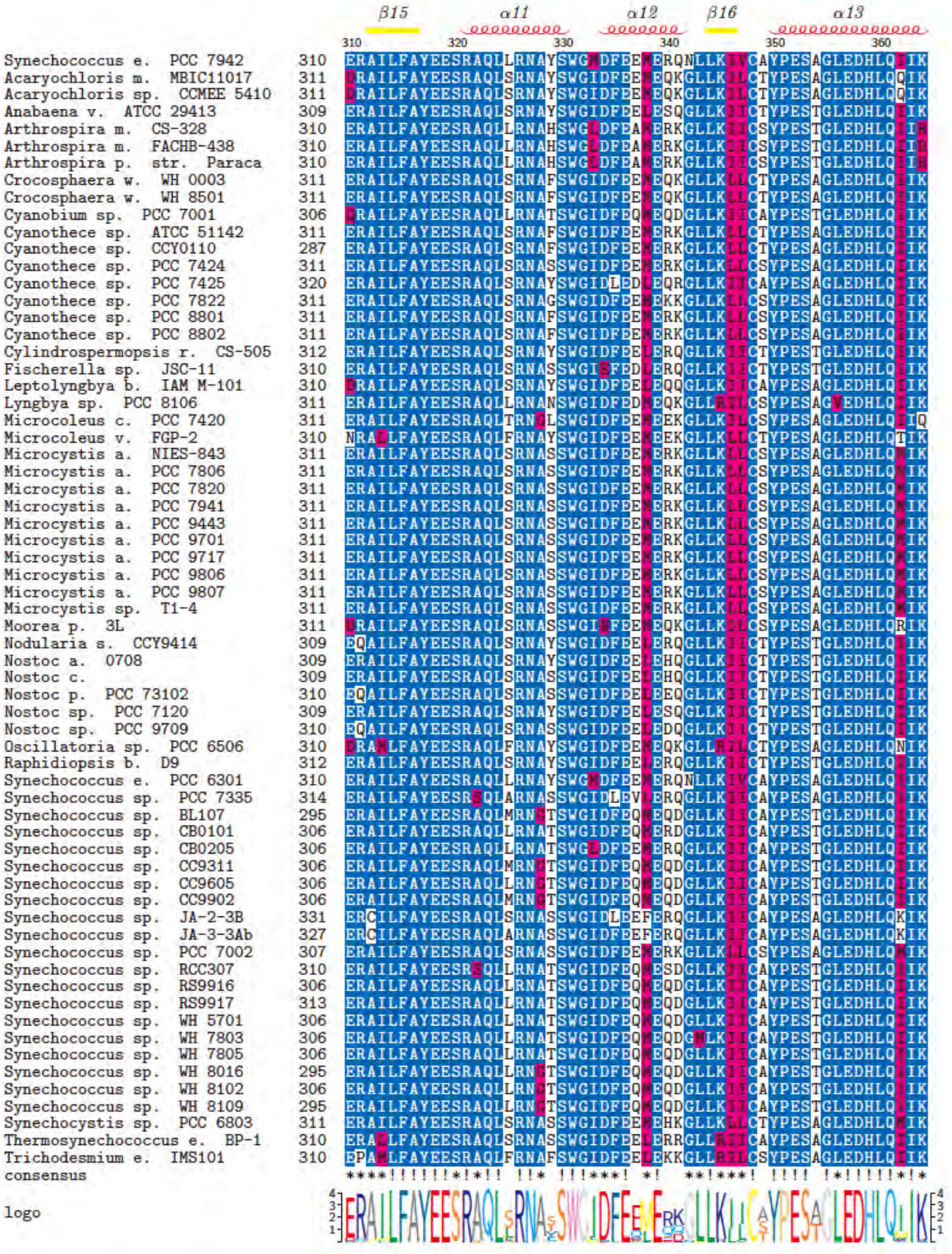

β15
α11
α12
β16
α13
310
320
330
340
350
360
Synechococcus e. PCC 7942 310
Acaryochloris m. MBIC11017 311
Acaryochloris sp. CCMEE 5410 311
Anabaena v. ATCC 29413 309
Arthrospira m. CS-328 310
Arthrospira m. FACHB-438 310
Arthrospira p. str. Paraca 310
Crocosphaera w. WH 0003 311
Crocosphaera w. WH 8501 311
Cyanobium sp. PCC 7001 306
Cyanothece sp. ATCC 51142 311
Cyanothece sp. CCY0110 287
Cyanothece sp. PCC 7424 311
Cyanothece sp. PCC 7425 320
Cyanothece sp. PCC 7822 311
Cyanothece sp. PCC 8801 311
Cyanothece sp. PCC 8802 311
Cylindrospermopsis r. CS-505 312
Fischerella sp. JSC-11 310
Leptolyngbya b. IAM M-101 310
Lyngbya sp. PCC 8106 311
Microcoleus c. PCC 7420 311
Microcoleus v. FGP-2 310
Microcystis a. NIES-843 311
Microcystis a. PCC 7806 311
Microcystis a. PCC 7820 311
Microcystis a. PCC 7941 311
Microcystis a. PCC 9443 311
Microcystis a. PCC 9701 311
Microcystis a. PCC 9717 311
Microcystis a. PCC 9806 311
Microcystis a. PCC 9807 311
Microcystis sp. T1-4 311
Moorea p. 3L 311
Nodularia s. CCY9414 309
Nostoc a. 0708 309
Nostoc c. 309
Nostoc p. PCC 73102 310
Nostoc sp. PCC 7120 309
Nostoc sp. PCC 9709 310
Oscillatoria sp. PCC 6506 310
Raphidiopsis b. D9 312
Synechococcus e. PCC 6301 310
Synechococcus sp. PCC 7335 314
Synechococcus sp. BL107 295
Synechococcus sp. CB0101 306
Synechococcus sp. CB0205 306
Synechococcus sp. CC9311 306
Synechococcus sp. CC9605 306
Synechococcus sp. CC9902 306
Synechococcus sp. JA-2-3B 331
Synechococcus sp. JA-3-3Ab 327
Synechococcus sp. PCC 7002 307
Synechococcus sp. RCC307 310
Synechococcus sp. RS9916 306
Synechococcus sp. RS9917 313
Synechococcus sp. WH 5701 306
Synechococcus sp. WH 7803 306
Synechococcus sp. WH 7805 306
Synechococcus sp. WH 8016 295
Synechococcus sp. WH 8102 306
Synechococcus sp. WH 8109 295
Synechocystis sp. PCC 6803 311
Thermosynechococcus e. BP-1 310
Trichodesmium e. IMS101 310
consensus
logo

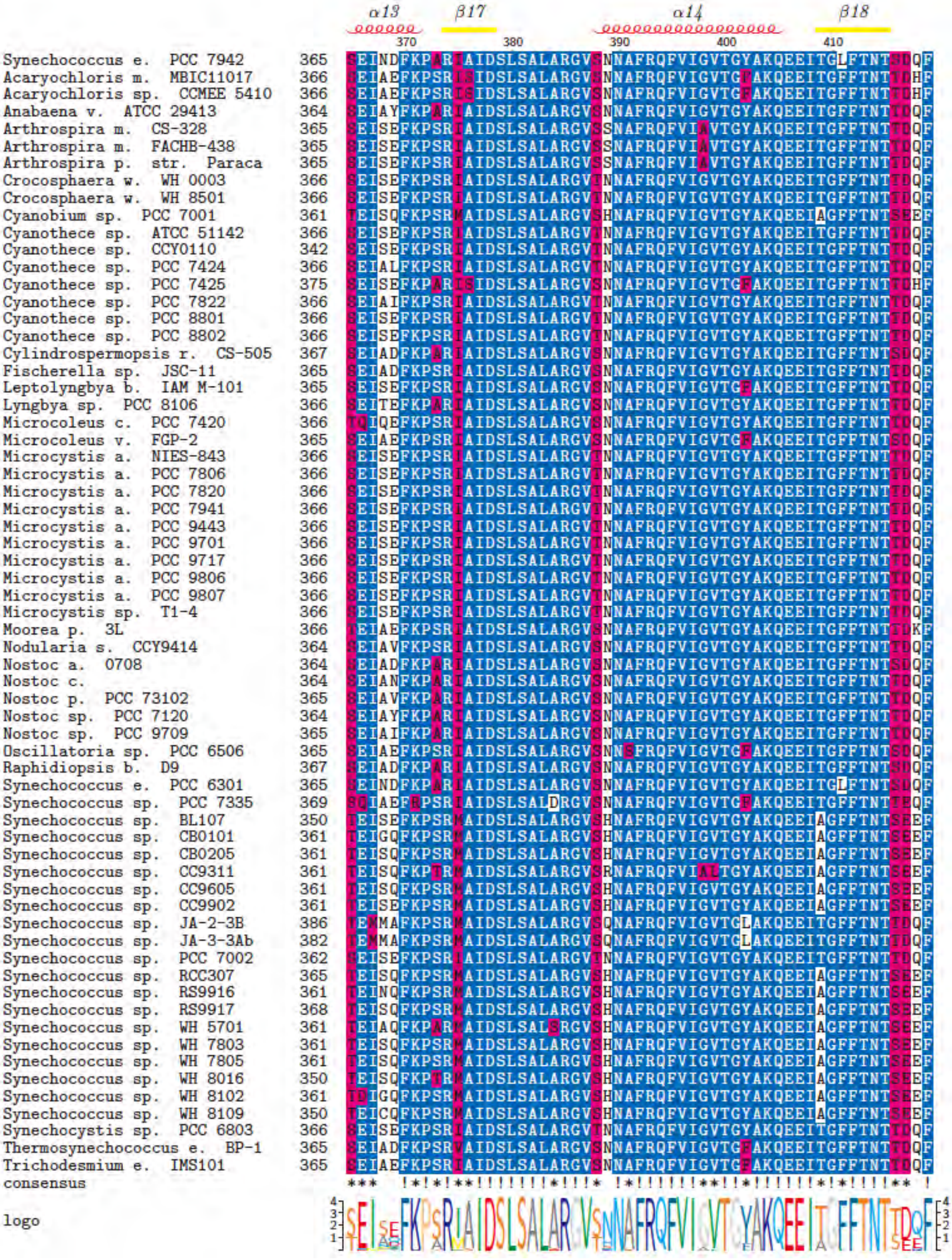
α13
β17
α14
β18
370
380
390
400
410
Synechococcus e. PCC 7942 365 SEINDFKPARIAIDSLSALARGVSNNAFRQFVIGVTGYAKQEEITGLFTNTSDQF
Acaryochloris m. MBIC11017 366 SEIAEFKPSRISIDSLSALARGVSNNAFRQFVIGVTGFAKQEEITGFFTNTTDHF
Acaryochloris sp. CCMEE 5410 366 SEIAEFKPSRISIDSLSALARGVSNNAFRQFVIGVTGFAKQEEITGFFTNTTDHF
Anabaena v. ATCC 29413 364 SEIAYFKPARIAIDSLSALARGVSNNAFRQFVIGVTGYAKQEEITGFFTNTTDQF
Arthrospira m. CS-328 365 SEISEFKPSRIAIDSLSALARGVSSNAFRQFVIAVTGYAKQEEITGFFTNTTDQF
Arthrospira m. FACHB-438 365 SEISEFKPSRIAIDSLSALARGVSSNAFRQFVIAVTGYAKQEEITGFFTNTTDQF
Arthrospira p. str. Paraca 365 SEISEFKPSRIAIDSLSALARGVSSNAFRQFVIAVTGYAKQEEITGFFTNTTDQF
Crocosphaera w. WH 0003 366 SEISEFKPSRIAIDSLSALARGVTNNAFRQFVIGVTGYAKQEEITGFFTNTTDQF
Crocosphaera w. WH 8501 366 SEISEFKPSRIAIDSLSALARGVTNNAFRQFVIGVTGYAKQEEITGFFTNTTDQF
Cyanobium sp. PCC 7001 361 TEISQFKPSRMAIDSLSALARGVSHNAFRQFVIGVTGYAKQEEIAGFFTNTSEEF
Cyanothece sp. ATCC 51142 366 SEISEFKPSRIAIDSLSALARGVTNNAFRQFVIGVTGYAKQEEITGFFTNTTDQF
Cyanothece sp. CCY0110 342 SEISEFKPSRIAIDSLSALARGVTNNAFRQFVIGVTGYAKQEEITGFFTNTTDQF
Cyanothece sp. PCC 7424 366 SEIALFKPSRIAIDSLSALARGVTNNAFRQFVIGVTGYAKQEEITGFFTNTTDQF
Cyanothece sp. PCC 7425 375 SEISEFKPARISIDSLSALARGVSNNAFRQFVIGVTGFAKQEEITGFFTNTTDHF
Cyanothece sp. PCC 7822 366 SEIAIFKPSRIAIDSLSALARGVTNNAFRQFVIGVTGYAKQEEITGFFTNTTDQF
Cyanothece sp. PCC 8801 366 SEISEFKPSRIAIDSLSALARGVTNNAFRQFVIGVTGYAKQEEITGFFTNTTDQF
Cyanothece sp. PCC 8802 366 SEISEFKPSRIAIDSLSALARGVTNNAFRQFVIGVTGYAKQEEITGFFTNTTDQF
Cylindrospermopsis r. CS-505 367 SEIADFKPARIAIDSLSALARGVSNNAFRQFVIGVTGYAKQEEITGFFTNTSDQF
Fischerella sp. JSC-11 365 SEIADFKPSRIAIDSLSALARGVSNNAFRQFVIGVTGYAKQEEITGFFTNTTDQF
Leptolyngbya b. IAM M-101 365 SEISEFKPSRIAIDSLSALARGVSNNAFRQFVIGVTGFAKQEEITGFFTNTTDQF
Lyngbya sp. PCC 8106 366 SEITEFKPARIAIDSLSALARGVSNNAFRQFVIGVTGYAKQEEITGFFTNTTDQF
Microcoleus c. PCC 7420 366 TQIQEFKPSRIAIDSLSALARGVSNNAFRQFVIGVTGYAKQEEITGFFTNTTDQF
Microcoleus v. FGP-2 365 SEIAEFKPSRIAIDSLSALARGVSNNAFRQFVIGVTGFAKQEEITGFFTNTSDQF
Microcystis a. NIES-843 366 SEISEFKPSRIAIDSLSALARGVTNNAFRQFVIGVTGYAKQEEITGFFTNTTDQF
Microcystis a. PCC 7806 366 SEISEFKPSRIAIDSLSALARGVTNNAFRQFVIGVTGYAKQEEITGFFTNTTDQF
Microcystis a. PCC 7820 366 SEISEFKPSRIAIDSLSALARGVTNNAFRQFVIGVTGYAKQEEITGFFTNTTDQF
Microcystis a. PCC 7941 366 SEISEFKPSRIAIDSLSALARGVTNNAFRQFVIGVTGYAKQEEITGFFTNTTDQF
Microcystis a. PCC 9443 366 SEISEFKPSRIAIDSLSALARGVTNNAFRQFVIGVTGYAKQEEITGFFTNTTDQF
Microcystis a. PCC 9701 366 SEISEFKPSRIAIDSLSALARGVTNNAFRQFVIGVTGYAKQEEITGFFTNTTDQF
Microcystis a. PCC 9717 366 SEISEFKPSRIAIDSLSALARGVTNNAFRQFVIGVTGYAKQEEITGFFTNTTDQF
Microcystis a. PCC 9806 366 SEISEFKPSRIAIDSLSALARGVTNNAFRQFVIGVTGYAKQEEITGFFTNTTDQF
Microcystis a. PCC 9807 366 SEISEFKPSRIAIDSLSALARGVTNNAFRQFVIGVTGYAKQEEITGFFTNTTDQF
Microcystis sp. T1-4 366 SEISEFKPSRIAIDSLSALARGVTNNAFRQFVIGVTGYAKQEEITGFFTNTTDQF
Moorea p. 3L 366 TEIAEFKPSRIAIDSLSALARGVSNNAFRQFVIGVTGYAKQEEITGFFTNTTDKF
Nodularia s. CCY9414 364 SEIAVFKPSRIAIDSLSALARGVSNNAFRQFVIGVTGYAKQEEITGFFTNTTDQF
Nostoc a. 0708 364 SEIADFKPARIAIDSLSALARGVSNNAFRQFVIGVTGYAKQEEITGFFTNTSDQF
Nostoc c. 364 SEIANFKPARIAIDSLSALARGVSNNAFRQFVIGVTGYAKQEEITGFFTNTTDQF
Nostoc p. PCC 73102 365 SEIAVFKPARIAIDSLSALARGVSNNAFRQFVIGVTGYAKQEEITGFFTNTTDQF
Nostoc sp. PCC 7120 364 SEIAYFKPARIAIDSLSALARGVSNNAFRQFVIGVTGYAKQEEITGFFTNTTDQF
Nostoc sp. PCC 9709 365 SEIAIFKPARIAIDSLSALARGVSNNAFRQFVIGVTGYAKQEEITGFFTNTTDQF
Oscillatoria sp. PCC 6506 365 SEIAEFKPSRIAIDSLSALARGVSNNSFRQFVIGVTGFAKQEEITGFFTNTSDQF
Raphidiopsis b. D9 367 SEIADFKPARIAIDSLSALARGVSNNAFRQFVIGVTGYAKQEEITGFFTNTSDQF
Synechococcus e. PCC 6301 365 SEINDFKPARIAIDSLSALARGVSNNAFRQFVIGVTGYAKQEEITGLFTNTSDQF
Synechococcus sp. PCC 7335 369 SQIAEFRPSRIAIDSLSALDRGVSNNAFRQFVIGVTGFAKQEEITGFFTNTTEQF
Synechococcus sp. BL107 350 TEISEFKPSRMAIDSLSALARGVSHNAFRQFVIGVTGYAKQEEIAGFFTNTSEEF
Synechococcus sp. CB0101 361 TEIGQFKPSRMAIDSLSALARGVSHNAFRQFVIGVTGYAKQEEIAGFFTNTSEEF
Synechococcus sp. CB0205 361 TEISQFKPSRMAIDSLSALARGVSHNAFRQFVIGVTGYAKQEEIAGFFTNTSEEF
Synechococcus sp. CC9311 361 TEISQFKPTRMAIDSLSALARGVSRNAFRQFVIALTGYAKQEEIAGFFTNTSEEF
Synechococcus sp. CC9605 361 TEISQFKPSRMAIDSLSALARGVSHNAFRQFVIGVTGYAKQEEIAGFFTNTSEEF
Synechococcus sp. CC9902 361 TEISEFKPSRMAIDSLSALARGVSHNAFRQFVIGVTGYAKQEEIAGFFTNTSEEF
Synechococcus sp. JA-2-3B 386 TEMMAFKPSRMAIDSLSALARGVSQNAFRQFVIGVTGLAKQEEITGFFTNTTDQF
Synechococcus sp. JA-3-3Ab 382 TEMMAFKPSRMAIDSLSALARGVSQNAFRQFVIGVTGLAKQEEITGFFTNTTDQF
Synechococcus sp. PCC 7002 362 SEISEFKPSRIAIDSLSALARGVTNNAFRQFVIGVTGYAKQEEITGFFTNTTDQF
Synechococcus sp. RCC307 365 TEISQFKPSRMAIDSLSALARGVSHNAFRQFVIGVTGYAKQEEIAGFFTNTSEEF
Synechococcus sp. RS9916 361 TEINQFKPSRMAIDSLSALARGVSHNAFRQFVIGVTGYAKQEEIAGFFTNTSEEF
Synechococcus sp. RS9917 368 TEISQFKPSRMAIDSLSALARGVSHNAFRQFVIGVTGYAKQEEIAGFFTNTSEEF
Synechococcus sp. WH 5701 361 TEIAQFKPARMAIDSLSALSRGVSHNAFRQFVIGVTGYAKQEEIAGFFTNTSEEF
Synechococcus sp. WH 7803 361 TEISQFKPSRMAIDSLSALARGVSHNAFRQFVIGVTGYAKQEEIAGFFTNTSEEF
Synechococcus sp. WH 7805 361 TEISQFKPSRMAIDSLSALARGVSHNAFRQFVIGVTGYAKQEEIAGFFTNTSEEF
Synechococcus sp. WH 8016 350 TEISQFKPTRMAIDSLSALARGVSHNAFRQFVIGVTGYAKQEEIAGFFTNTSEEF
Synechococcus sp. WH 8102 361 TDIGQFKPSRMAIDSLSALARGVSHNAFRQFVIGVTGYAKQEEIAGFFTNTSEEF
Synechococcus sp. WH 8109 350 TEICQFKPSRMAIDSLSALARGVSHNAFRQFVIGVTGYAKQEEIAGFFTNTSEEF
Synechocystis sp. PCC 6803 366 SEISEFKPSRIAIDSLSALARGVTNNAFRQFVIGVTGYAKQEEITGFFTNTTDQF
Thermosynechococcus e. BP-1 365 SEIADFKPSRVAIDSLSALARGVSNNAFRQFVIGVTGFAKQEEITGFFTNTTDQF
Trichodesmium e. IMS101 365 SEIAEFKPSRIAIDSLSALARGVSNNAFRQFVIGVTGFAKQEEITGFFTNTTDQF
consensus *** !*!*!**!!!!!!!*!!!* !*!!!!!!**!!*!!!!!!*!*!!!!** !
logo

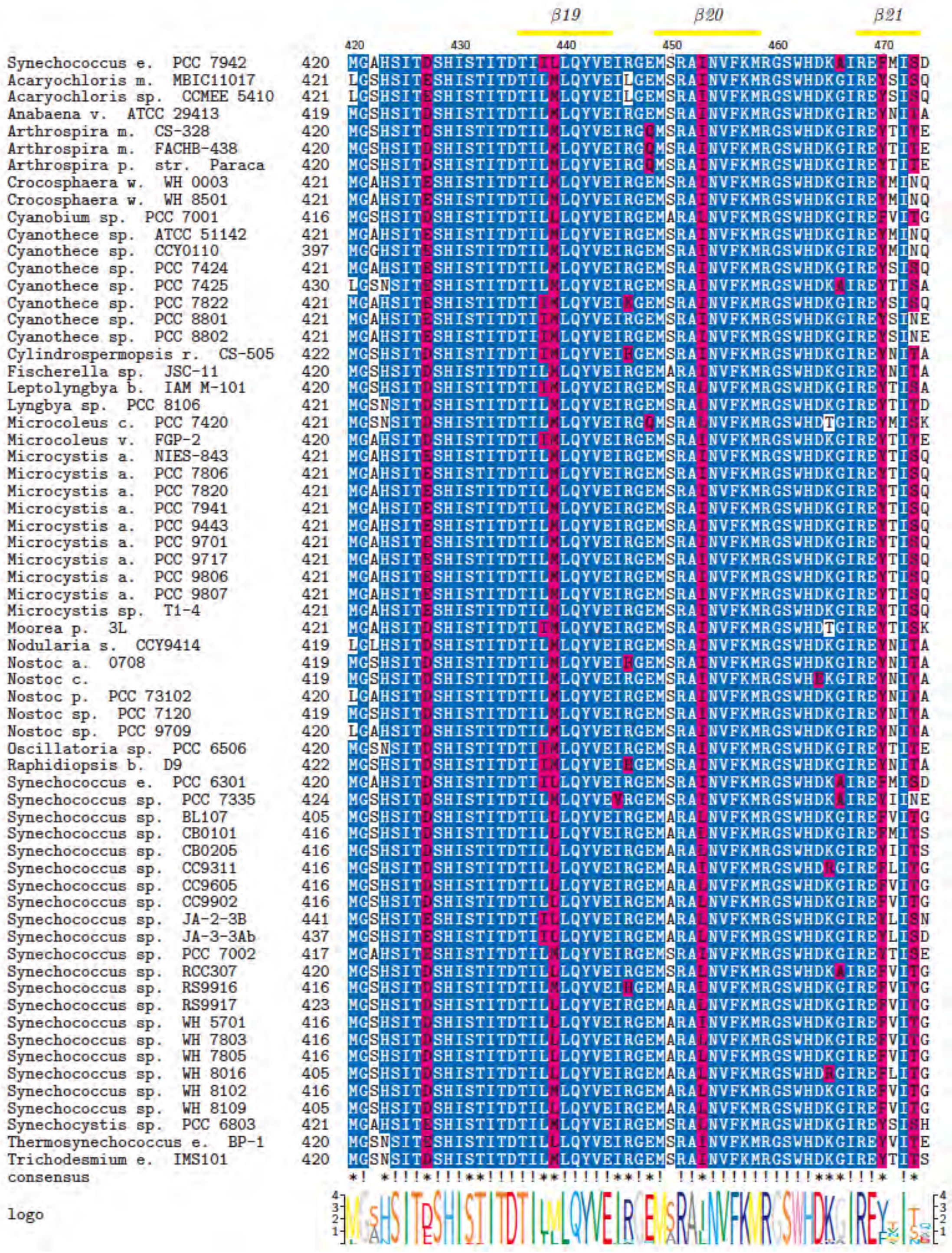
β19
β20
β21
420
430
440
450
460
470
Synechococcus e. PCC 7942 420
Acaryochloris m. MBIC11017 421
Acaryochloris sp. CCMEE 5410 421
Anabaena v. ATCC 29413 419
Arthrospira m. CS-328 420
Arthrospira m. FACHB-438 420
Arthrospira p. str. Paraca 420
Crocosphaera w. WH 0003 421
Crocosphaera w. WH 8501 421
Cyanobium sp. PCC 7001 416
Cyanothece sp. ATCC 51142 421
Cyanothece sp. CCY0110 397
Cyanothece sp. PCC 7424 421
Cyanothece sp. PCC 7425 430
Cyanothece sp. PCC 7822 421
Cyanothece sp. PCC 8801 421
Cyanothece sp. PCC 8802 421
Cylindrospermopsis r. CS-505 422
Fischerella sp. JSC-11 420
Leptolyngbya b. IAM M-101 420
Lyngbya sp. PCC 8106 421
Microcoleus c. PCC 7420 421
Microcoleus v. FGP-2 420
Microcystis a. NIES-843 421
Microcystis a. PCC 7806 421
Microcystis a. PCC 7820 421
Microcystis a. PCC 7941 421
Microcystis a. PCC 9443 421
Microcystis a. PCC 9701 421
Microcystis a. PCC 9717 421
Microcystis a. PCC 9806 421
Microcystis a. PCC 9807 421
Microcystis sp. T1-4 421
Moorea p. 3L 421
Nodularia s. CCY9414 419
Nostoc a. 0708 419
Nostoc c. 419
Nostoc p. PCC 73102 420
Nostoc sp. PCC 7120 419
Nostoc sp. PCC 9709 420
Oscillatoria sp. PCC 6506 420
Raphidiopsis b. D9 422
Synechococcus e. PCC 6301 420
Synechococcus sp. PCC 7335 424
Synechococcus sp. BL107 405
Synechococcus sp. CB0101 416
Synechococcus sp. CB0205 416
Synechococcus sp. CC9311 416
Synechococcus sp. CC9605 416
Synechococcus sp. CC9902 416
Synechococcus sp. JA-2-3B 441
Synechococcus sp. JA-3-3Ab 437
Synechococcus sp. PCC 7002 417
Synechococcus sp. RCC307 420
Synechococcus sp. RS9916 416
Synechococcus sp. RS9917 423
Synechococcus sp. WH 5701 416
Synechococcus sp. WH 7803 416
Synechococcus sp. WH 7805 416
Synechococcus sp. WH 8016 405
Synechococcus sp. WH 8102 416
Synechococcus sp. WH 8109 405
Synechocystis sp. PCC 6803 421
Thermosynechococcus e. BP-1 420
Trichodesmium e. IMS101 420
consensus
logo

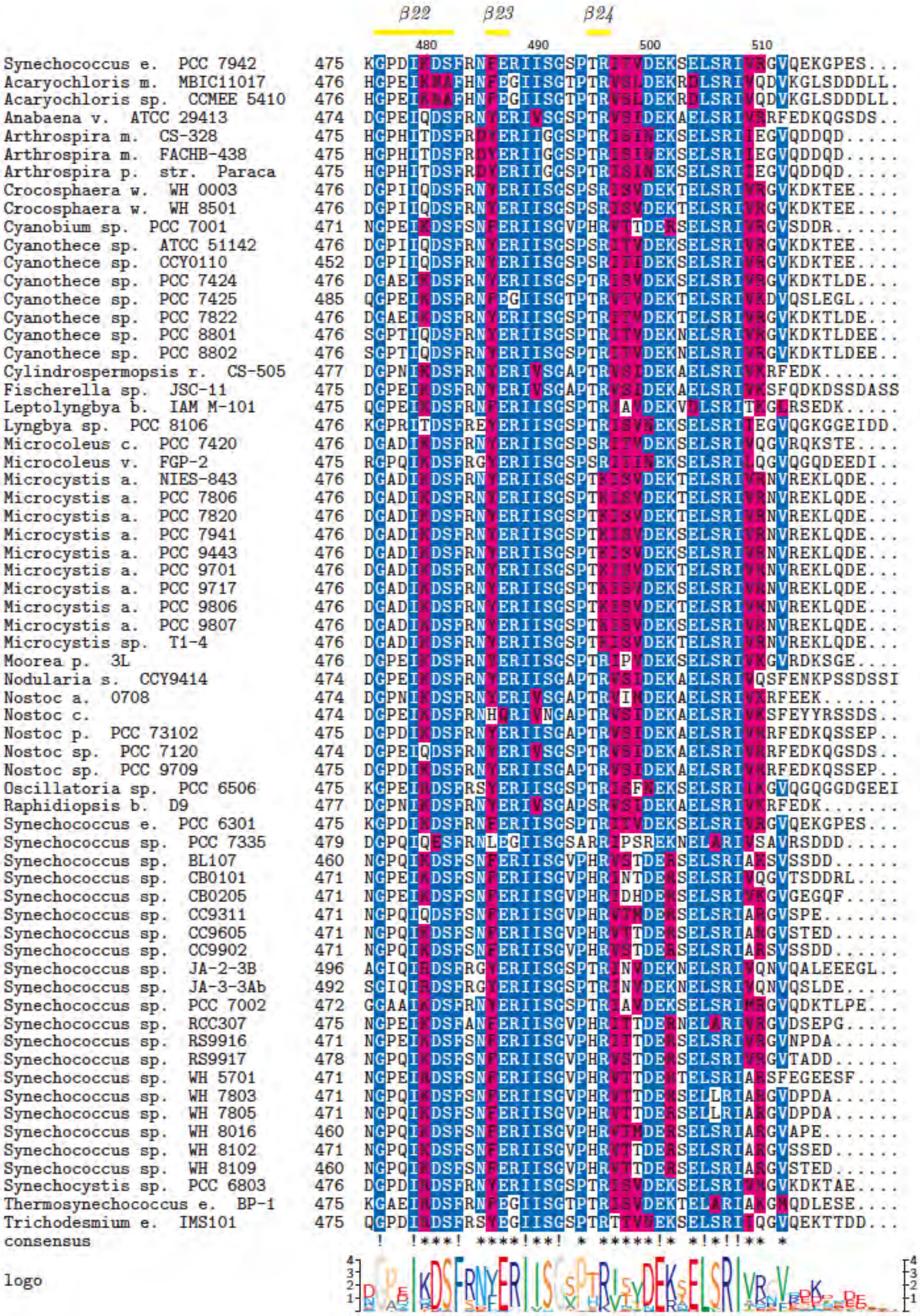
β22 β23 β24
480 490 500 510
Synechococcus e. PCC 7942 475 KGPDIKDSFRNFERIISGSPTRITVDEKSELSRIVRGVQEKGPES...
Acaryochloris m. MBIC11017 476 HGPEIKNAFHNFEGIISGTPTRVSLDEKRDLSRIVQDVKGLSDDDLL.
Acaryochloris sp. CCMEE 5410 476 HGPEIKNAFHNFEGIISGTPTRVSLDEKRDLSRIVQDVKGLSDDDLL.
Anabaena v. ATCC 29413 474 DGPEIQDSFRNYERIVSGSPTRVSIDEKAELSRIVRRFEDKQGSDS..
Arthrospira m. CS-328 475 HGPHITDSFRDYERIIGGSPTRISINEKSELSRIIEGVQDDQD.....
Arthrospira m. FACHB-438 475 HGPHITDSFRDYERIIGGSPTRISINEKSELSRIIEGVQDDQD.....
Arthrospira p. str. Paraca 475 HGPHITDSFRDYERIIGGSPTRISINEKSELSRIIEGVQDDQD.....
Crocosphaera w. WH 0003 476 DGPIIQDSFRNYERIISGSPSRISVDEKTELSRIVRGVKDKTEE....
Crocosphaera w. WH 8501 476 DGPIIQDSFRNYERIISGSPSRISVDEKTELSRIVRGVKDKTEE....
Cyanobium sp. PCC 7001 471 NGPEIKDSFSNFERIISGVPHRVTTDERSELSRIVRGVSDDR......
Cyanothece sp. ATCC 51142 476 DGPIIQDSFRNYERIISGSPSRITVDEKSELSRIVRGVKDKTEE....
Cyanothece sp. CCY0110 452 DGPIIQDSFRNYERIISGSPSRITIDEKSELSRIVRGVKDKTEE....
Cyanothece sp. PCC 7424 476 DGAEIKDSFRNYERIISGSPTRISVDEKSELSRIVRGVKDKTLDE...
Cyanothece sp. PCC 7425 485 QGPEIKDSFRNFEGIISGTPTRVTVDEKTELSRIVKDVQSLEGL....
Cyanothece sp. PCC 7822 476 DGAEIKDSFRNYERIISGSPTRITVDEKTELSRIVRGVKDKTLDE...
Cyanothece sp. PCC 8801 476 SGPTIQDSFRNYERIISGSPTRITVDEKNELSRIVRGVKDKTLDEE..
Cyanothece sp. PCC 8802 476 SGPTIQDSFRNYERIISGSPTRITVDEKNELSRIVRGVKDKTLDEE..
Cylindrospermopsis r. CS-505 477 DGPNIKDSFRNYERIVSGAPTRVSIDEKAELSRIVKRFEDK.......
Fischerella sp. JSC-11 475 DGPEIKDSFRNYERIVSGAPTRVSIDEKAELSRIVKSFQDKDSSDASS
Leptolyngbya b. IAM M-101 475 QGPEIKDSFRNFERIISGSPTRIAVDEKVDLSRITKGLRSEDK.....
Lyngbya sp. PCC 8106 476 KGPRITDSFREYERIISGSPTRISVNEKSELSRIIEGVQGKGGEIDD.
Microcoleus c. PCC 7420 476 DGADIKDSFRNYERIISGSPSRITVDEKSELSRIVQGVRQKSTE....
Microcoleus v. FGP-2 475 RGPQIKDSFRGYERIISGSPSRITINEKSELSRILQGVQGQDEEDI..
Microcystis a. NIES-843 476 DGADIKDSFRNYERIISGSPTKISVDEKTELSRIVRNVREKLQDE...
Microcystis a. PCC 7806 476 DGADIKDSFRNYERIISGSPTKISVDEKTELSRIVRNVREKLQDE...
Microcystis a. PCC 7820 476 DGADIKDSFRNYERIISGSPTKISVDEKTELSRIVRNVREKLQDE...
Microcystis a. PCC 7941 476 DGADIKDSFRNYERIISGSPTKISVDEKSELSRIVRNVREKLQDE...
Microcystis a. PCC 9443 476 DGADIKDSFRNYERIISGSPTKISVDEKSELSRIVRNVREKLQDE...
Microcystis a. PCC 9701 476 DGADIKDSFRNYERIISGSPTKISVDEKTELSRIVRNVREKLQDE...
Microcystis a. PCC 9717 476 DGADIKDSFRNYERIISGSPTKISVDEKSELSRIVRNVREKLQDE...
Microcystis a. PCC 9806 476 DGADIKDSFRNYERIISGSPTKISVDEKTELSRIVKNVREKLQDE...
Microcystis a. PCC 9807 476 DGADIKDSFRNYERIISGSPTKISVDEKSELSRIVRNVREKLQDE...
Microcystis sp. T1-4 476 DGADIKDSFRNYERIISGSPTKISVDEKTELSRIVRNVREKLQDE...
Moorea p. 3L 476 DGPEIKDSFRNYERIISGSPTRIPVDEKSELSRIVKGVRDKSGE....
Nodularia s. CCY9414 474 DGPEIKDSFRNYERIISGAPTRVSIDEKAELSRIVQSFENKPSSDSSI
Nostoc a. 0708 474 DGPNIKDSFRNYERIVSGAPTRVIMDEKAELSRIVKRFEEK.......
Nostoc c. 474 DGPEIKDSFRNHQRIVNGAPTRVSIDEKAELSRIVKSFEYYRSSDS..
Nostoc p. PCC 73102 475 DGPDIKDSFRNYERIISGAPTRVSIDEKAELSRIVRRFEDKQSSEP..
Nostoc sp. PCC 7120 474 DGPEIQDSFRNYERIVSGSPTRVSIDEKAELSRIVRRFEDKQGSDS..
Nostoc sp. PCC 9709 475 DGPDIKDSFRNYERIISGAPTRVSIDEKAELSRIVRRFEDKQSSEP..
Oscillatoria sp. PCC 6506 475 KGPEIRDSFRSYERIISGSPTRISFNEKSELSRIIKGVQGQGGDGEEI
Raphidiopsis b. D9 477 DGPNIKDSFRNYERIVSGAPSRVSIDEKAELSRIVKRFEDK.......
Synechococcus e. PCC 6301 475 KGPDIKDSFRNFERIISGSPTRITVDEKSELSRIVRGVQEKGPES...
Synechococcus sp. PCC 7335 479 DGPQIQESFRNLEGIISGSARRIPSREKNELARIVSAVRSDDD.....
Synechococcus sp. BL107 460 NGPQIKDSFSNFERIISGVPHRVSTDERSELSRIAKSVSSDD......
Synechococcus sp. CB0101 471 NGPEIKDSFSNFERIISGVPHRINTDERSELSRIVQGVTSDDRL....
Synechococcus sp. CB0205 471 NGPEIKDSFSNFERIISGVPHRIDHDERSELSRIVKGVGEGQF.....
Synechococcus sp. CC9311 471 NGPQIQDSFSNFERIISGVPHRVTMDERSELSRIARGVSPE.......
Synechococcus sp. CC9605 471 NGPQIKDSFSNFERIISGVPHRVTTDERSELSRIARGVSTED......
Synechococcus sp. CC9902 471 NGPQIKDSFSNFERIISGVPHRVSTDERSELSRIARSVSSDD......
Synechococcus sp. JA-2-3B 496 AGIQIRDSFRGYERIISGSPTRINVDEKNELSRIVQNVQALEEEGL..
Synechococcus sp. JA-3-3Ab 492 SGIQIRDSFRGYERIISGSPTRINVDEKNELSRIVQNVQSLDE.....
Synechococcus sp. PCC 7002 472 GGAAIKDSFRNYERIISGSPTRIAVDEKSELSRIMRGVQDKTLPE...
Synechococcus sp. RCC307 475 NGPEIKDSFANFERIISGVPHRITTDERNELARIVRGVDSEPG.....
Synechococcus sp. RS9916 471 NGPEIKDSFSNFERIISGVPHRITTDERSELSRIVRGVNPDA......
Synechococcus sp. RS9917 478 NGPQIKDSFSNFERIISGVPHRVSTDERSELSRIVRGVTADD......
Synechococcus sp. WH 5701 471 NGPEIRDSFSNFERIISGVPHRVTTDERTELSRIARSFEGEESF....
Synechococcus sp. WH 7803 471 NGPQIKDSFSNFERIISGVPHRVTTDERSELLRIARGVDPDA......
Synechococcus sp. WH 7805 471 NGPQIKDSFSNFERIISGVPHRVTTDERSELLRIARGVDPDA......
Synechococcus sp. WH 8016 460 NGPQIKDSFSNFERIISGVPHRVTMDERSELSRIARGVAPE.......
Synechococcus sp. WH 8102 471 NGPQIKDSFSNFERIISGVPHRVTTDERSELSRIARGVSSED......
Synechococcus sp. WH 8109 460 NGPQIKDSFSNFERIISGVPHRVTTDERSELSRIARGVSTED......
Synechocystis sp. PCC 6803 476 DGPDIRDSFRNYERIISGSPTRISVDEKSELSRIVKGVKDKTAE....
Thermosynechococcus e. BP-1 475 KGAEIRDSFRNFEGIISGTPTRISVDEKTELARIAKGMQDLESE....
Trichodesmium e. IMS101 475 QGPDIRDSFRSYEGIISGSPTRTTVNEKSELSRIIQGVQEKTTDD...
consensus ! !***! ****!**! * *****!* *!*!!** *
logo

(D)

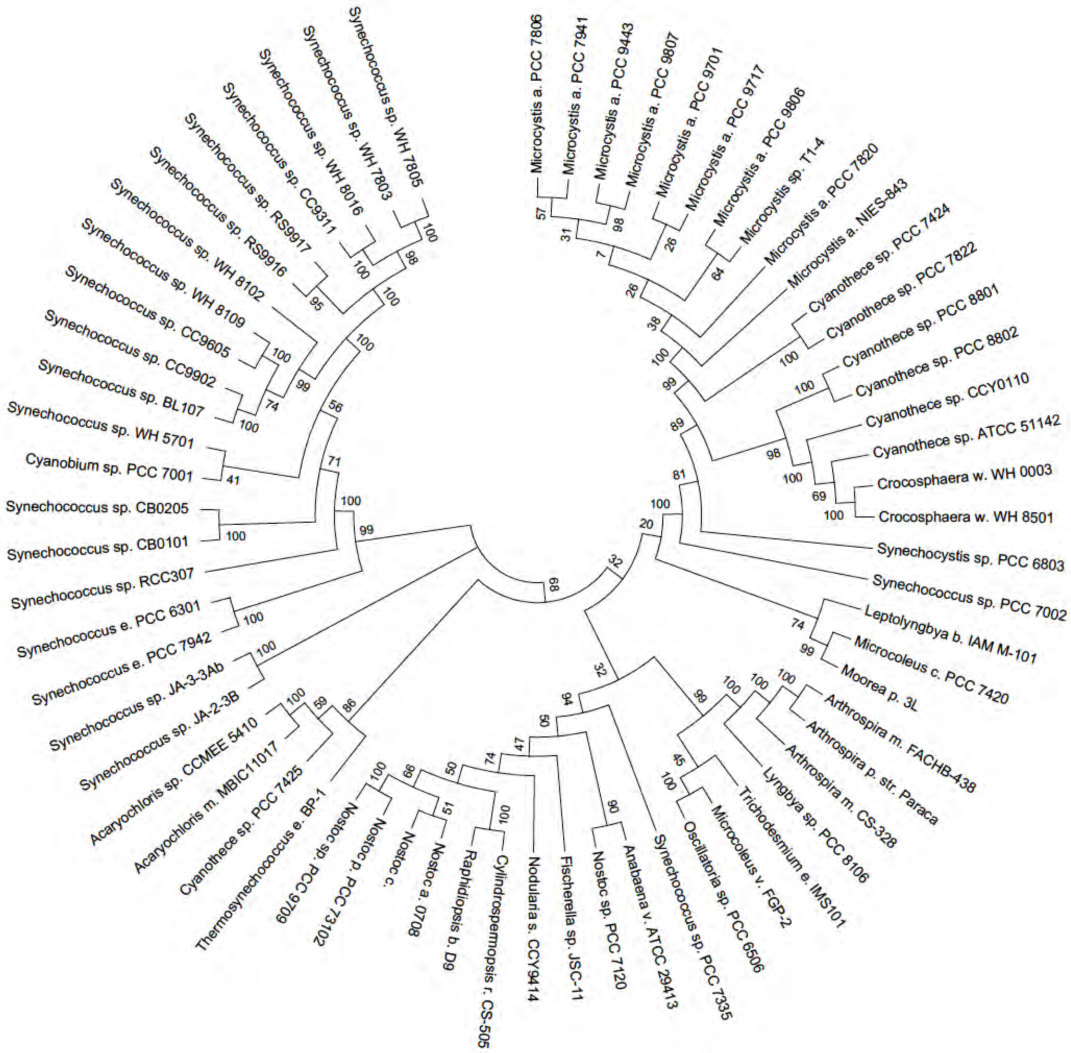
Microcystis a. PCC 7806
Microcystis a. PCC 7941
Microcystis a. PCC 9443
Microcystis a. PCC 9807
Microcystis a. PCC 9701
Microcystis a. PCC 9717
Microcystis a. PCC 9806
Microcystis sp. T1-4
Microcystis a. PCC 7820
Microcystis a. NIES-843
Cyanothece sp. PCC 7424
Cyanothece sp. PCC 7822
Cyanothece sp. PCC 8801
Cyanothece sp. PCC 8802
Cyanothece sp. CCY0110
Cyanothece sp. ATCC 51142
Crocosphaera w. WH 0003
Crocosphaera w. WH 8501
Synechocystis sp. PCC 6803
Synechococcus sp. PCC 7002
Leptolyngbya b. IAM M-101
Microcoleus c. PCC 7420
Moorea p. 3L
Arthrospira m. FACHB-438
Arthrospira p. str. Paraca
Arthrospira m. CS-328
Lyngbya sp. PCC 8106
Trichodesmium e. IMS101
Microcoleus v. FGP-2
Oscillatoria sp. PCC 6506
Synechococcus sp. PCC 7335
Anabaena v. ATCC 29413
Nostoc sp. PCC 7120
Fischerella sp. JSC-11
Nodularia s. CCY9414
Cylindrospermopsis r. CS-505
Raphidiopsis b. D9
Nostoc a. 0708
Nostoc c.
Nostoc p. PCC 73102
Nostoc sp. PCC 9709
Thermosynechococcus e. BP-1
Cyanothece sp. PCC 7425
Acaryochloris m. MBIC11017
Acaryochloris sp. CCMEE 5410
Synechococcus sp. JA-2-3B
Synechococcus sp. JA-3-3Ab
Synechococcus e. PCC 7942
Synechococcus e. PCC 6301
Synechococcus sp. RCC307
Synechococcus sp. CB0101
Synechococcus sp. CB0205
Cyanobium sp. PCC 7001
Synechococcus sp. WH 5701
Synechococcus sp. BL107
Synechococcus sp. CC9902
Synechococcus sp. CC9605
Synechococcus sp. WH 8109
Synechococcus sp. WH 8102
Synechococcus sp. RS9916
Synechococcus sp. RS9917
Synechococcus sp. CC9311
Synechococcus sp. WH 8016
Synechococcus sp. WH 7803
Synechococcus sp. WH 7805

(E)

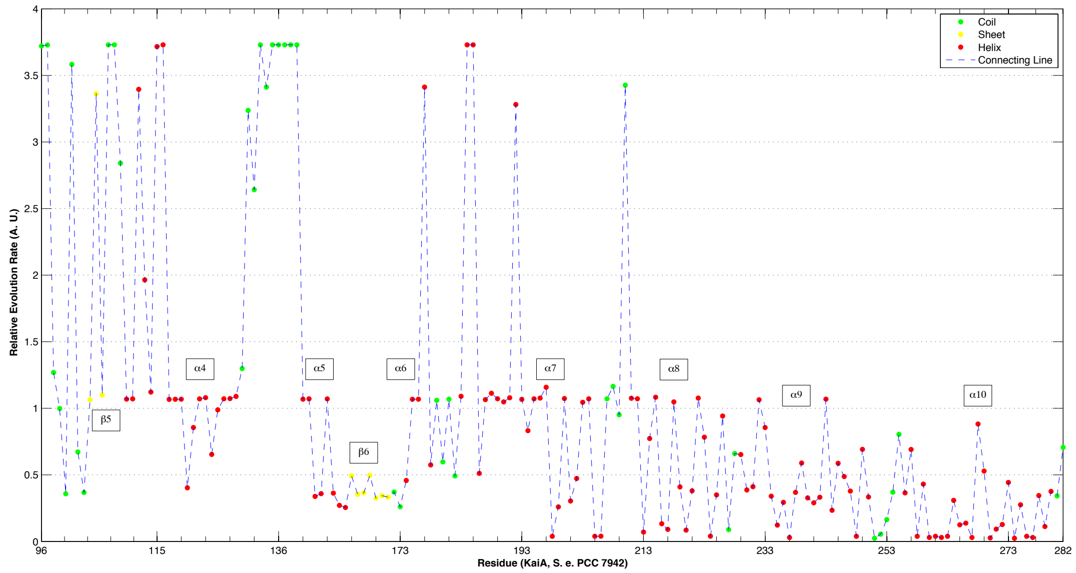
Coil
Sheet
Helix
Connecting Line
Relative Evolution Rate (A. U.)
Residue (KaiA, S. e. PCC 7942)
β5
α4
α5
β6
α6
α7
α8
α9
α10
0
0.5
1
1.5
2
2.5
3
3.5
4
96
115
136
153
173
193
213
233
253
273
282

(F)

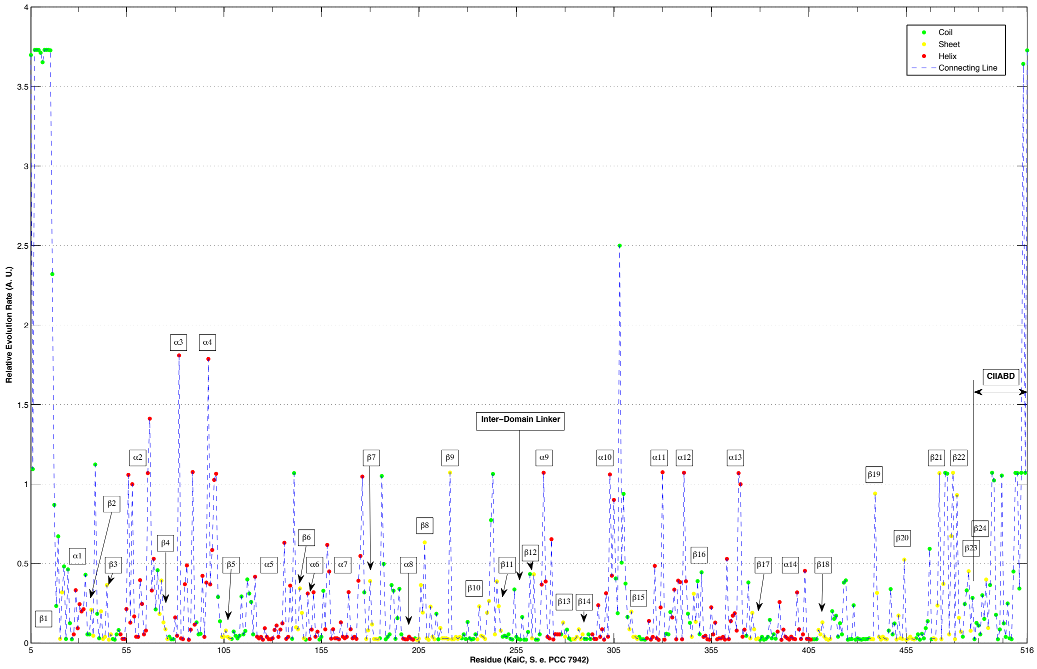

Coil
Sheet
Helix
Connecting Line
Relative Evolution Rate (A. U.)
Residue (KaiC, S. e. PCC 7942)
Inter-Domain Linker
CIIABD

Supplementary Figure S2. The molecular dynamics simulation of the apo structure of KaiA C-terminal domains (1Q6B). (A) The backbone RMSD variation of the whole C-terminal domains compared to the apo structure (1Q6B) and the holo structure (1SV1). (B) The backbone RMSD variation of the helices 9 and 10 compared to the apo structure (1Q6B) and the holo structure (1SV1) respectively. Compared to the simulation of the holo structure described in the main text, the conformational change of the apo structure had a lower oscillation frequency between these two conformations.

(A)

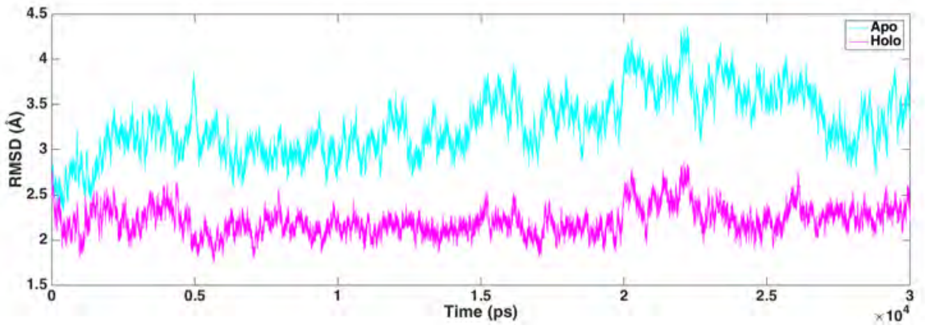

(B)

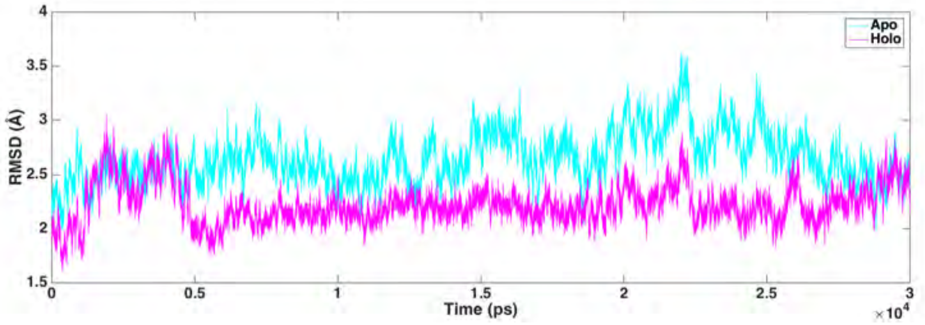

Supplementary Video S1. The snapshots of the molecular dynamics simulation of the apo structure of KaiA C-terminal domains. The apo structure (1R6B, in cyan) and the holo structure (1SV1, in magenta) are shown for comparison. Only 20 ns simulations were shown.

Supplementary Video S2. The snapshots of the molecular dynamics simulation of the holo structure of KaiA C-terminal domains. The apo structure (1R6B, in cyan) and the holo structure (1SV1, in magenta) are shown for comparison. Only 20 ns simulations were shown.
